# Supplementary figures and images for: Super-enhancer-driven ZFP36L1 promotes PD-L1 expression in infiltrative gastric cancer (part 1 of 2)
Source: eLife. 2024 Oct 7;13:RP96445. doi: 10.7554/eLife.96445 (PMC11458174; doi:10.7554/eLife.96445)

**Fig.2H**

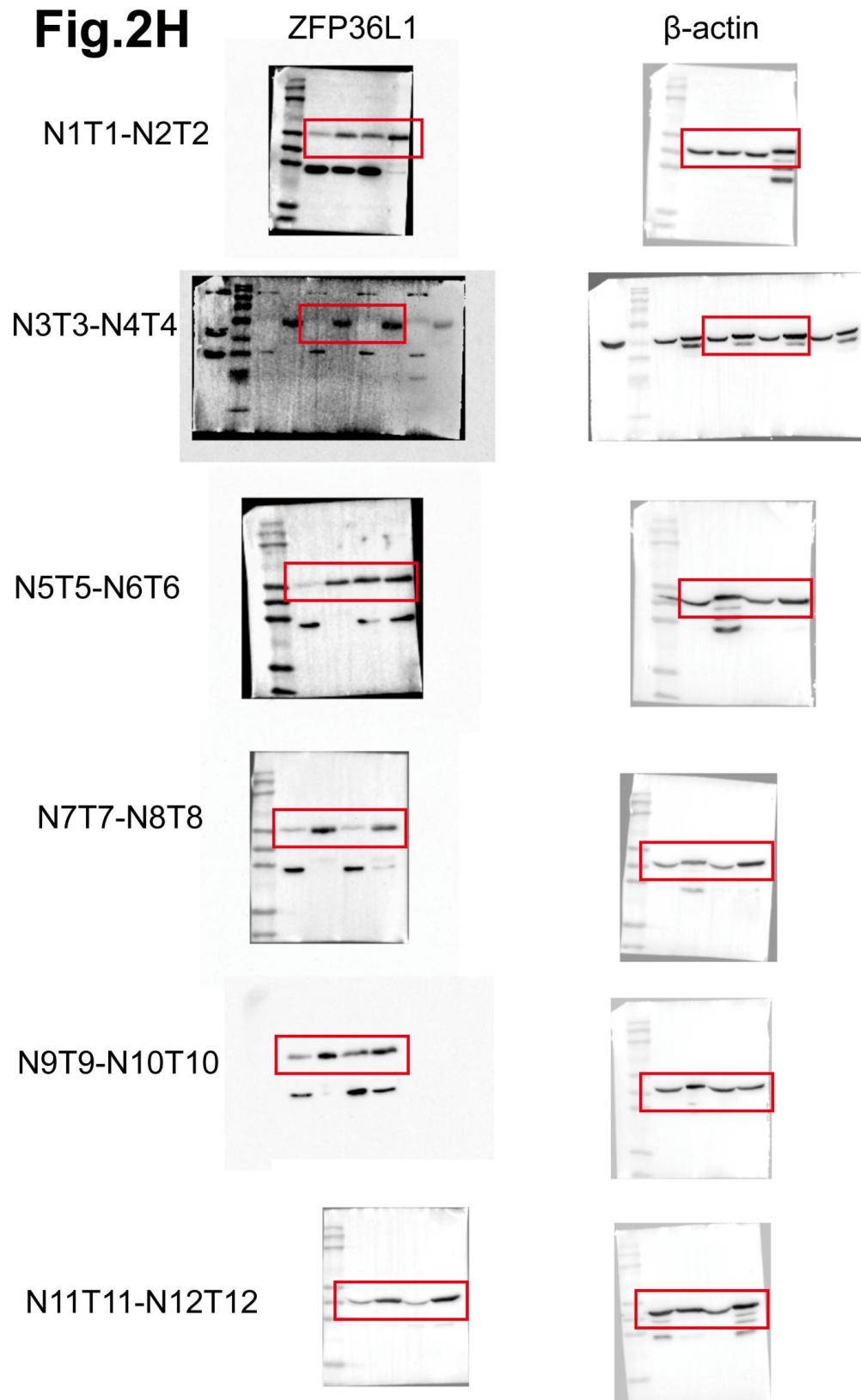

**Fig.2I**

ZFP36L1

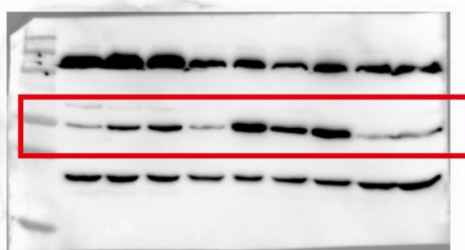

$\beta$ -actin

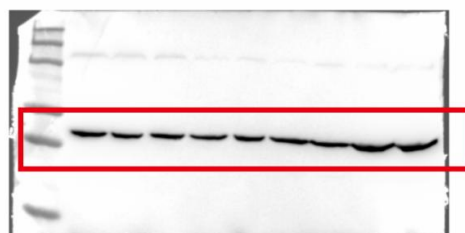

**Fig.2J**

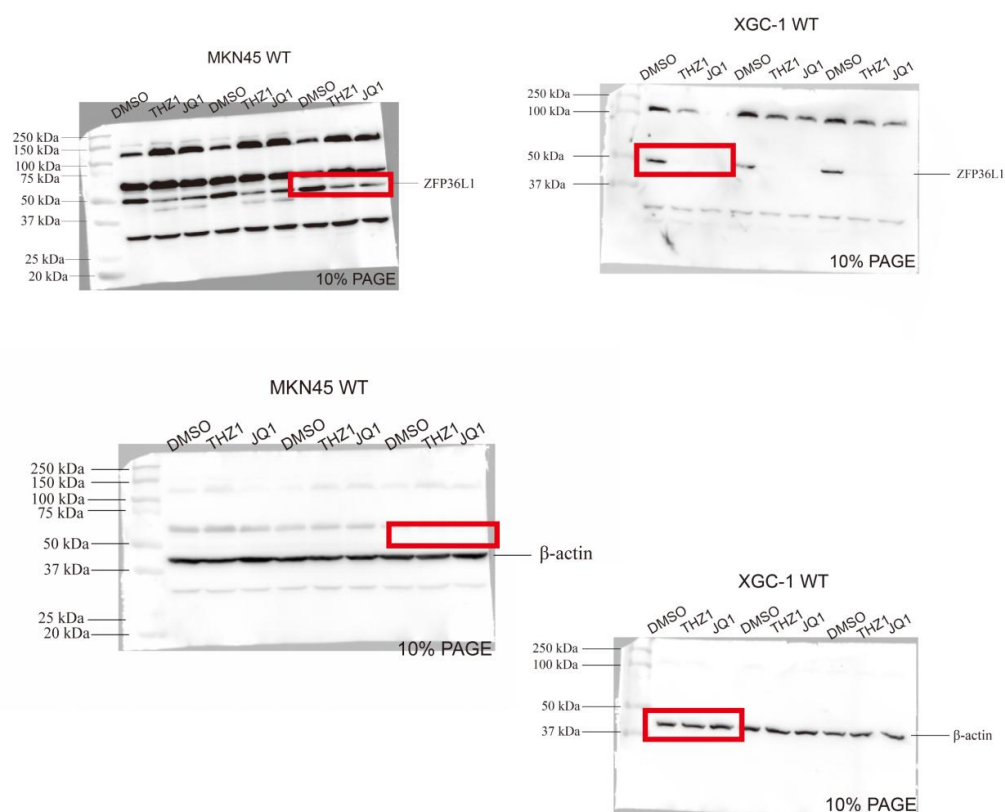

**Figure 2, Source Data 1.** Original membranes corresponding to Figure 2

Supplement: Figure 2—source data 1. [file elife-96445-fig2-data1.pdf]

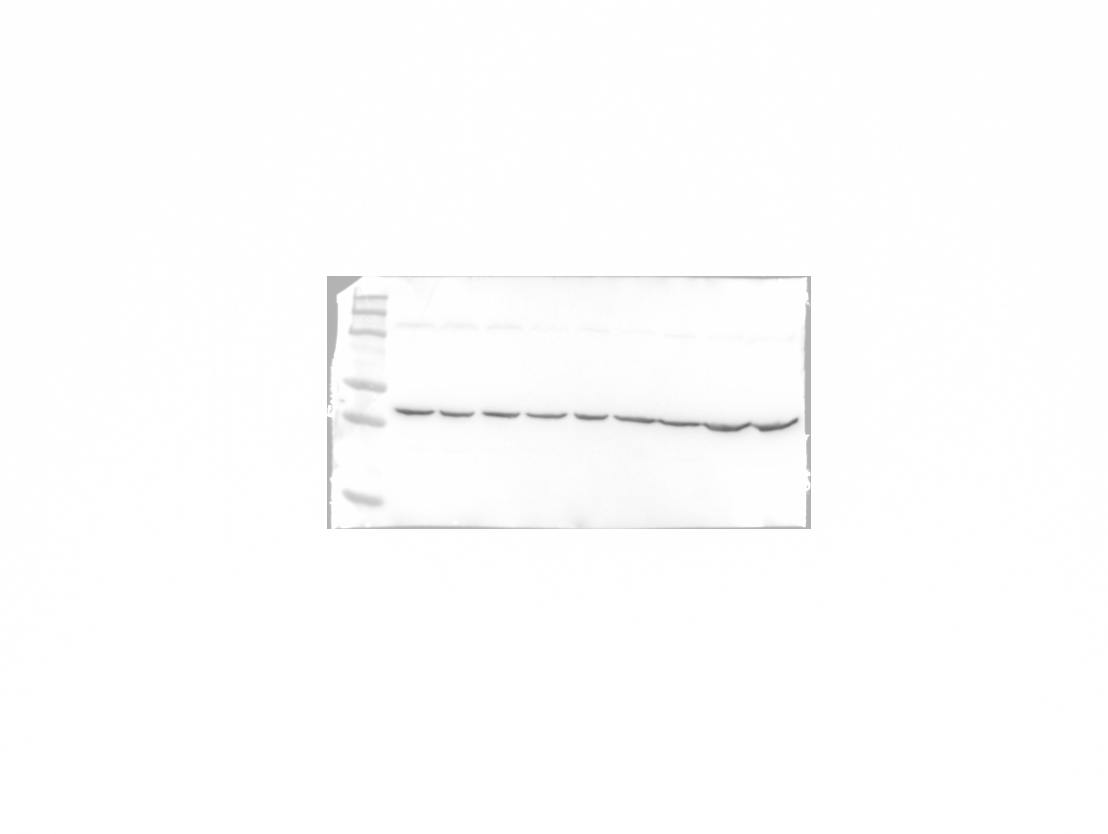

Supplement: Figure 2—source data 2. [file elife-96445-fig2-data2.zip › Figure 2_Source Data 2/Fig.2H actin.tif]

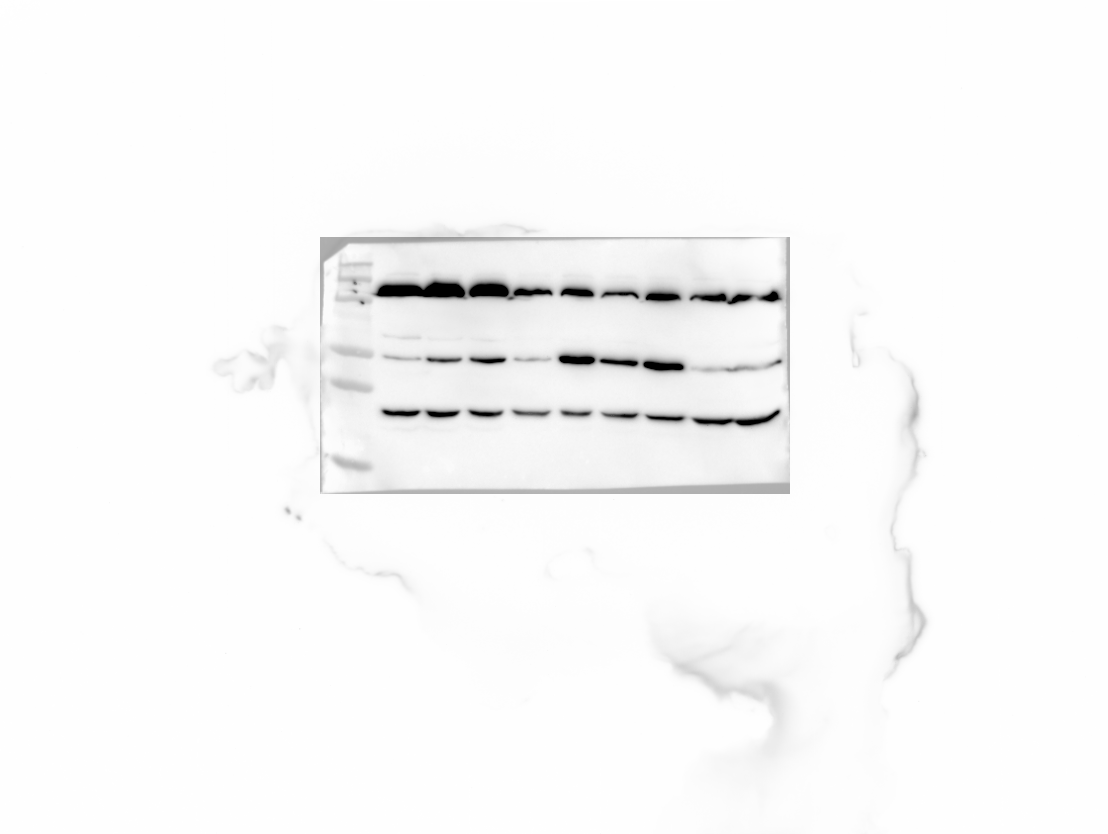

Supplement: Figure 2—source data 2. [file elife-96445-fig2-data2.zip › Figure 2_Source Data 2/Fig.2H ZFP36L1.tif]

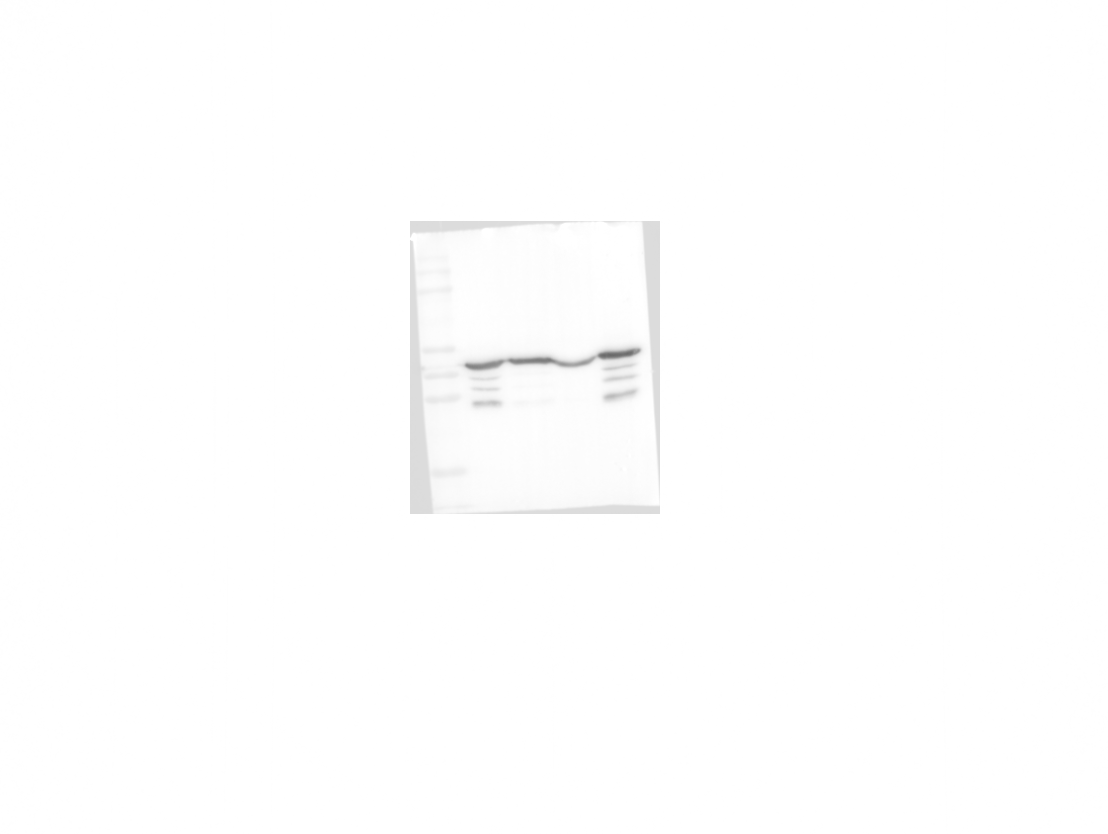

Supplement: Figure 2—source data 2. [file elife-96445-fig2-data2.zip › Figure 2_Source Data 2/Fig.2I N11N12-actin.tif]

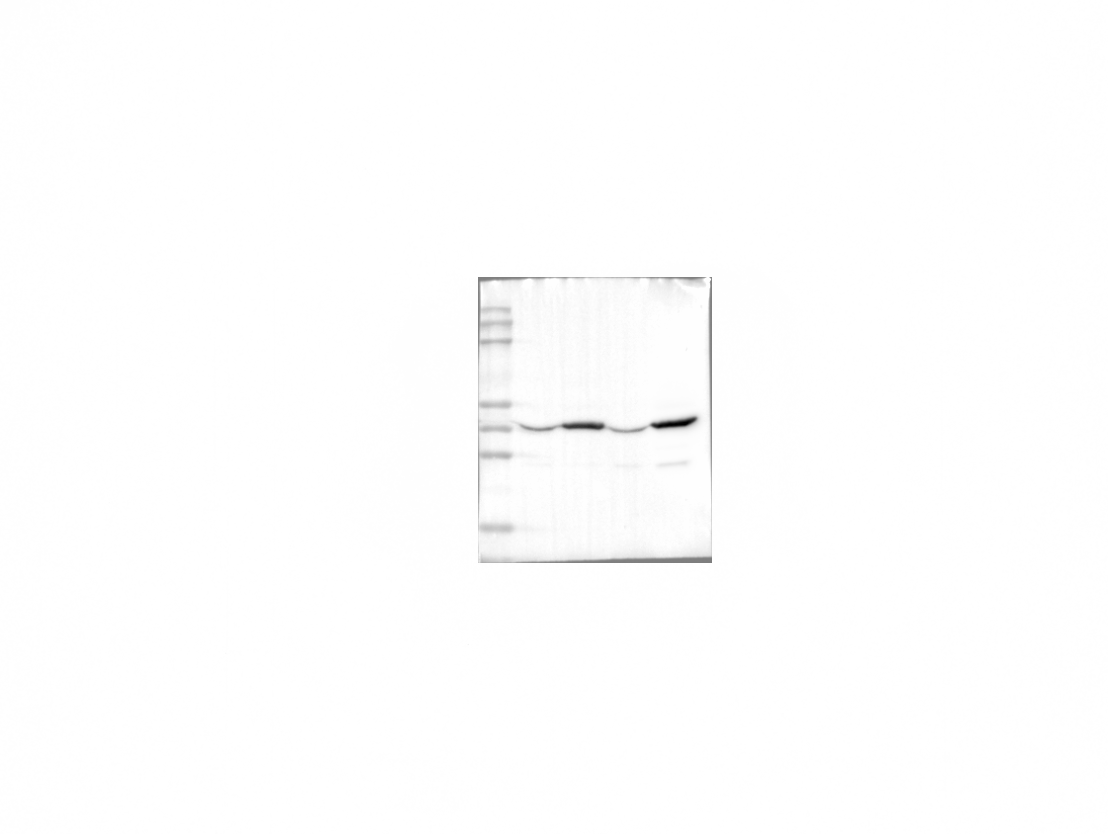

Supplement: Figure 2—source data 2. [file elife-96445-fig2-data2.zip › Figure 2_Source Data 2/Fig.2I N11N12-ZFP36L1.tif]

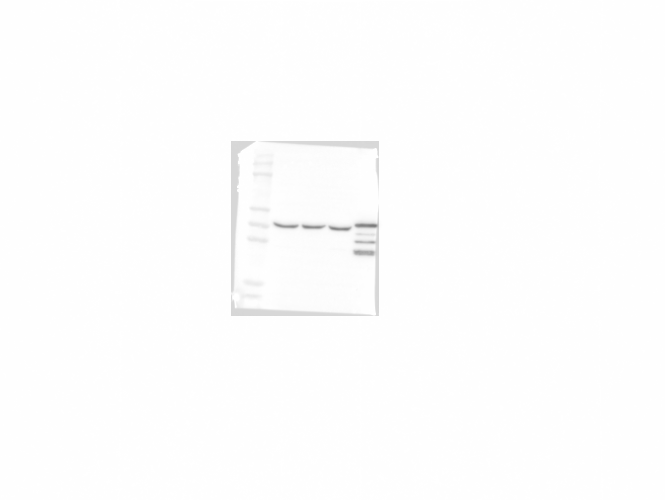

Supplement: Figure 2—source data 2. [file elife-96445-fig2-data2.zip › Figure 2_Source Data 2/Fig.2I N1N2-actin.tif]

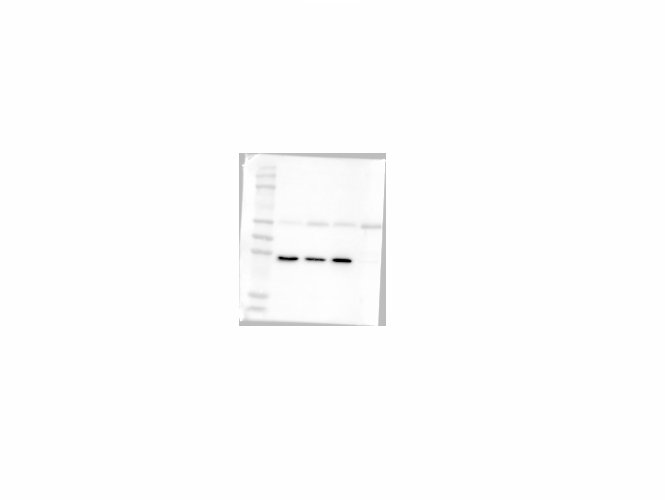

Supplement: Figure 2—source data 2. [file elife-96445-fig2-data2.zip › Figure 2_Source Data 2/Fig.2I N1N2-ZFP36L1.tif]

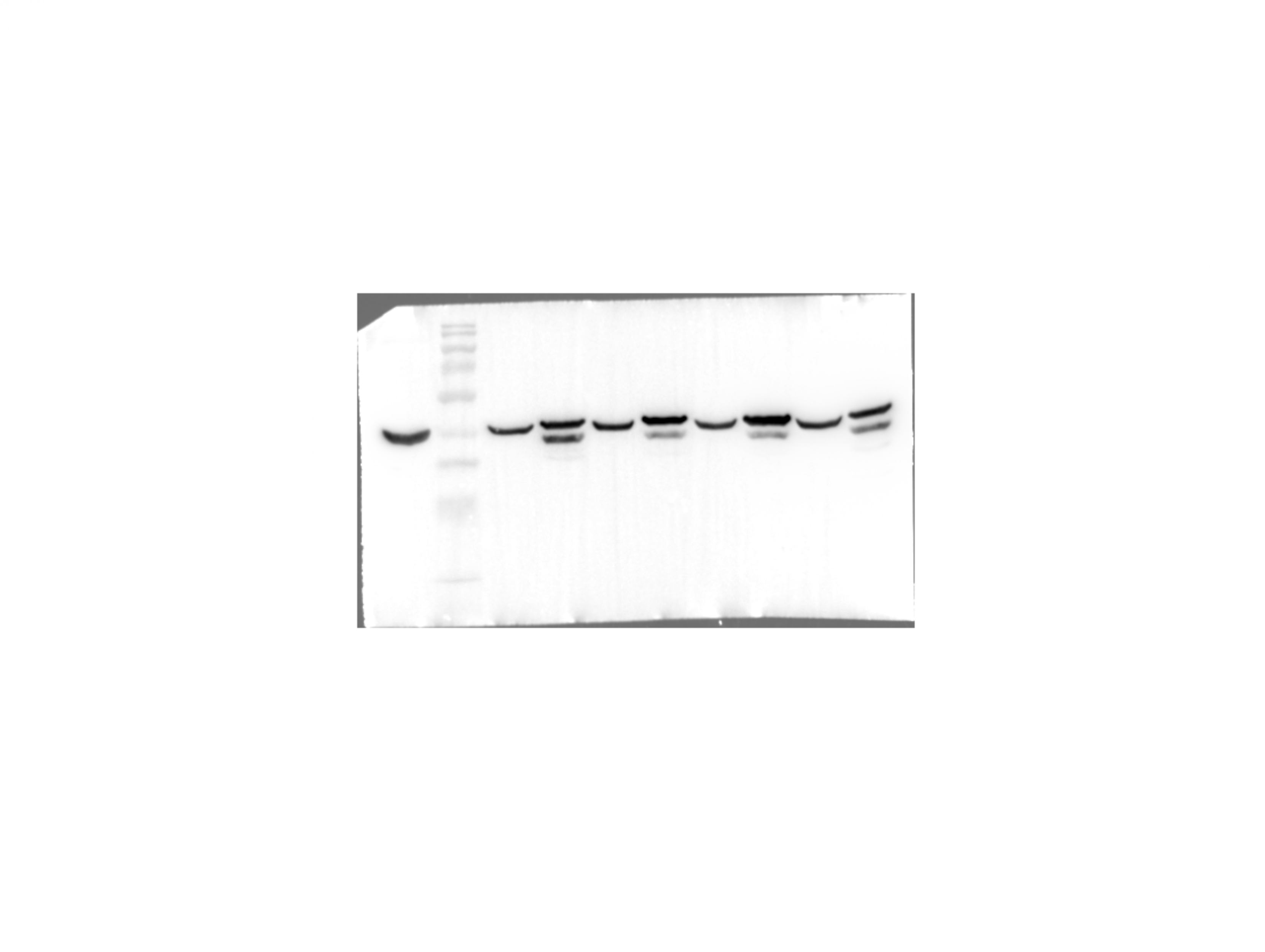

Supplement: Figure 2—source data 2. [file elife-96445-fig2-data2.zip › Figure 2_Source Data 2/Fig.2I N3N4-actin.tif]

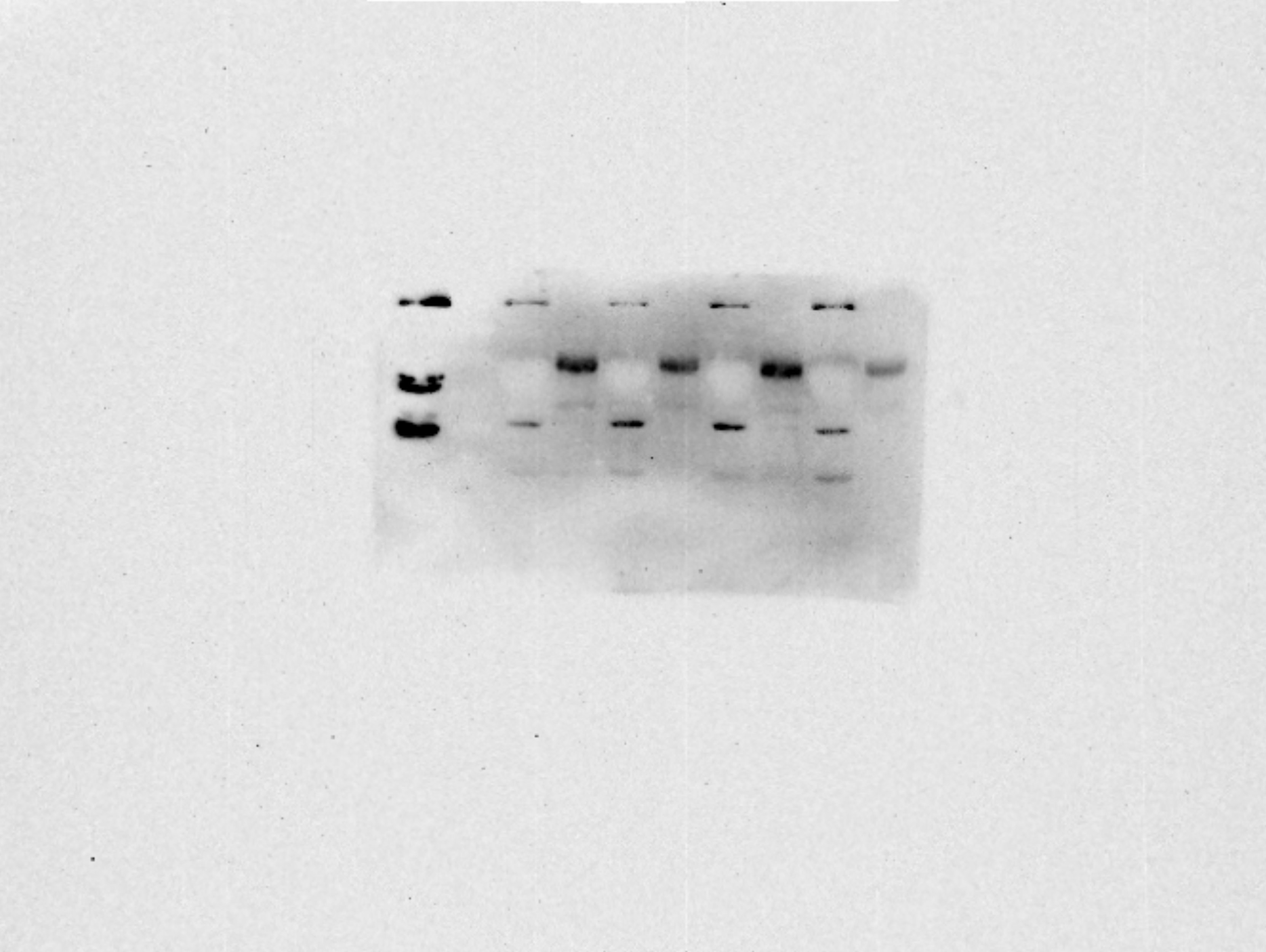

Supplement: Figure 2—source data 2. [file elife-96445-fig2-data2.zip › Figure 2_Source Data 2/Fig.2I N3N4-ZFP36L1.tif]

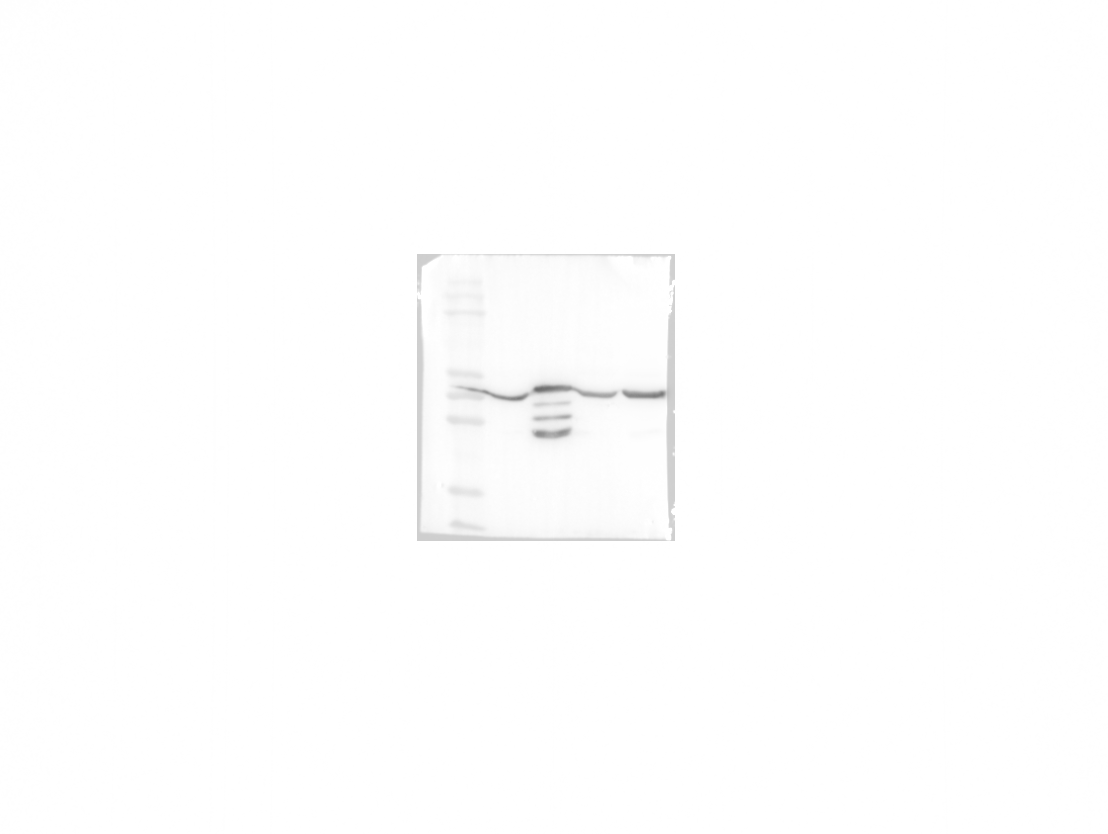

Supplement: Figure 2—source data 2. [file elife-96445-fig2-data2.zip › Figure 2_Source Data 2/Fig.2I N5N6-actin.tif]

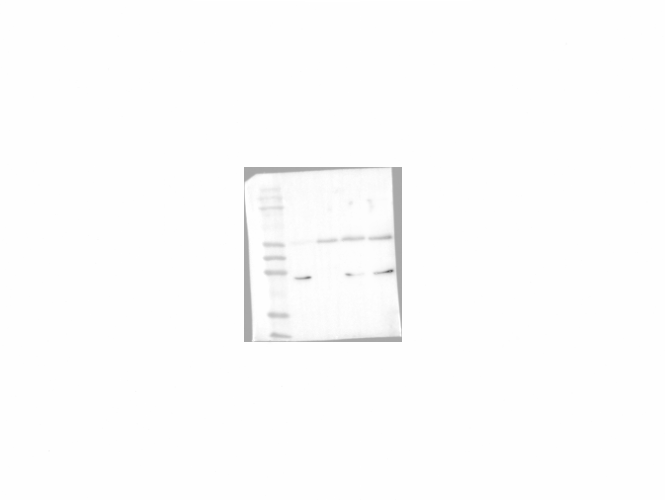

Supplement: Figure 2—source data 2. [file elife-96445-fig2-data2.zip › Figure 2_Source Data 2/Fig.2I N5N6-ZFP36L1.tif]

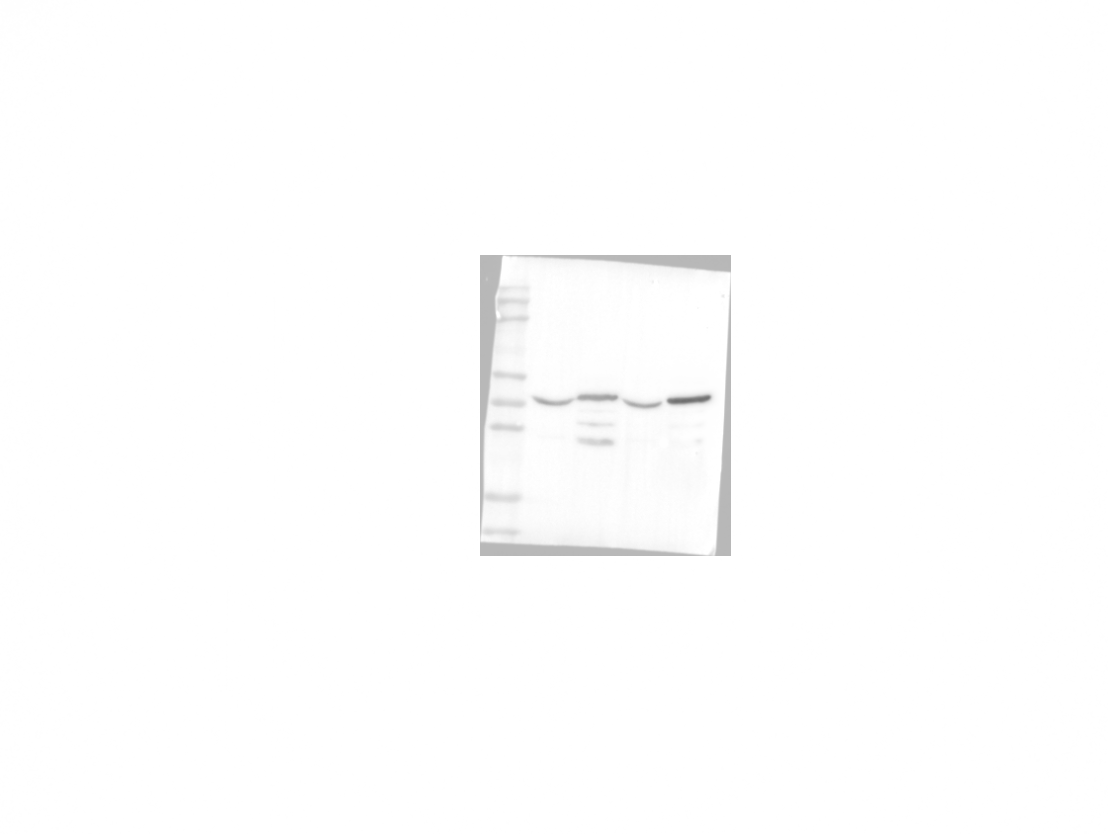

Supplement: Figure 2—source data 2. [file elife-96445-fig2-data2.zip › Figure 2_Source Data 2/Fig.2I N7N8-actin.tif]

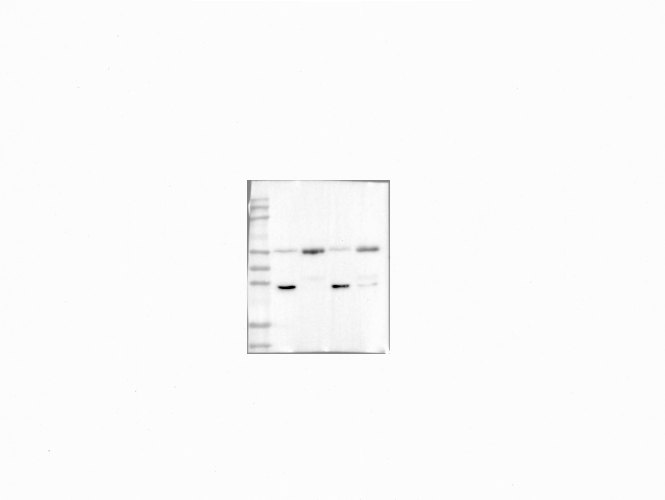

Supplement: Figure 2—source data 2. [file elife-96445-fig2-data2.zip › Figure 2_Source Data 2/Fig.2I N7N8-ZFP36L1.tif]

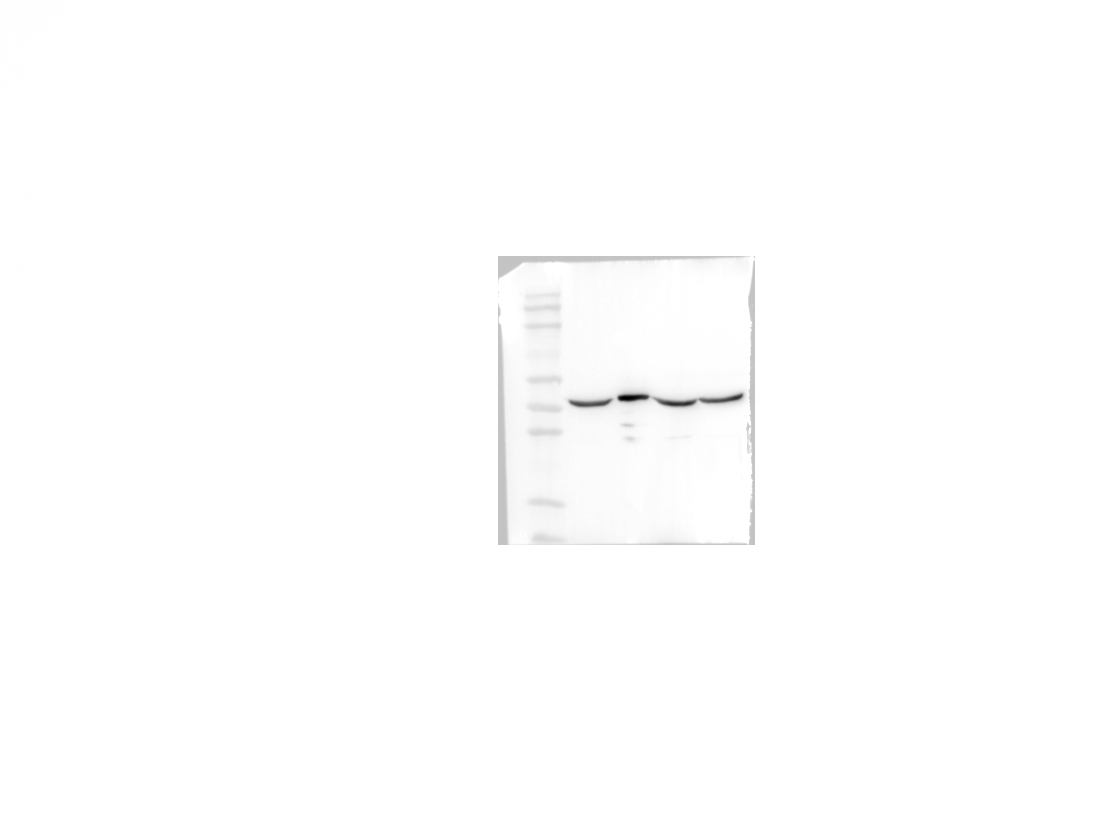

Supplement: Figure 2—source data 2. [file elife-96445-fig2-data2.zip › Figure 2_Source Data 2/Fig.2I N9N10-actin.tif]

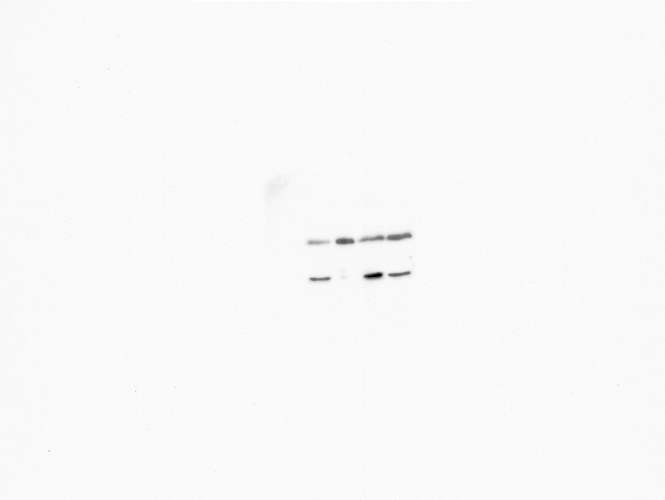

Supplement: Figure 2—source data 2. [file elife-96445-fig2-data2.zip › Figure 2_Source Data 2/Fig.2I N9N10-ZFP36L1.tif]

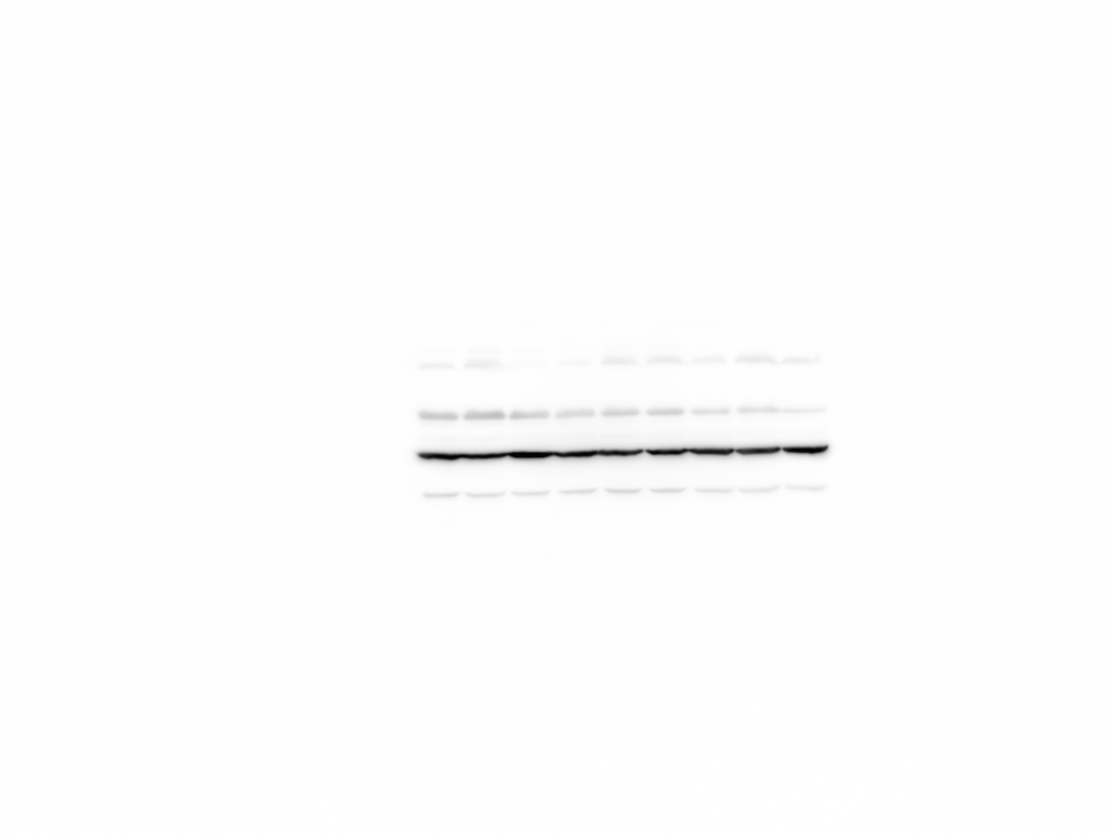

Supplement: Figure 2—source data 2. [file elife-96445-fig2-data2.zip › Figure 2_Source Data 2/Fig.2j actin MKN45.tif]

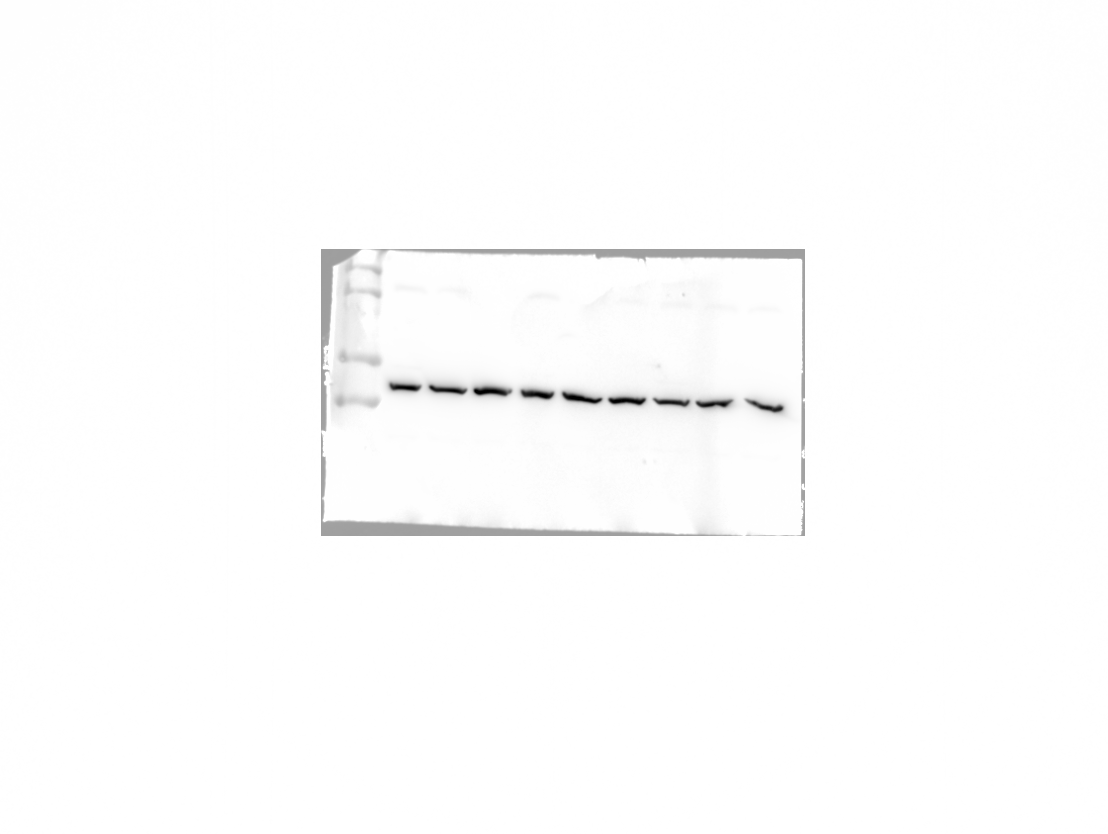

Supplement: Figure 2—source data 2. [file elife-96445-fig2-data2.zip › Figure 2_Source Data 2/Fig.2j actin XGC-1.tif]

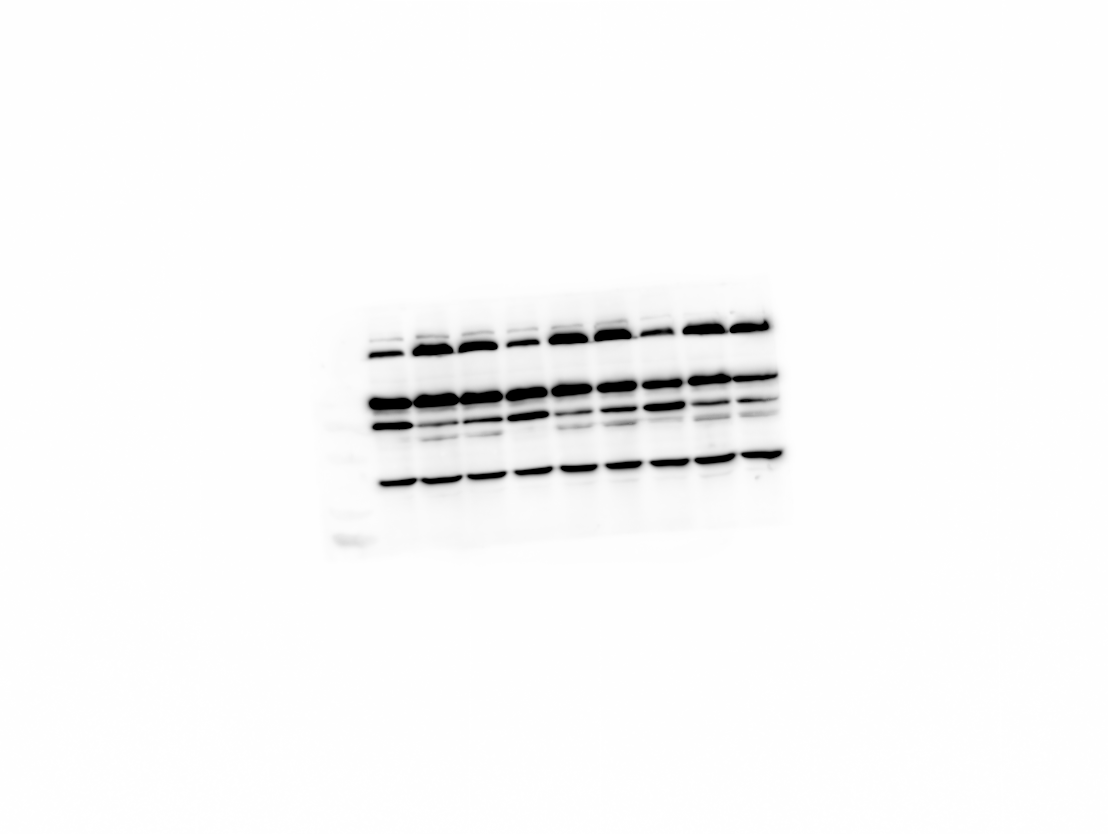

Supplement: Figure 2—source data 2. [file elife-96445-fig2-data2.zip › Figure 2_Source Data 2/Fig.2j ZFP36L1 MKN45.tif]

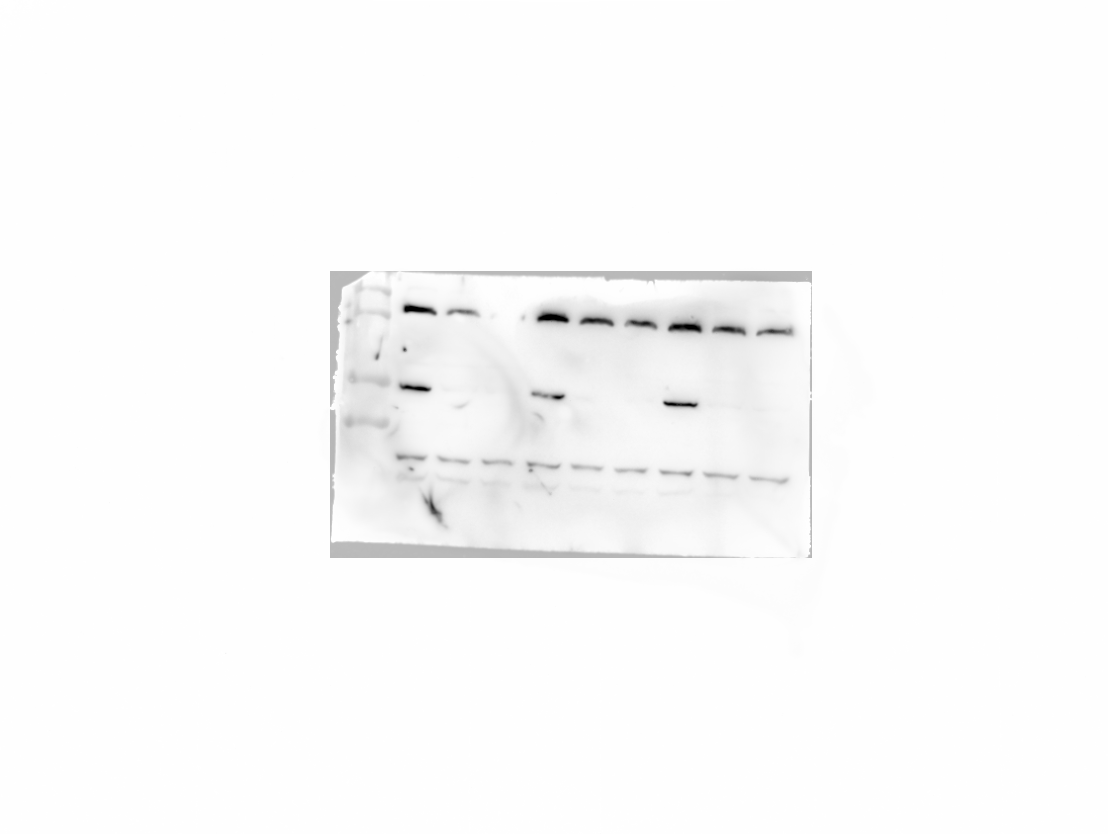

Supplement: Figure 2—source data 2. [file elife-96445-fig2-data2.zip › Figure 2_Source Data 2/Fig.2j ZFP36L1 XGC-1.tif]

# Fig.3C

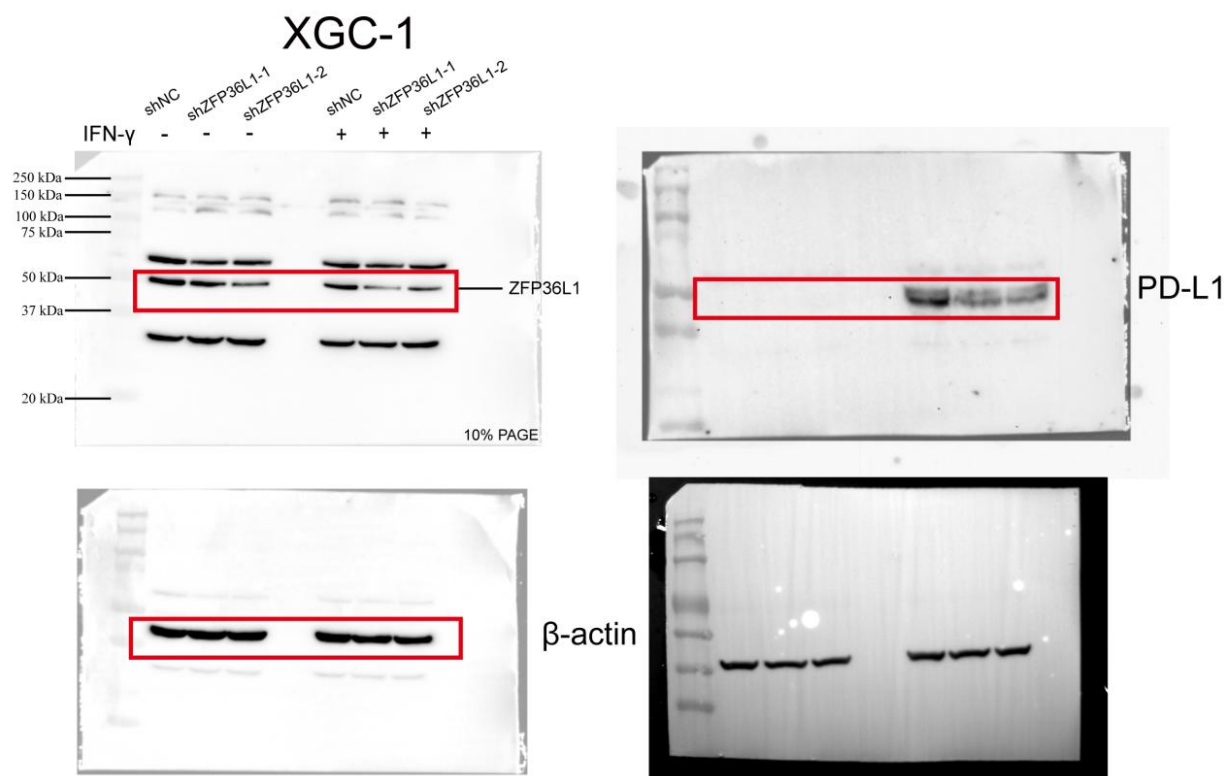

**MKN45**

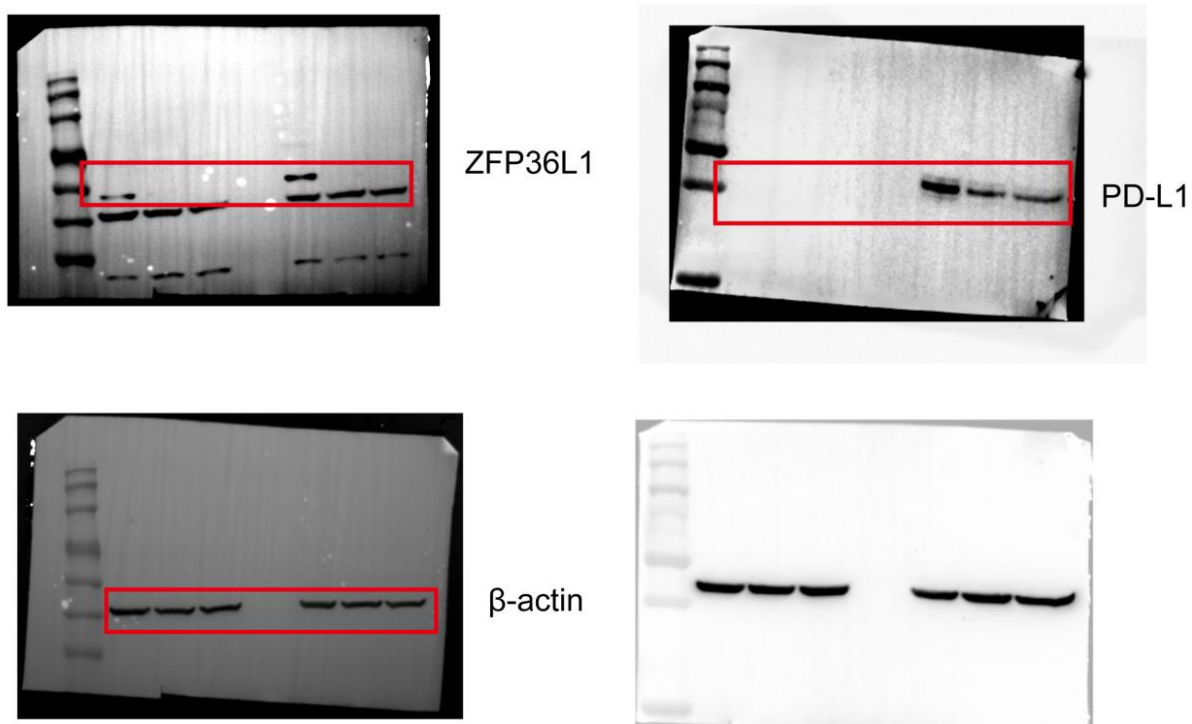

**Fig.3D**

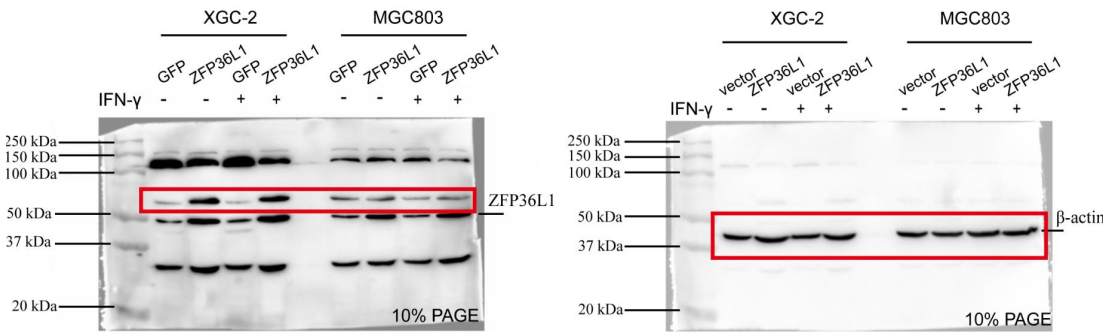

**Figure 3, Source Data 1.** Original membranes corresponding to Figure 3

Supplement: Figure 3—source data 1. [file elife-96445-fig3-data1.pdf]

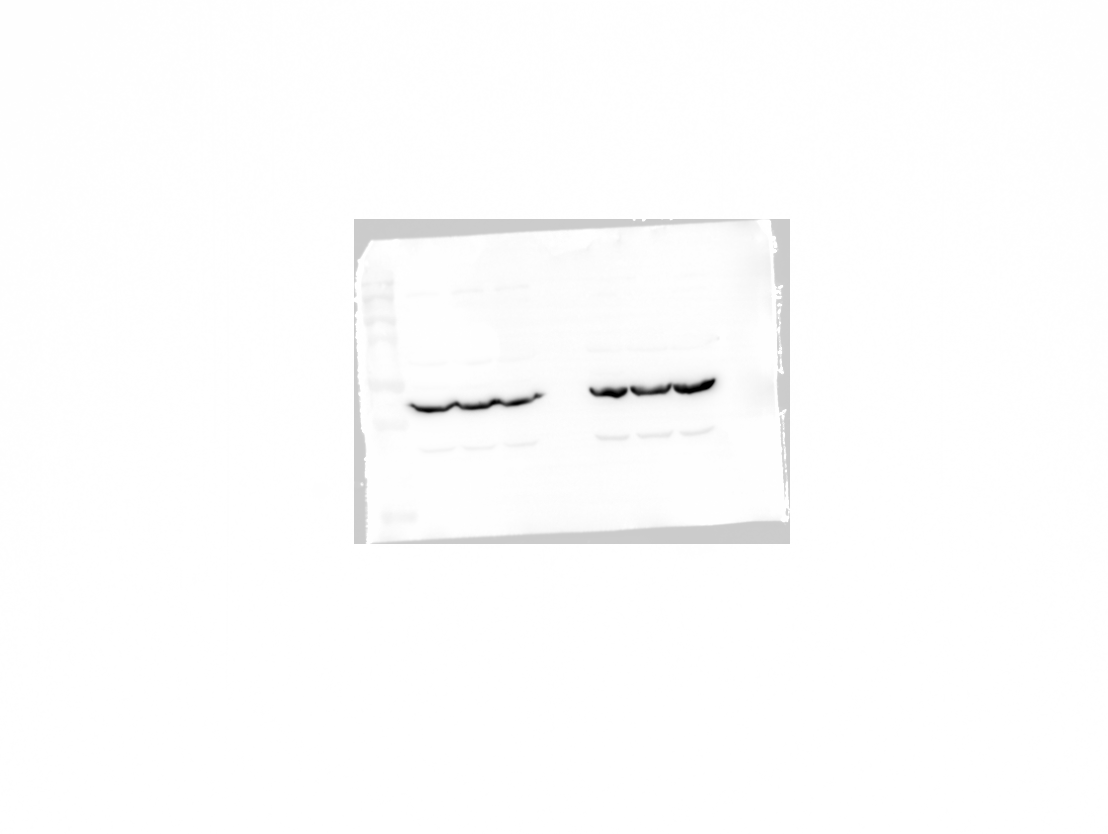

Supplement: Figure 3—source data 2. [file elife-96445-fig3-data2.zip › Figure 3_Source Data 2/Fig.3C MKN45-actin.tif]

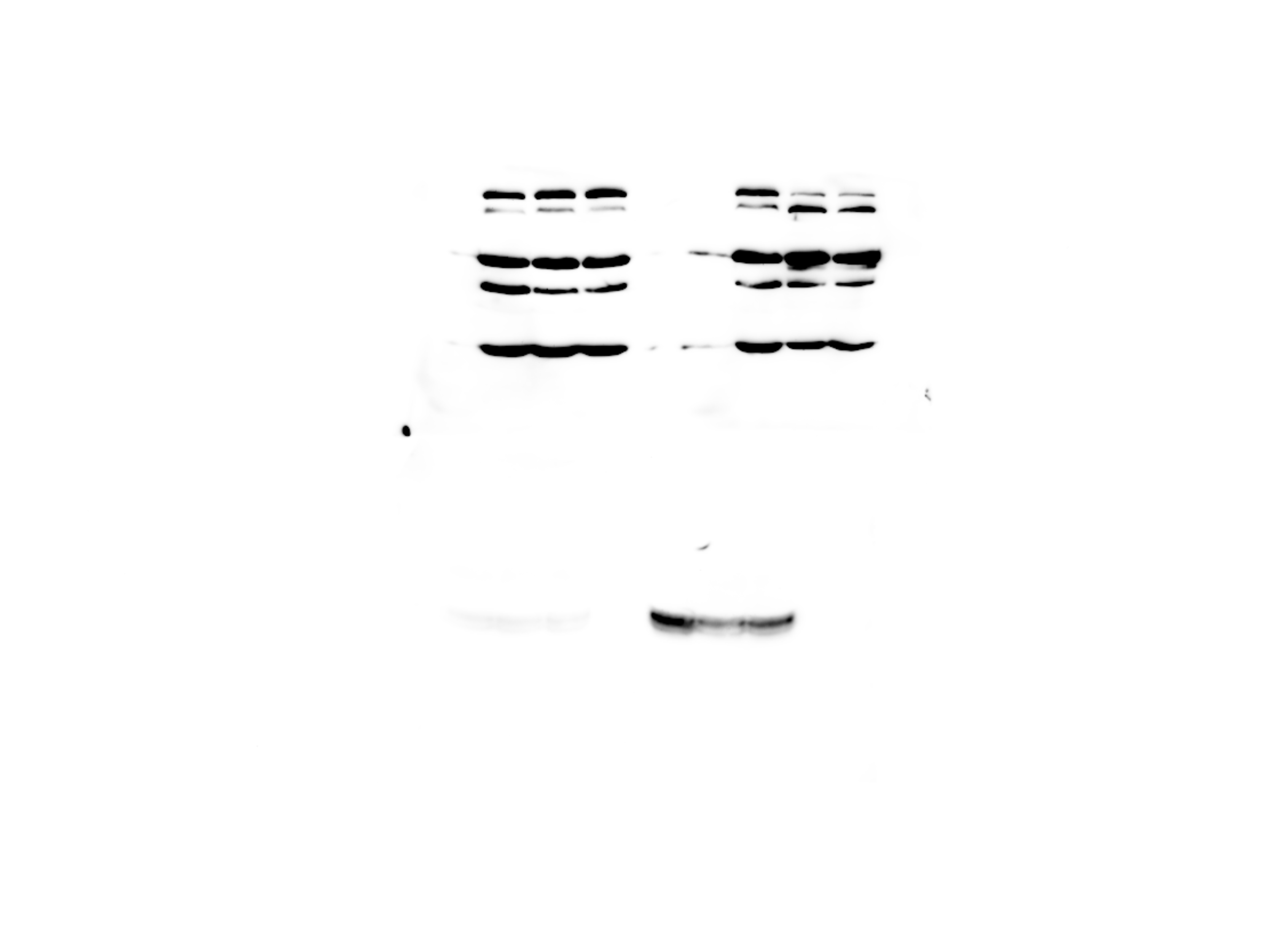

Supplement: Figure 3—source data 2. [file elife-96445-fig3-data2.zip › Figure 3_Source Data 2/Fig.3C MKN45-PD-L1.tif]

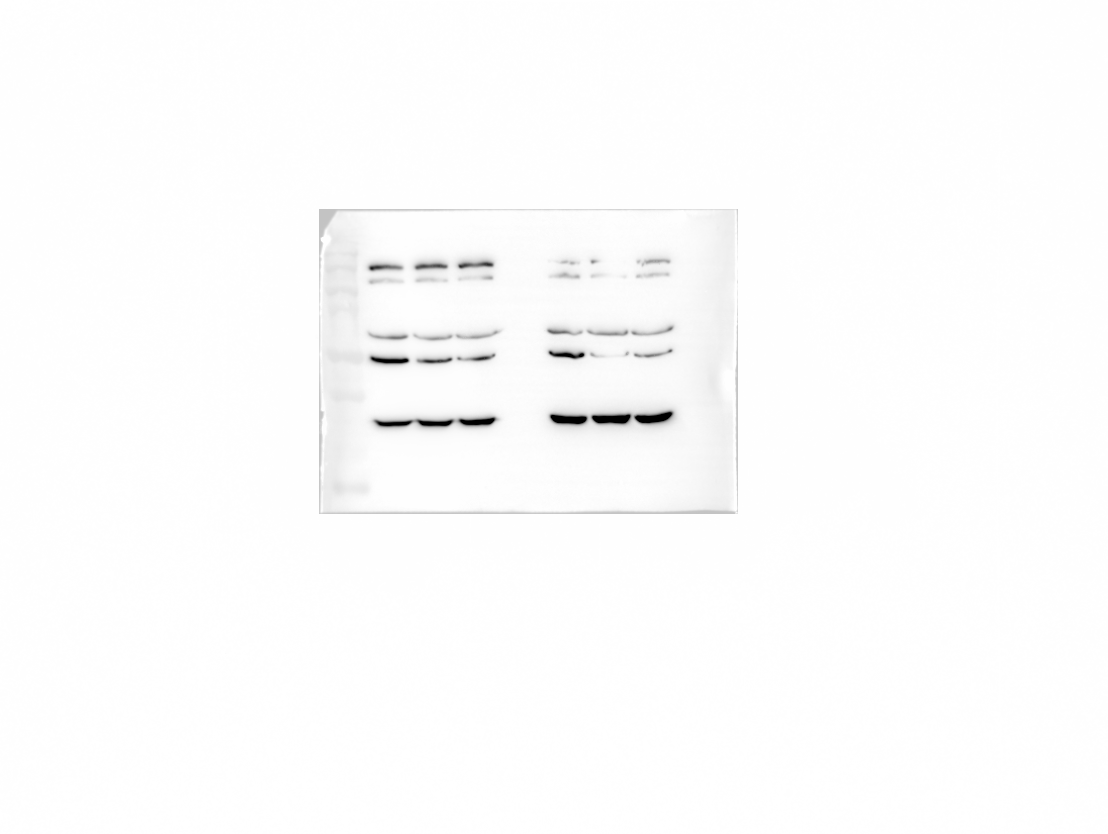

Supplement: Figure 3—source data 2. [file elife-96445-fig3-data2.zip › Figure 3_Source Data 2/Fig.3C MKN45-ZFP36L1.tif]

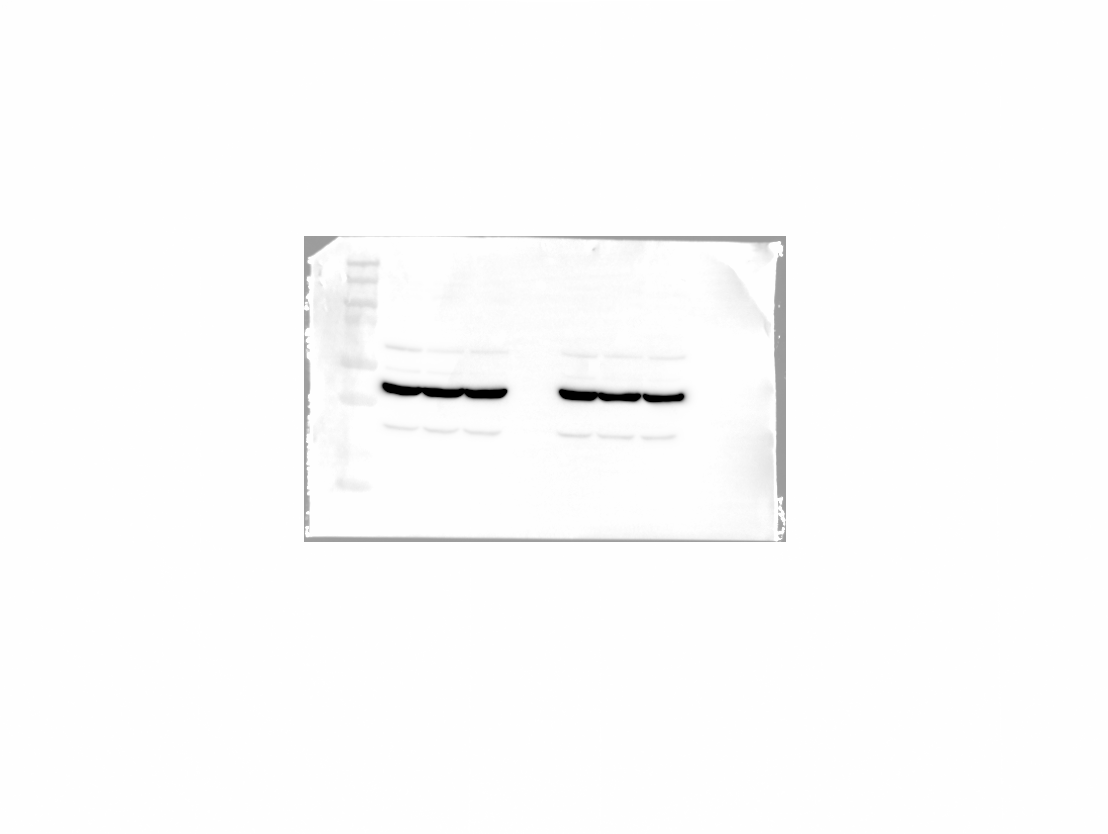

Supplement: Figure 3—source data 2. [file elife-96445-fig3-data2.zip › Figure 3_Source Data 2/Fig.3C XGC1-actin.tif]

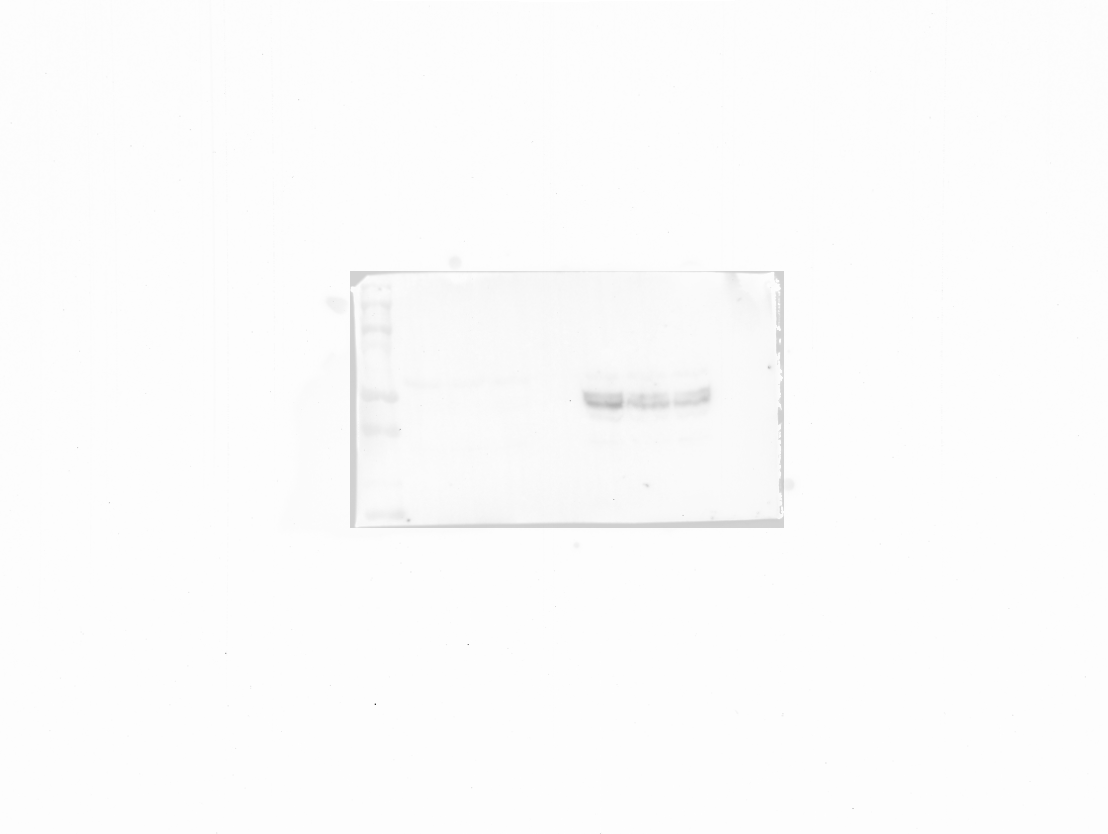

Supplement: Figure 3—source data 2. [file elife-96445-fig3-data2.zip › Figure 3_Source Data 2/Fig.3C XGC1-PDL1.tif]

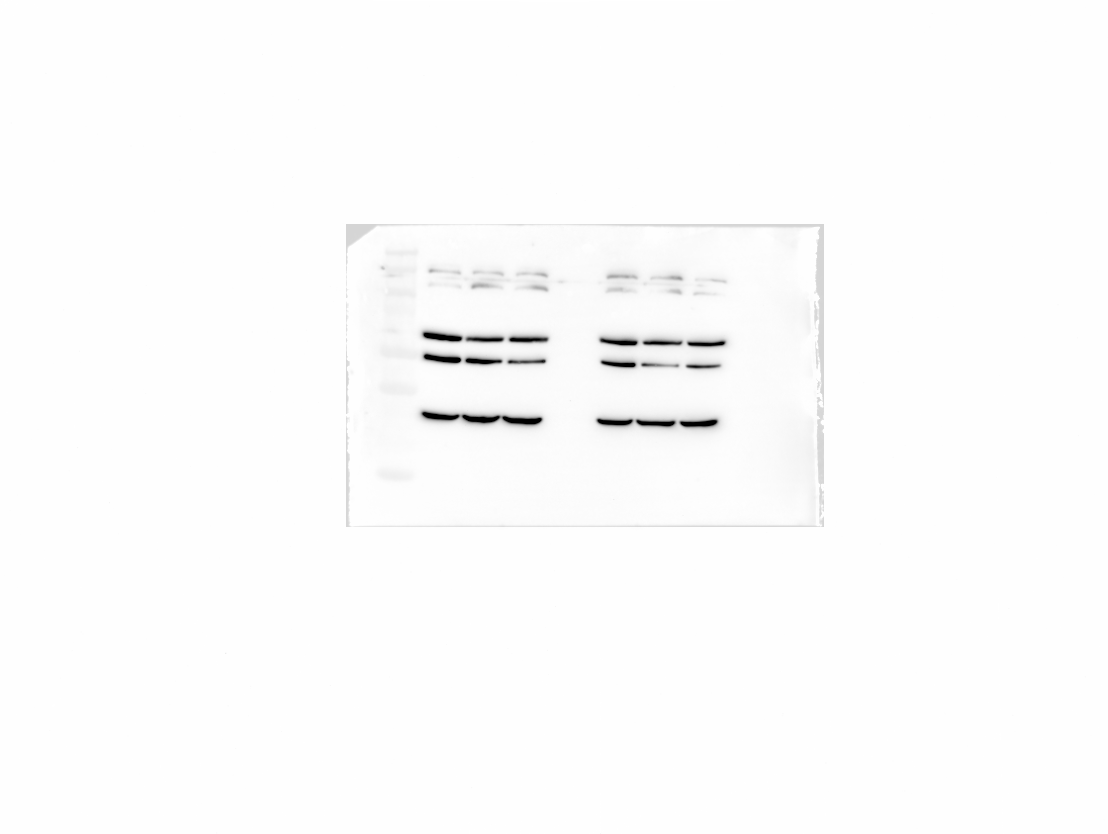

Supplement: Figure 3—source data 2. [file elife-96445-fig3-data2.zip › Figure 3_Source Data 2/Fig.3C XGC1-ZFP36L1.tif]

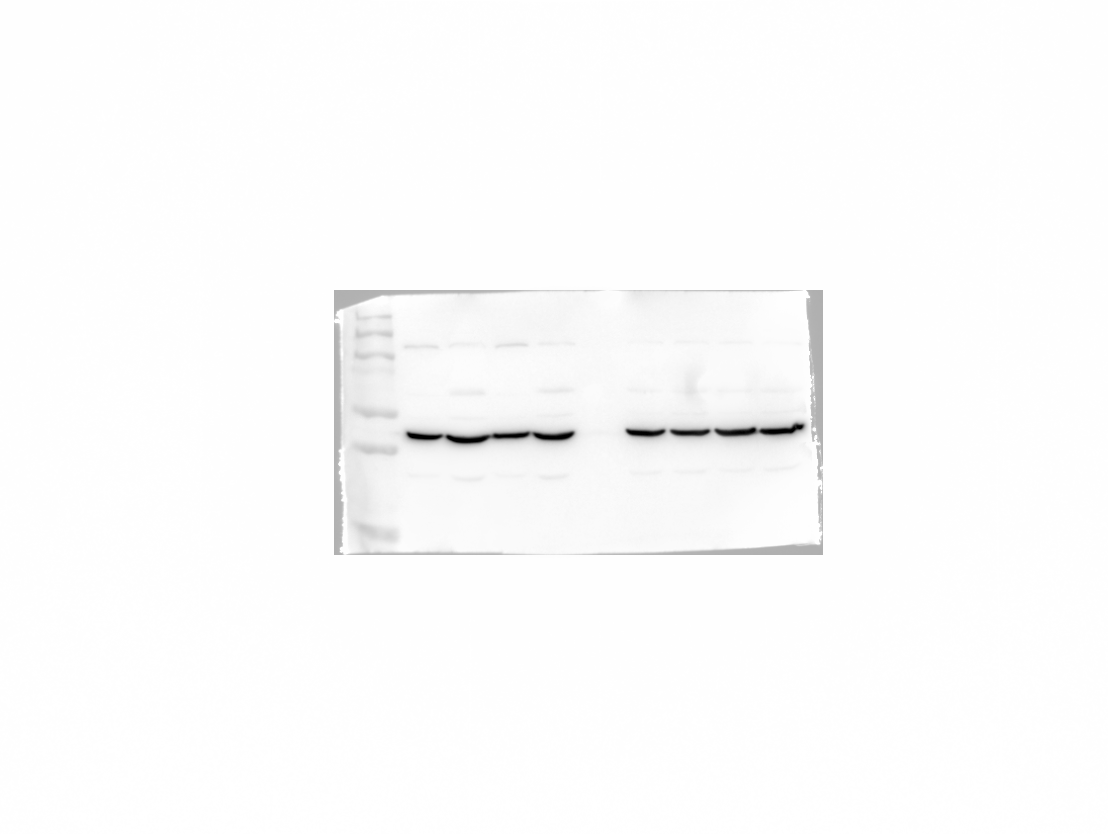

Supplement: Figure 3—source data 2. [file elife-96445-fig3-data2.zip › Figure 3_Source Data 2/Fig.3D actin.tif]

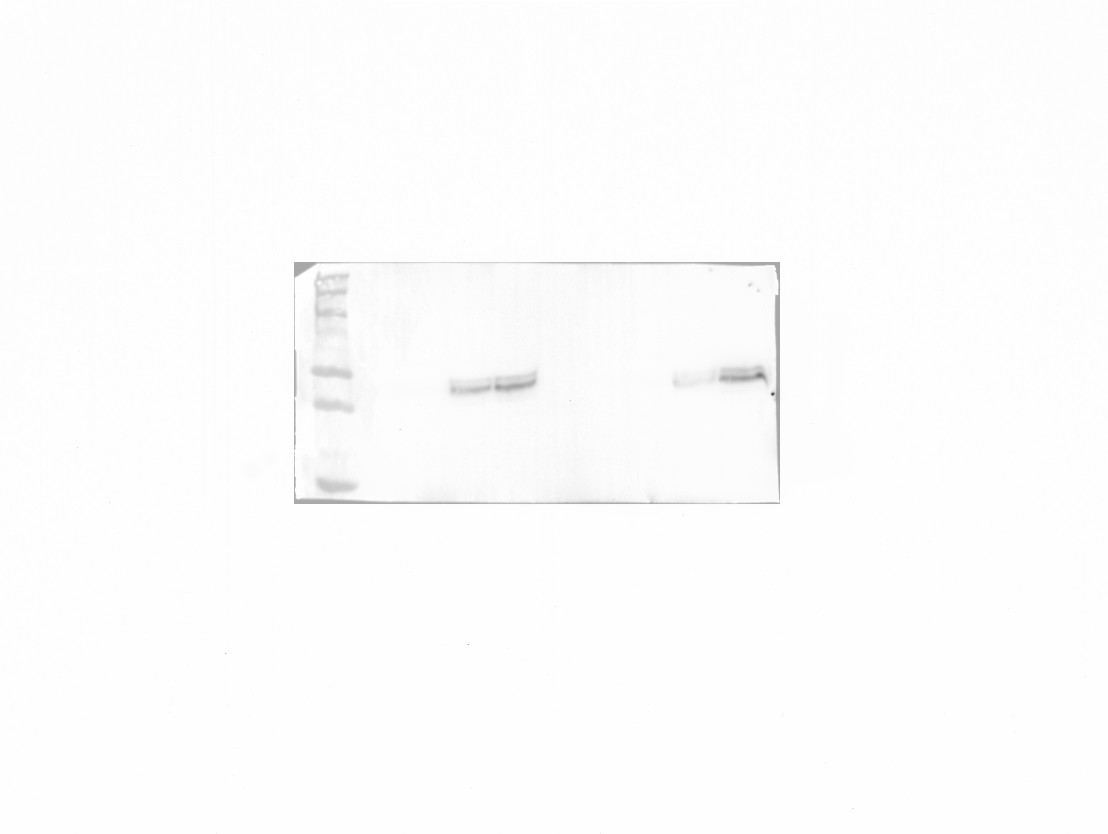

Supplement: Figure 3—source data 2. [file elife-96445-fig3-data2.zip › Figure 3_Source Data 2/Fig.3D PDL1.tif]

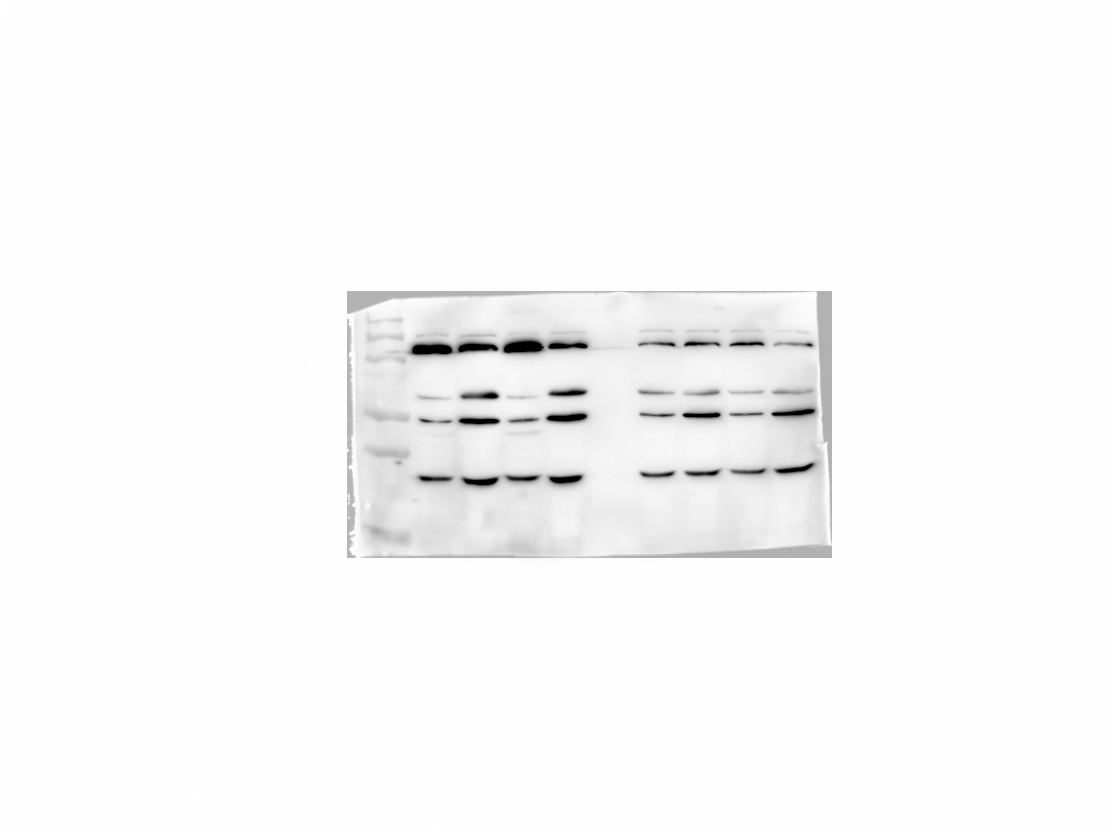

Supplement: Figure 3—source data 2. [file elife-96445-fig3-data2.zip › Figure 3_Source Data 2/Fig.3D ZFP36L1.tif]

**Fig.4D**

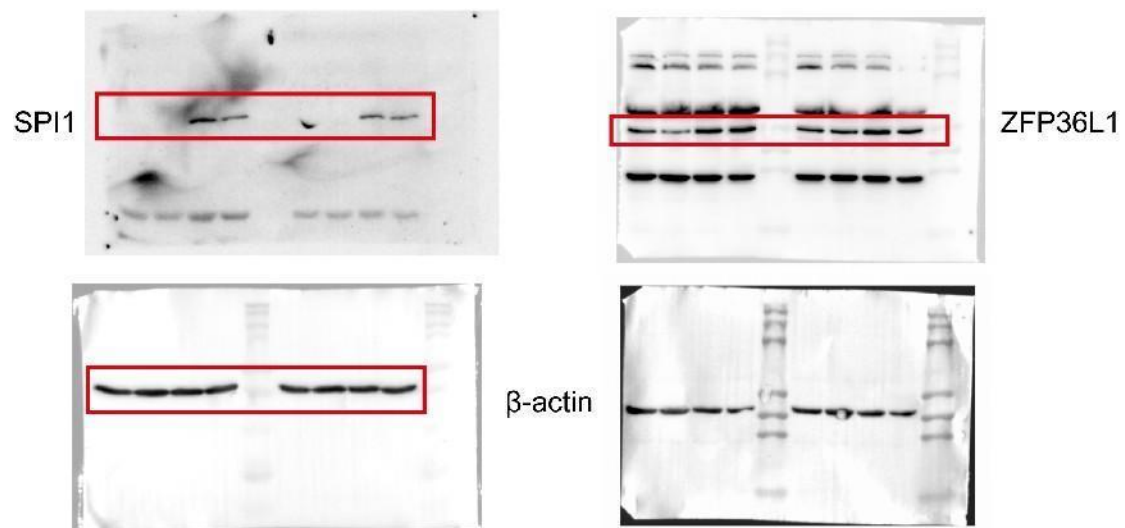

**Fig.4H**

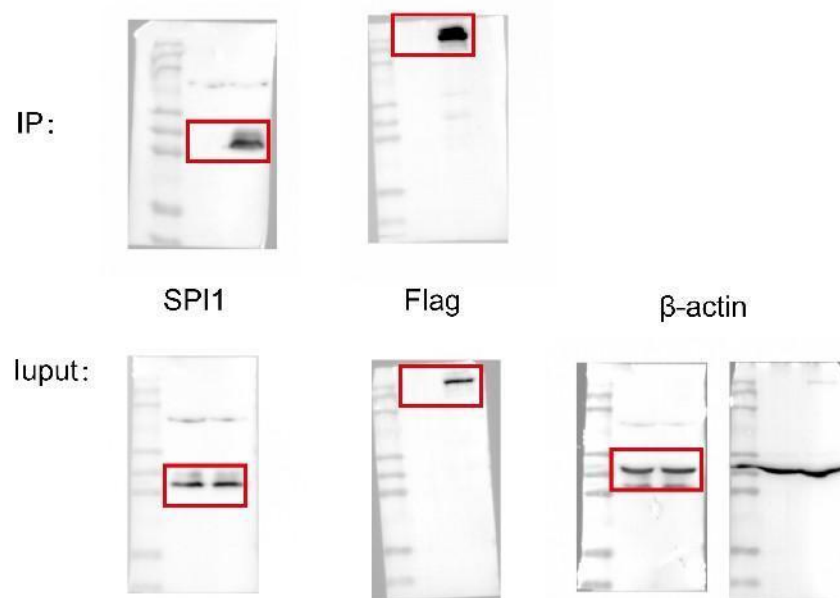

**Fig.4F**

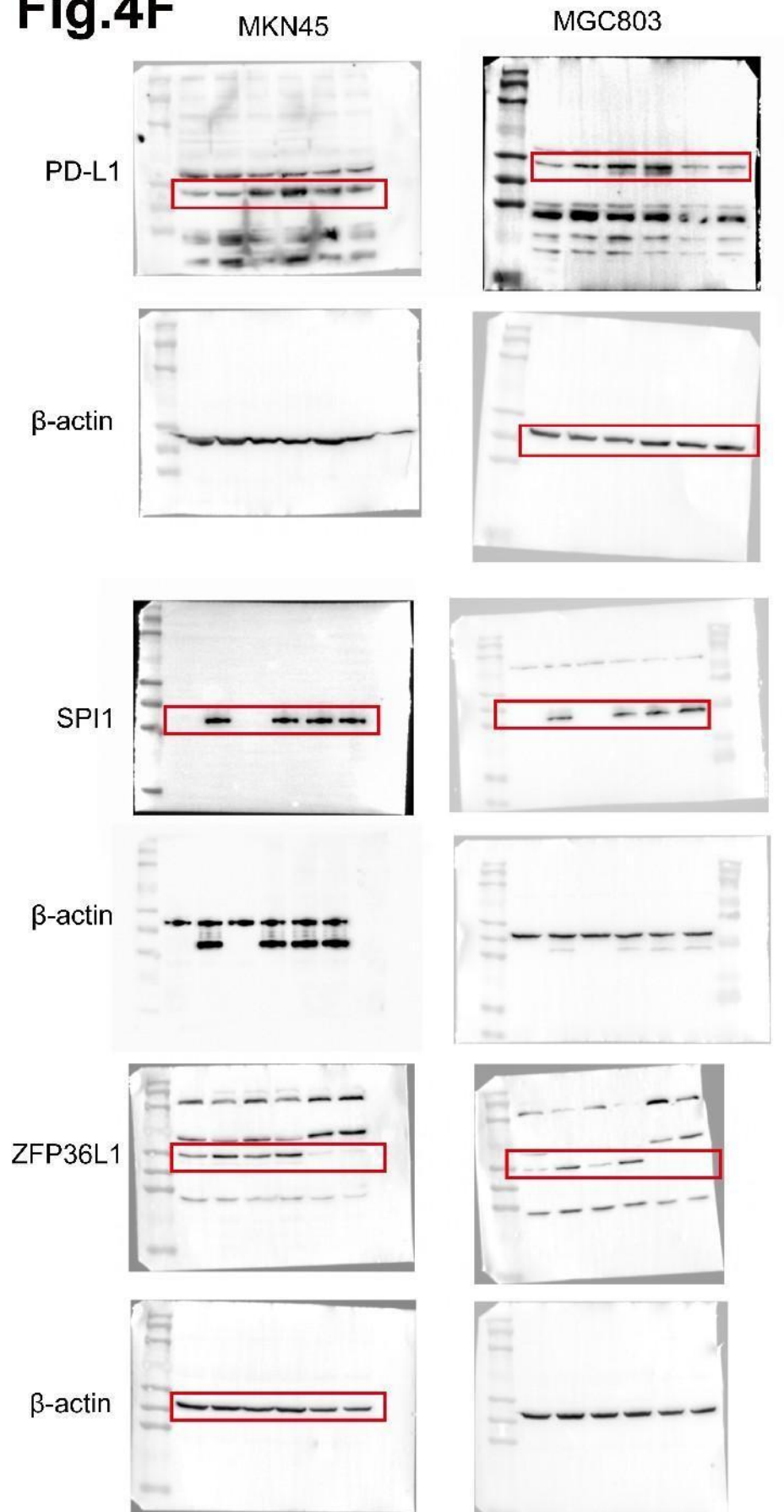

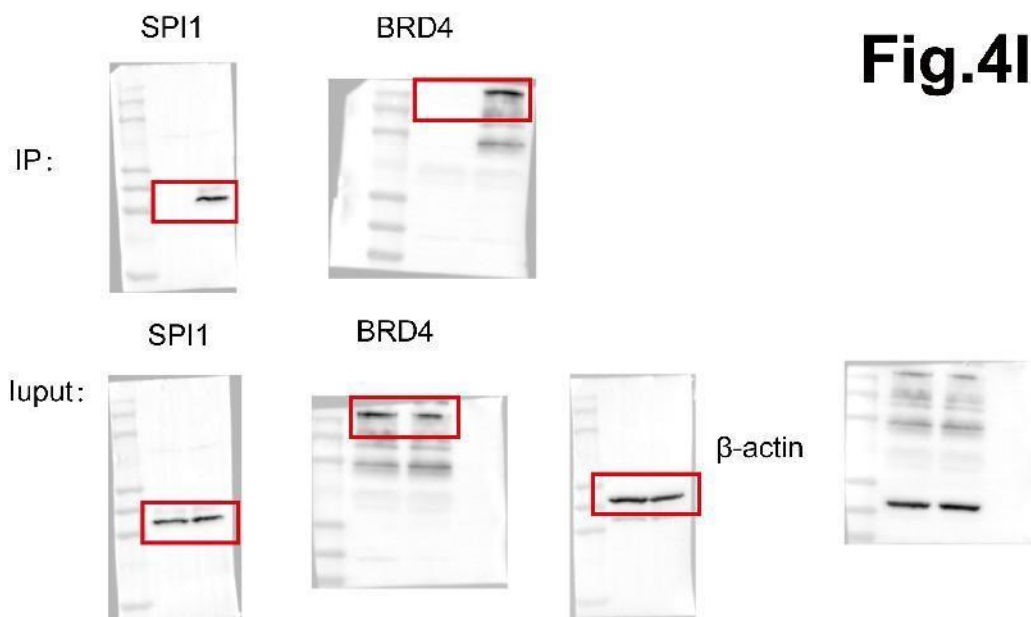

**Fig.4J**

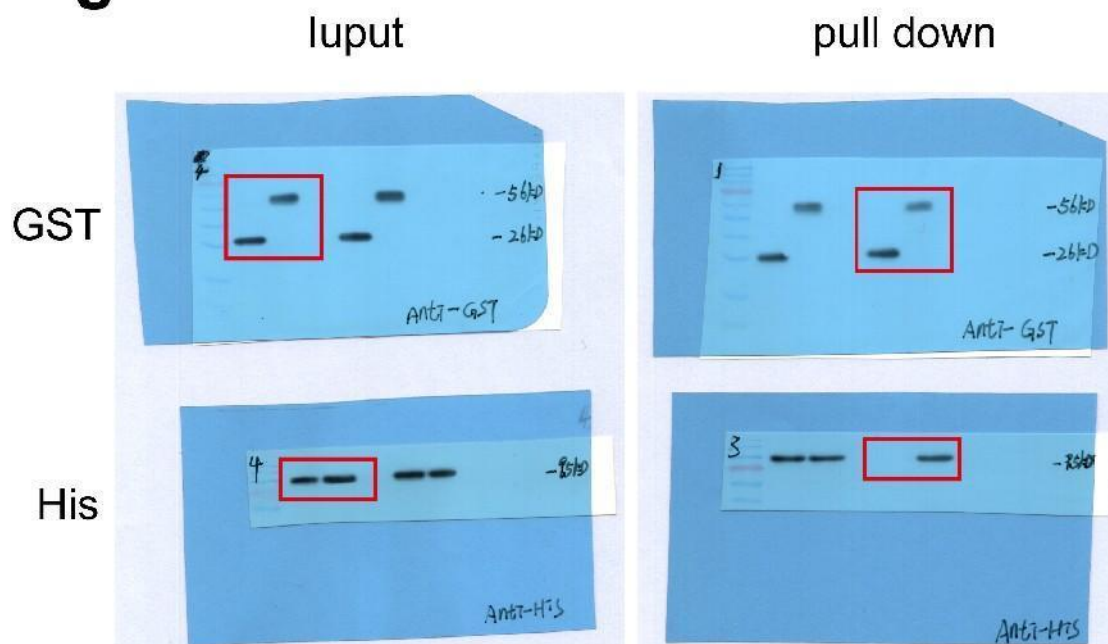

Figure 4, Source Data 1. Original blots corresponding to Figure 4

Supplement: Figure 4—source data 1. [file elife-96445-fig4-data1.pdf]

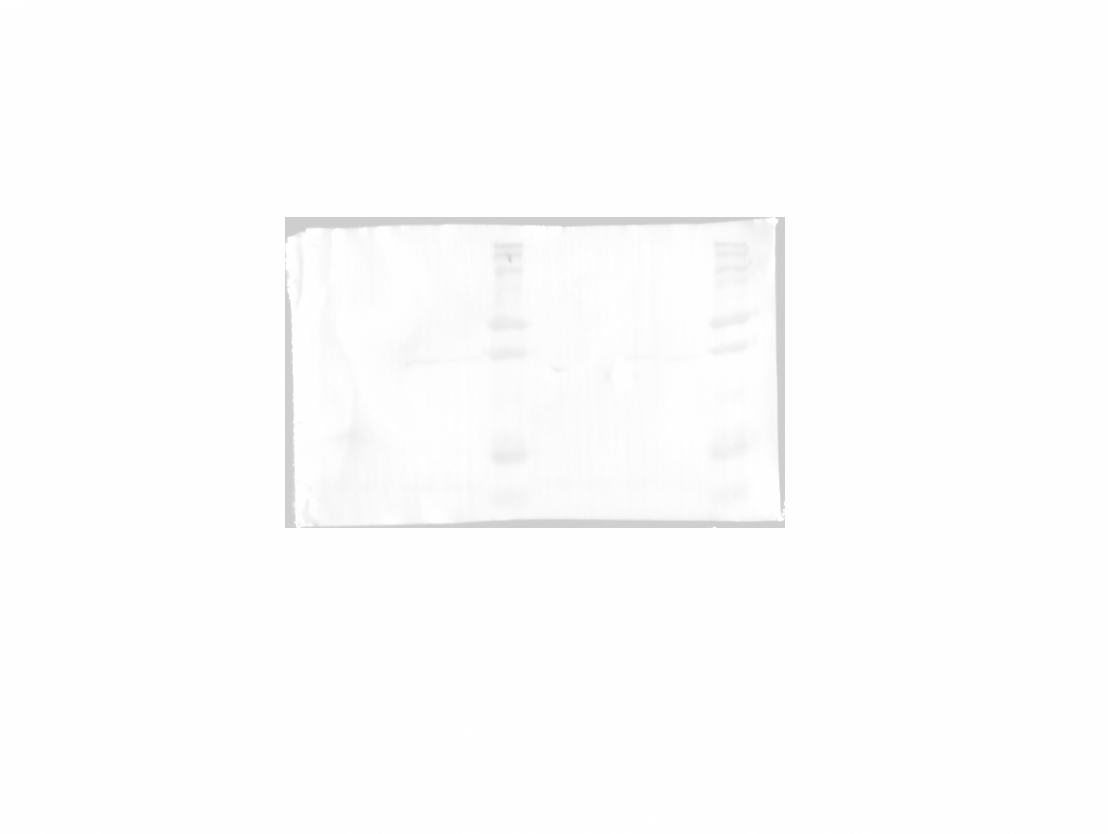

Supplement: Figure 4—source data 2. [file elife-96445-fig4-data2.zip › Figure 4_Source Data 2/4D/SPI1.tif]

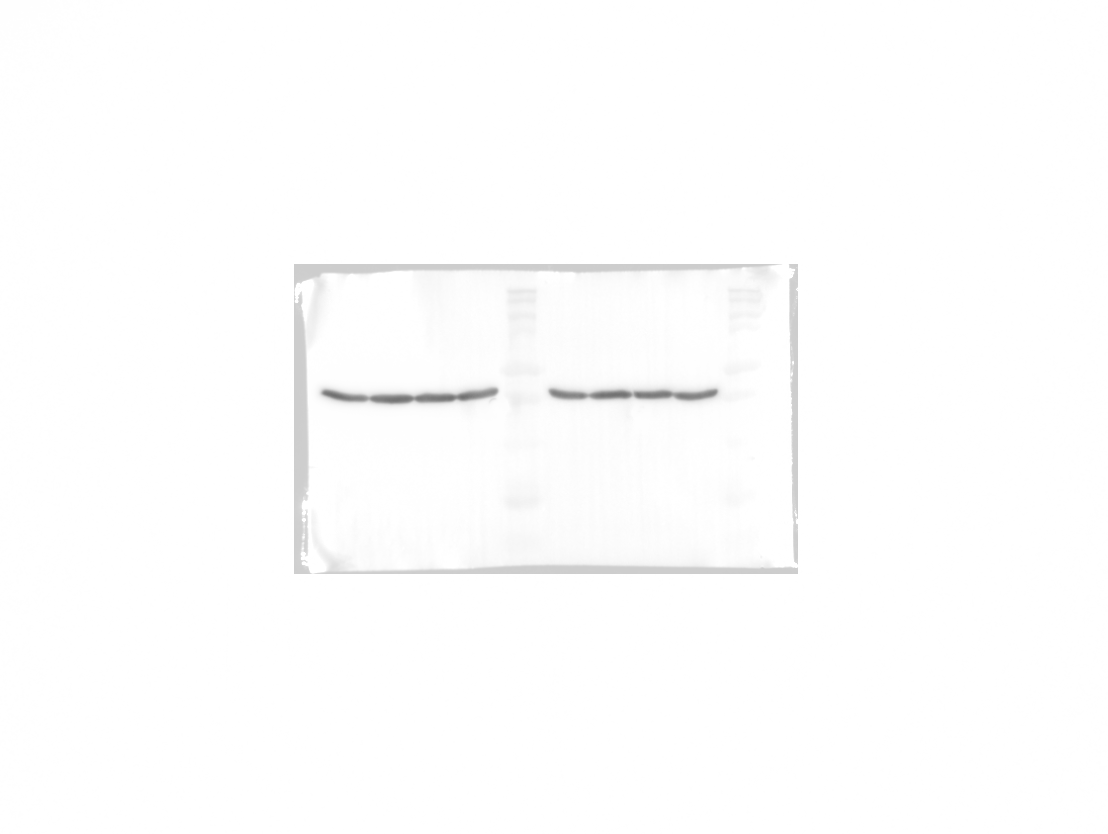

Supplement: Figure 4—source data 2. [file elife-96445-fig4-data2.zip › Figure 4_Source Data 2/4D/spi1actin.tif]

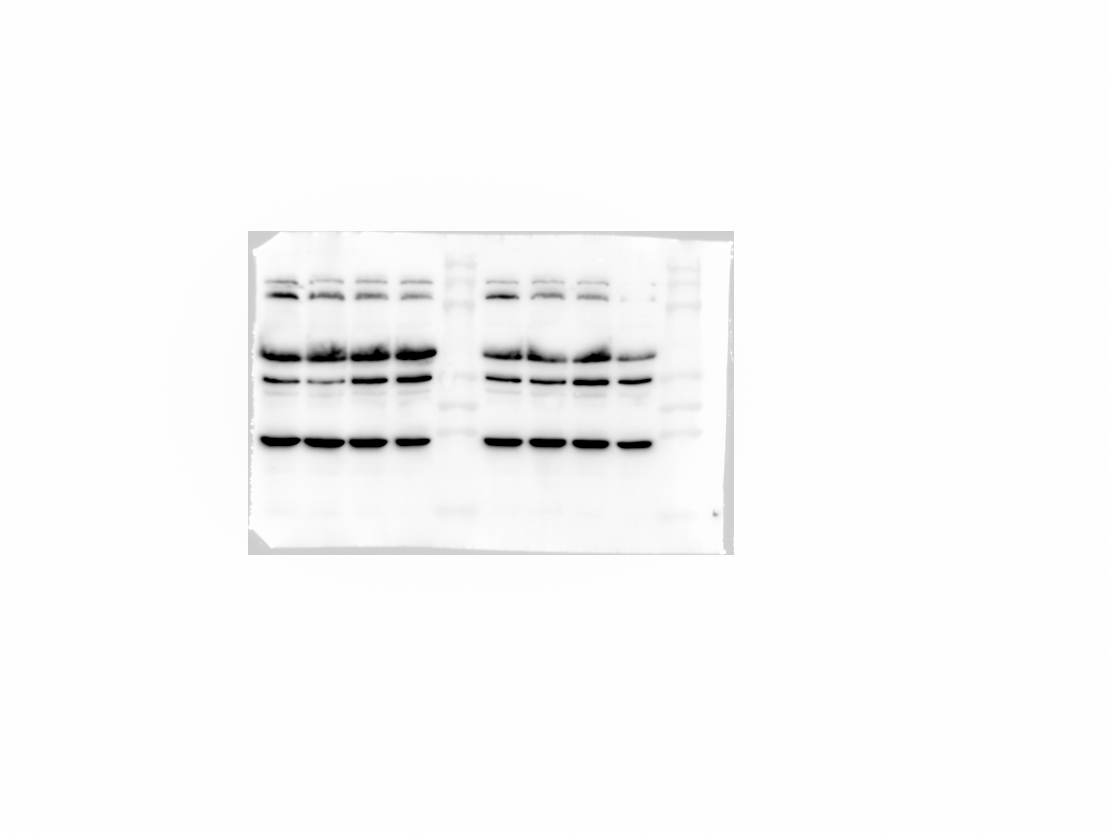

Supplement: Figure 4—source data 2. [file elife-96445-fig4-data2.zip › Figure 4_Source Data 2/4D/ZFP36L11.tif]

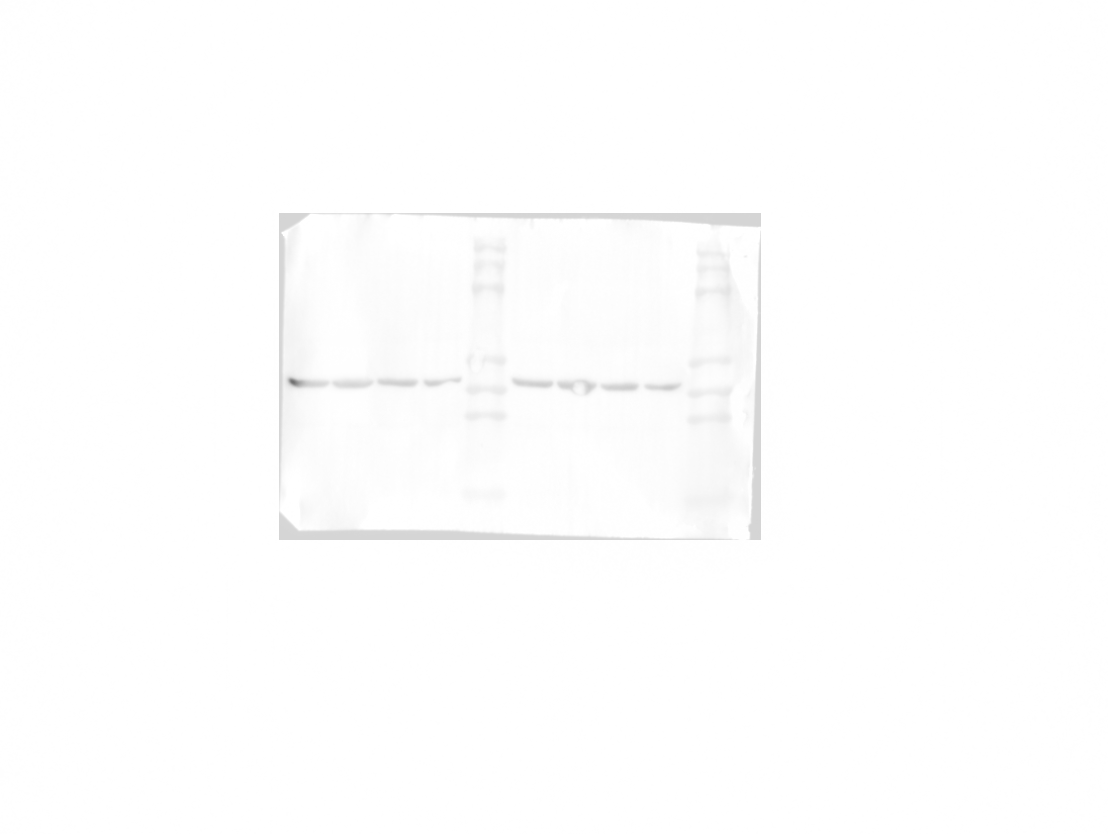

Supplement: Figure 4—source data 2. [file elife-96445-fig4-data2.zip › Figure 4_Source Data 2/4D/ZFPactin.tif]

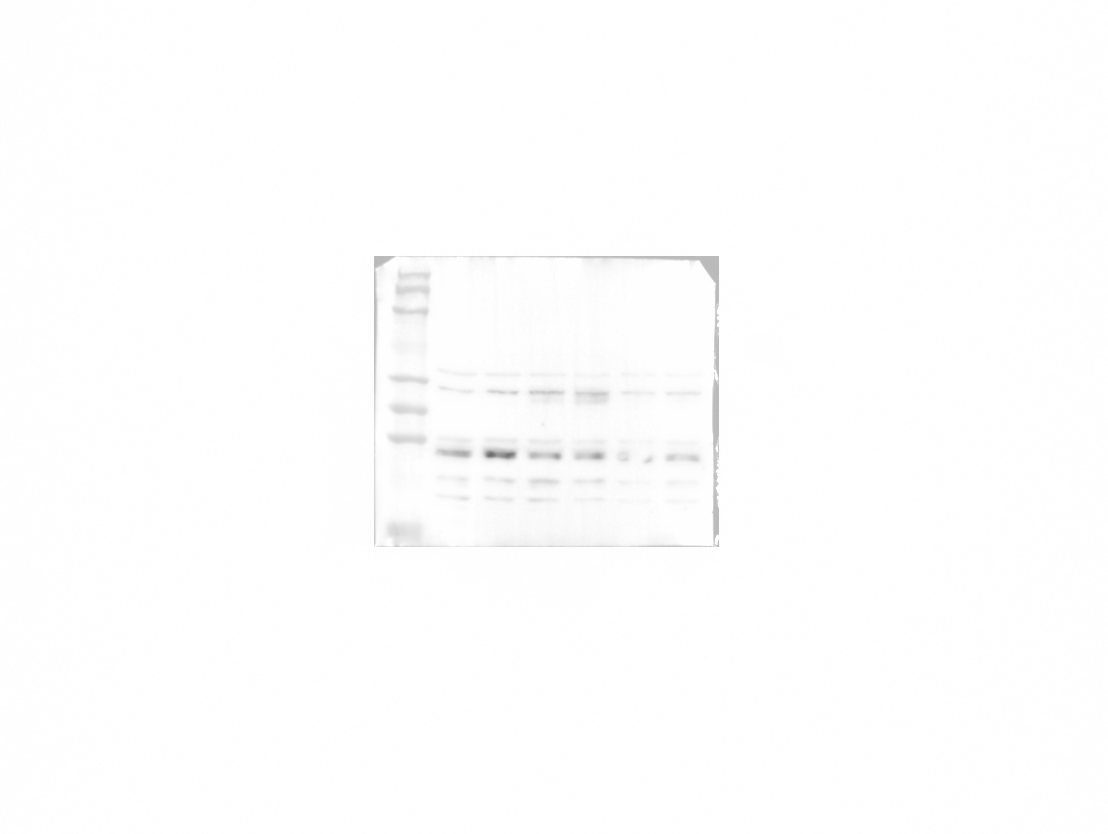

Supplement: Figure 4—source data 2. [file elife-96445-fig4-data2.zip › Figure 4_Source Data 2/4F-MGC803/pd-l1.tif]

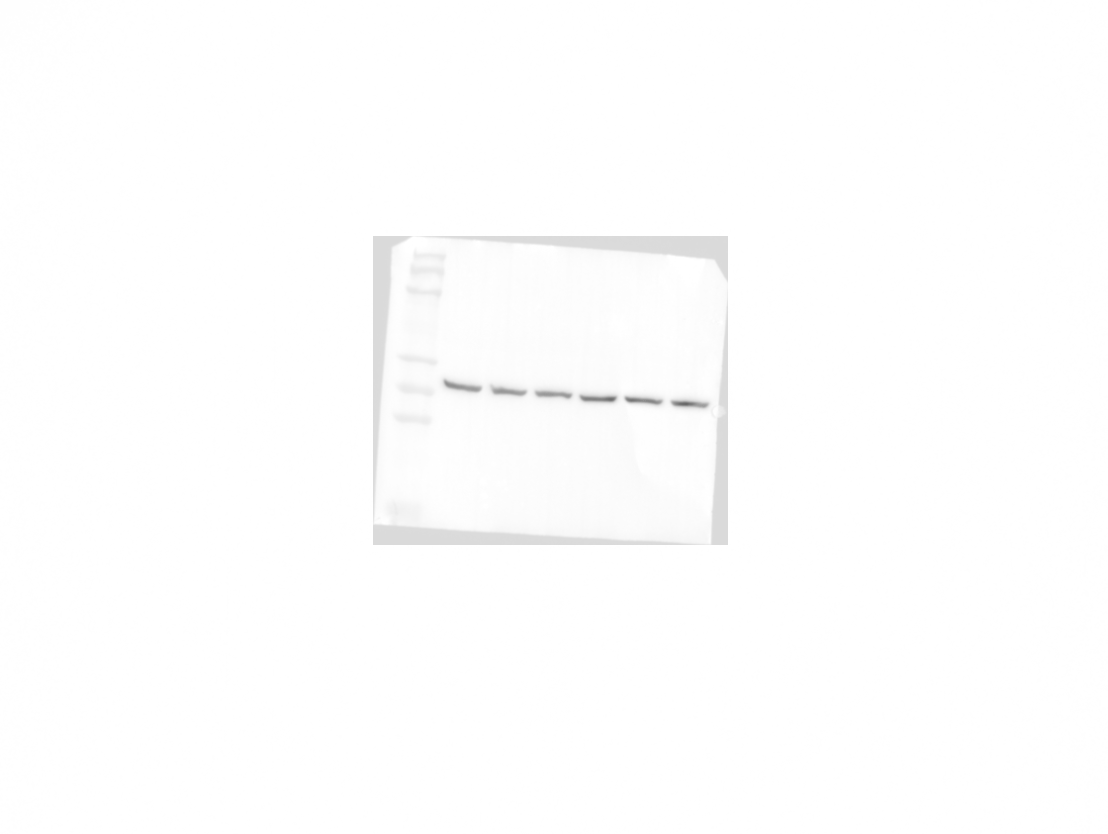

Supplement: Figure 4—source data 2. [file elife-96445-fig4-data2.zip › Figure 4_Source Data 2/4F-MGC803/pdl1actin.tif]

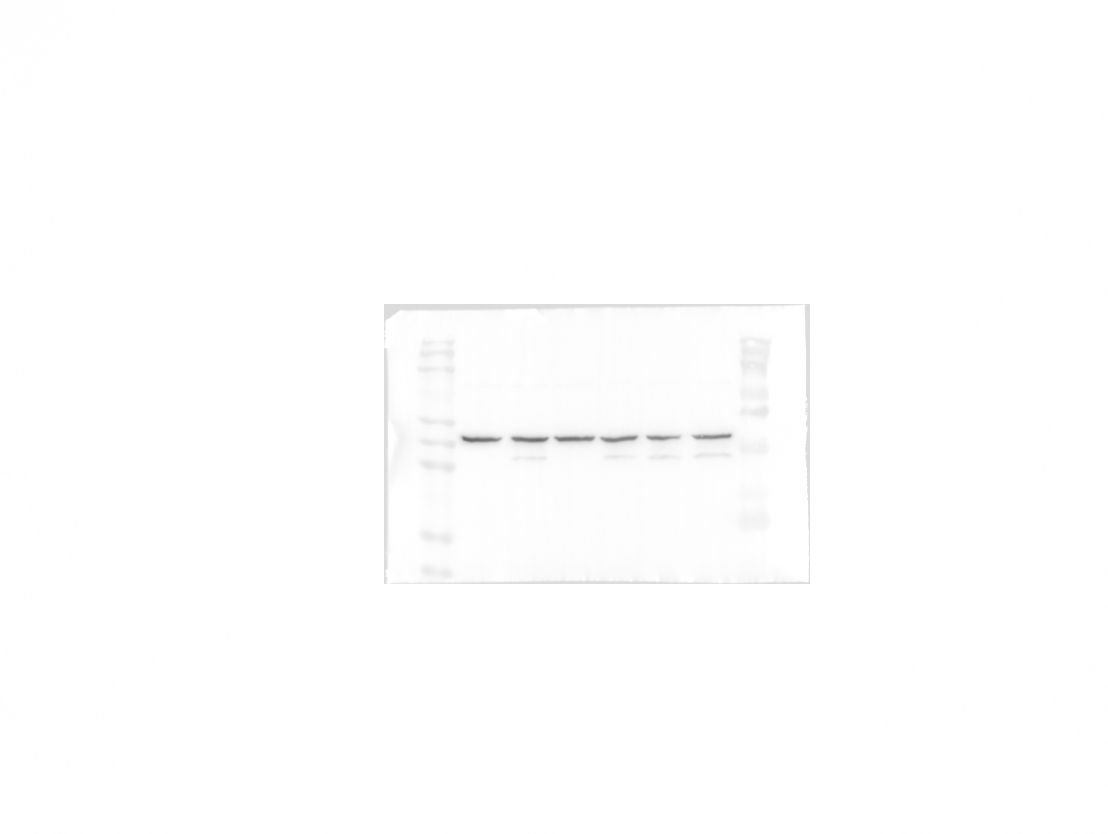

Supplement: Figure 4—source data 2. [file elife-96445-fig4-data2.zip › Figure 4_Source Data 2/4F-MGC803/SPI1-actin.tif]

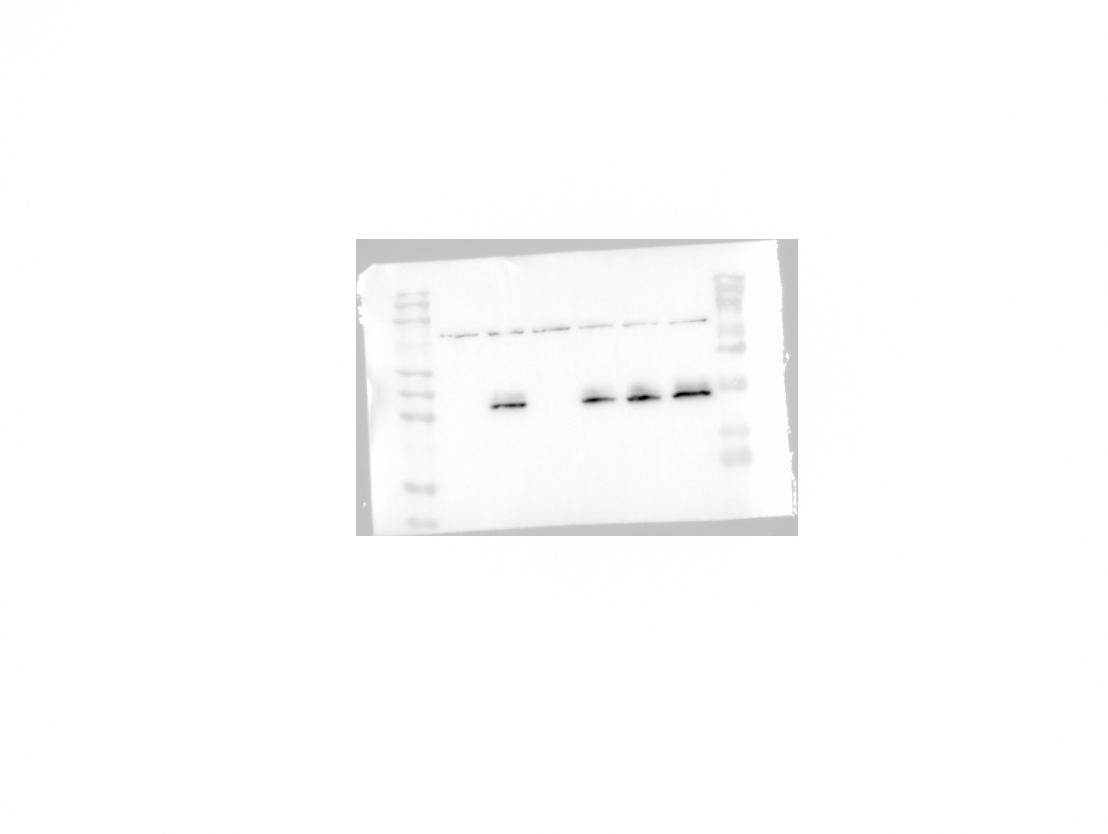

Supplement: Figure 4—source data 2. [file elife-96445-fig4-data2.zip › Figure 4_Source Data 2/4F-MGC803/SPI1.tif]

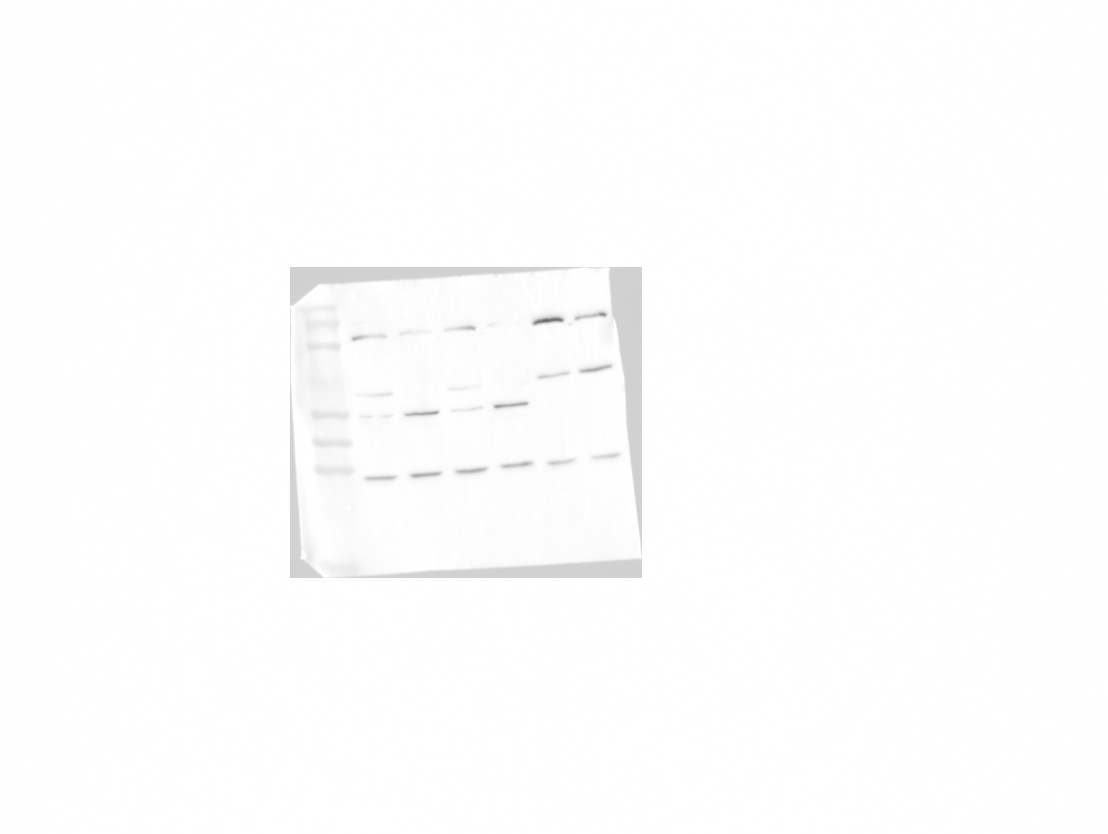

Supplement: Figure 4—source data 2. [file elife-96445-fig4-data2.zip › Figure 4_Source Data 2/4F-MGC803/zfp.tif]

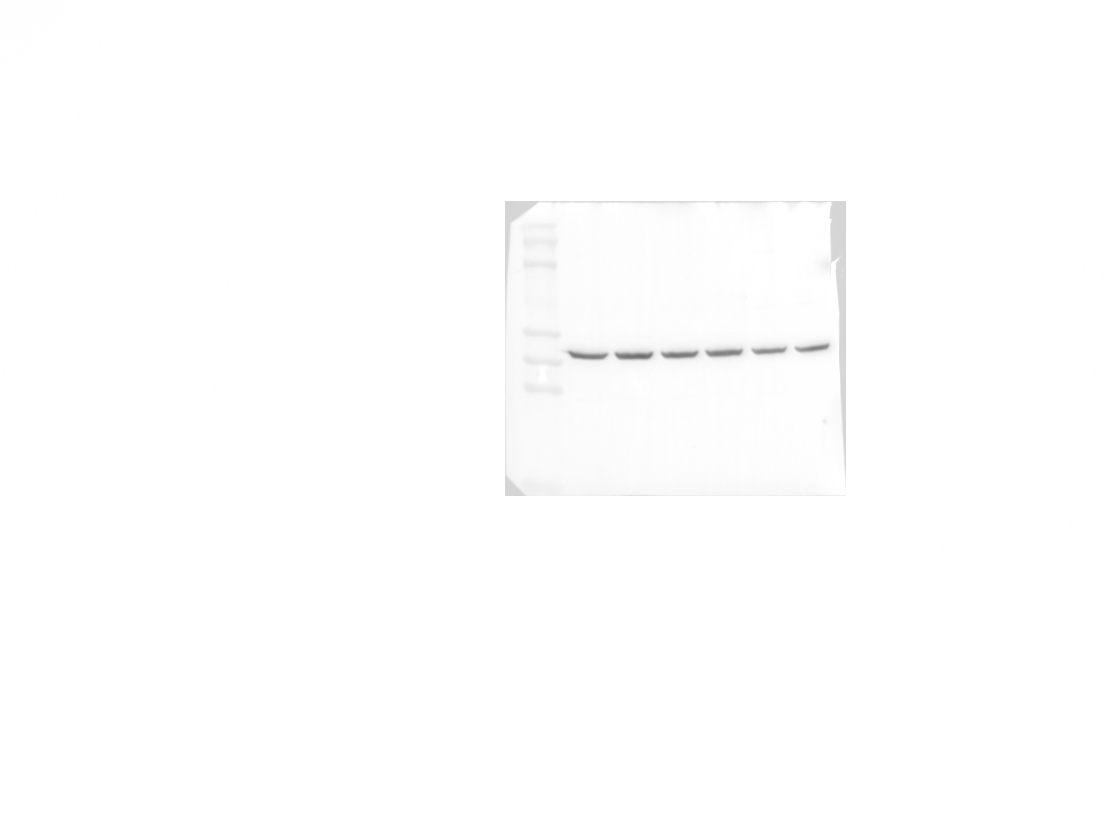

Supplement: Figure 4—source data 2. [file elife-96445-fig4-data2.zip › Figure 4_Source Data 2/4F-MGC803/zfpactin.tif]

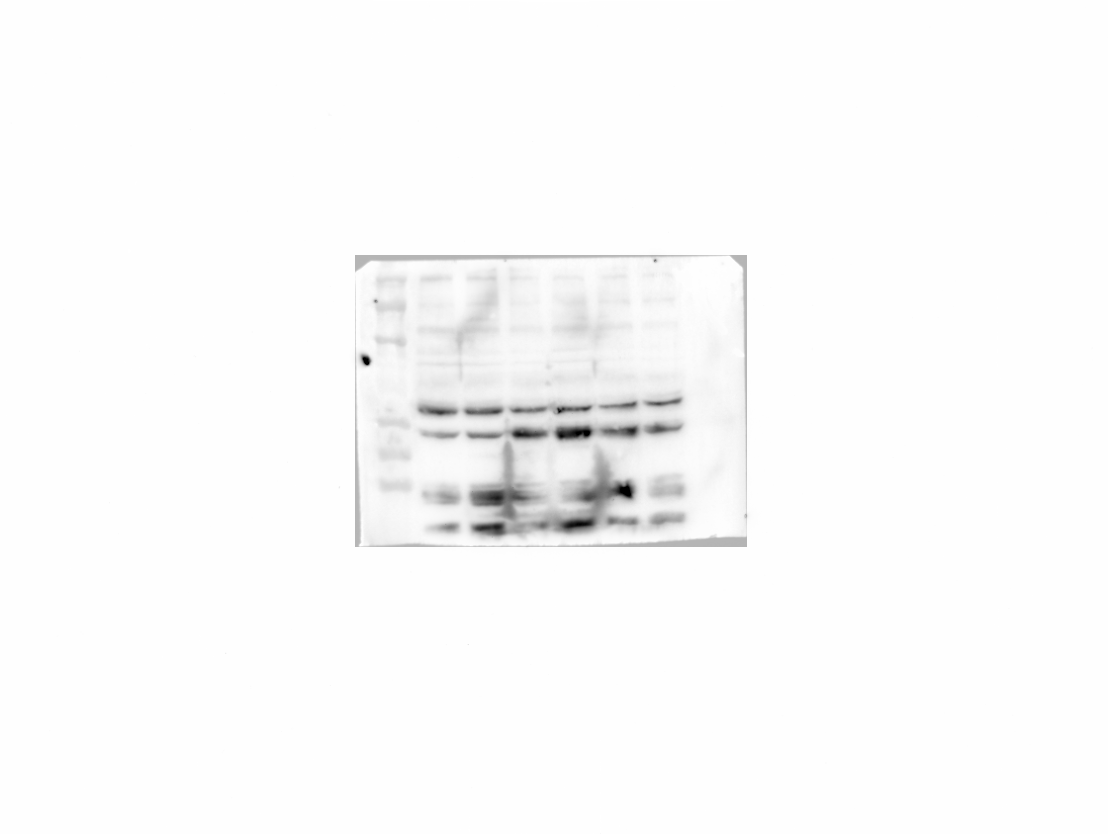

Supplement: Figure 4—source data 2. [file elife-96445-fig4-data2.zip › Figure 4_Source Data 2/4F-MKN45/pd-l1.tif]

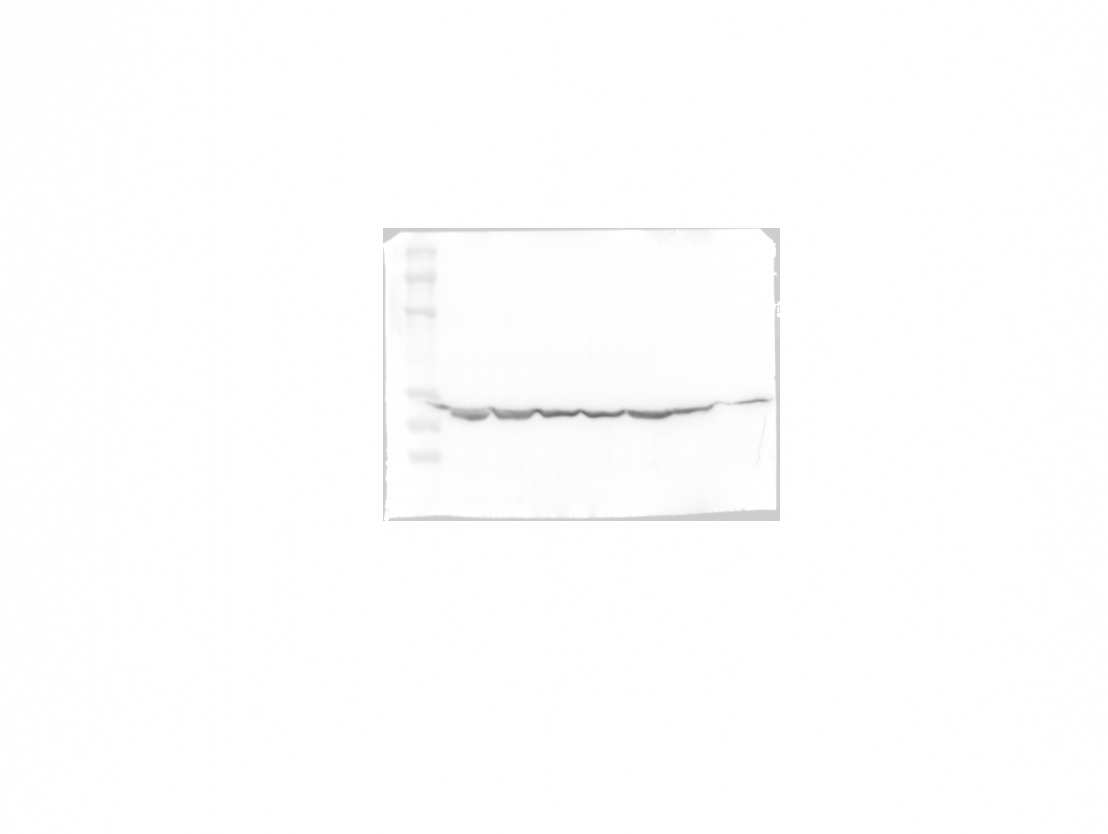

Supplement: Figure 4—source data 2. [file elife-96445-fig4-data2.zip › Figure 4_Source Data 2/4F-MKN45/PDL1actin.tif]

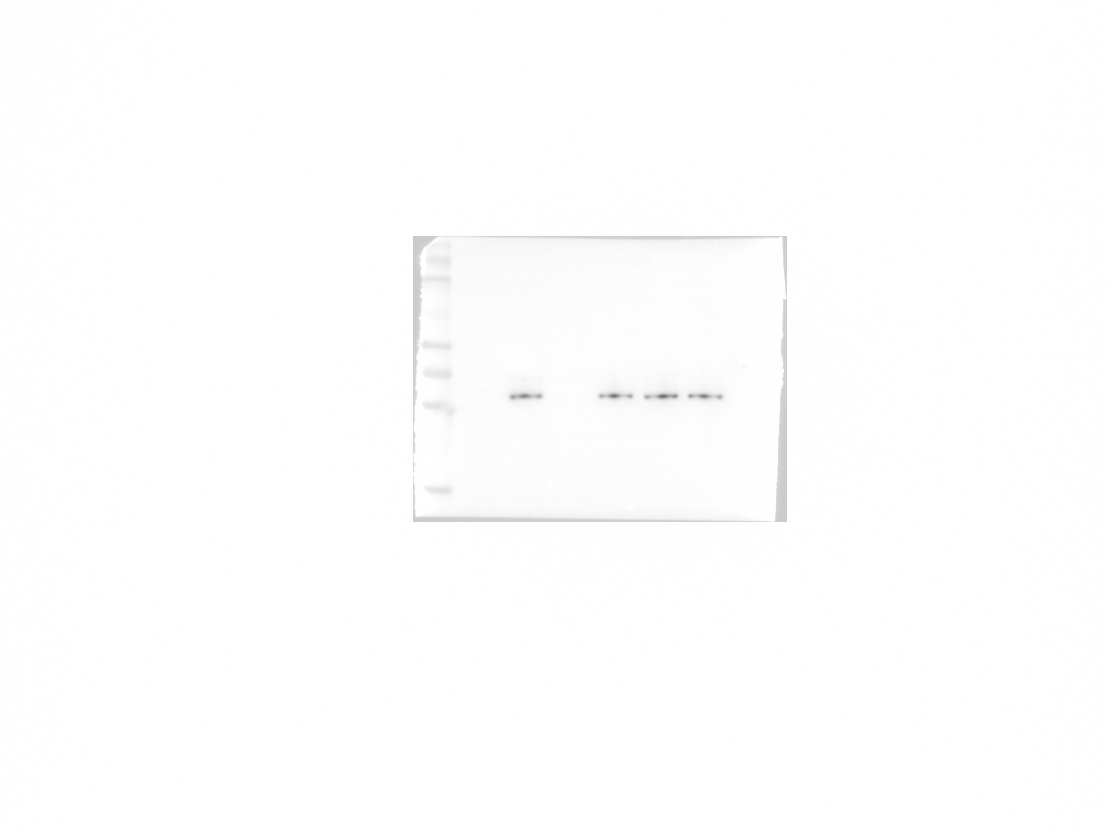

Supplement: Figure 4—source data 2. [file elife-96445-fig4-data2.zip › Figure 4_Source Data 2/4F-MKN45/spi1.tif]

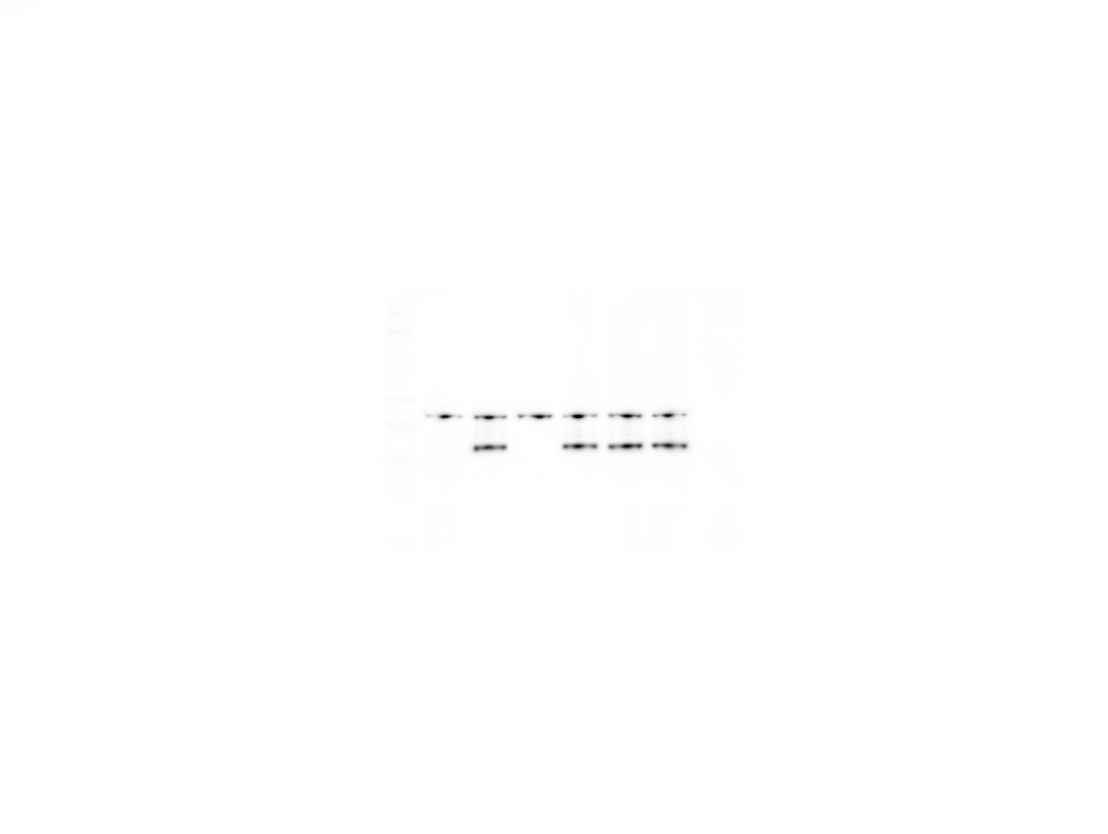

Supplement: Figure 4—source data 2. [file elife-96445-fig4-data2.zip › Figure 4_Source Data 2/4F-MKN45/spi1actin.tif]

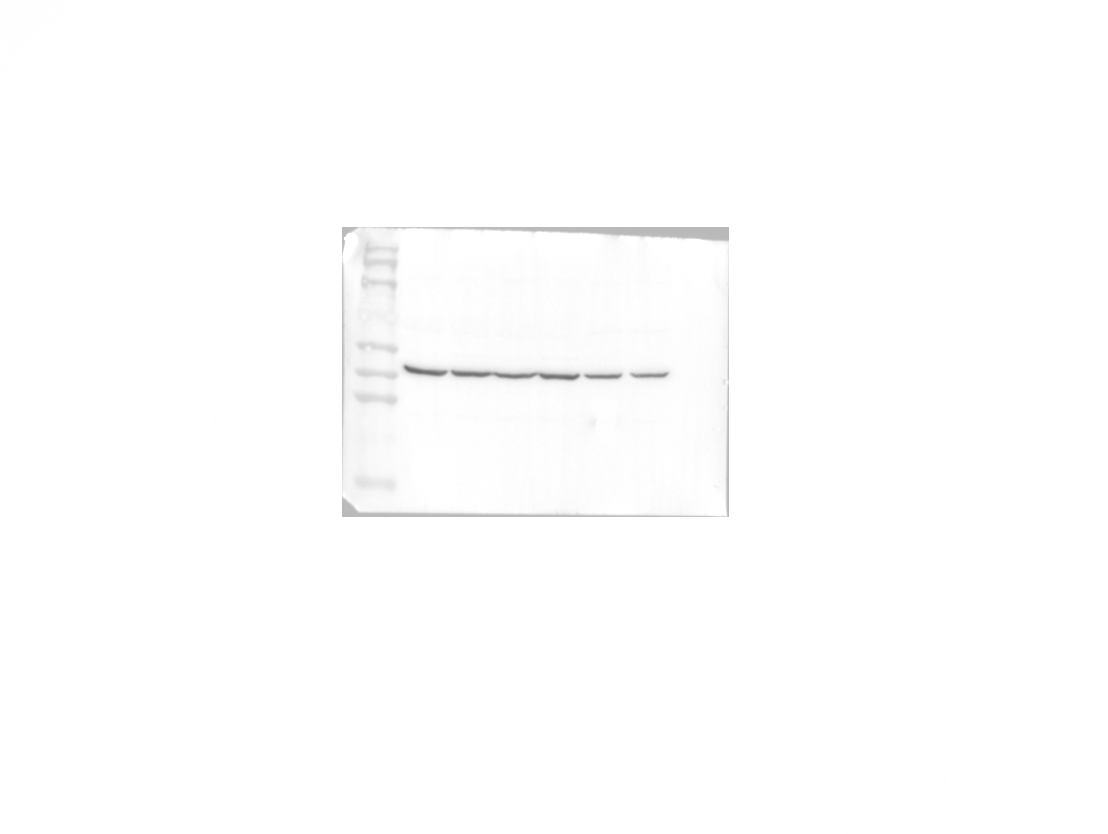

Supplement: Figure 4—source data 2. [file elife-96445-fig4-data2.zip › Figure 4_Source Data 2/4F-MKN45/zfp-actin.tif]

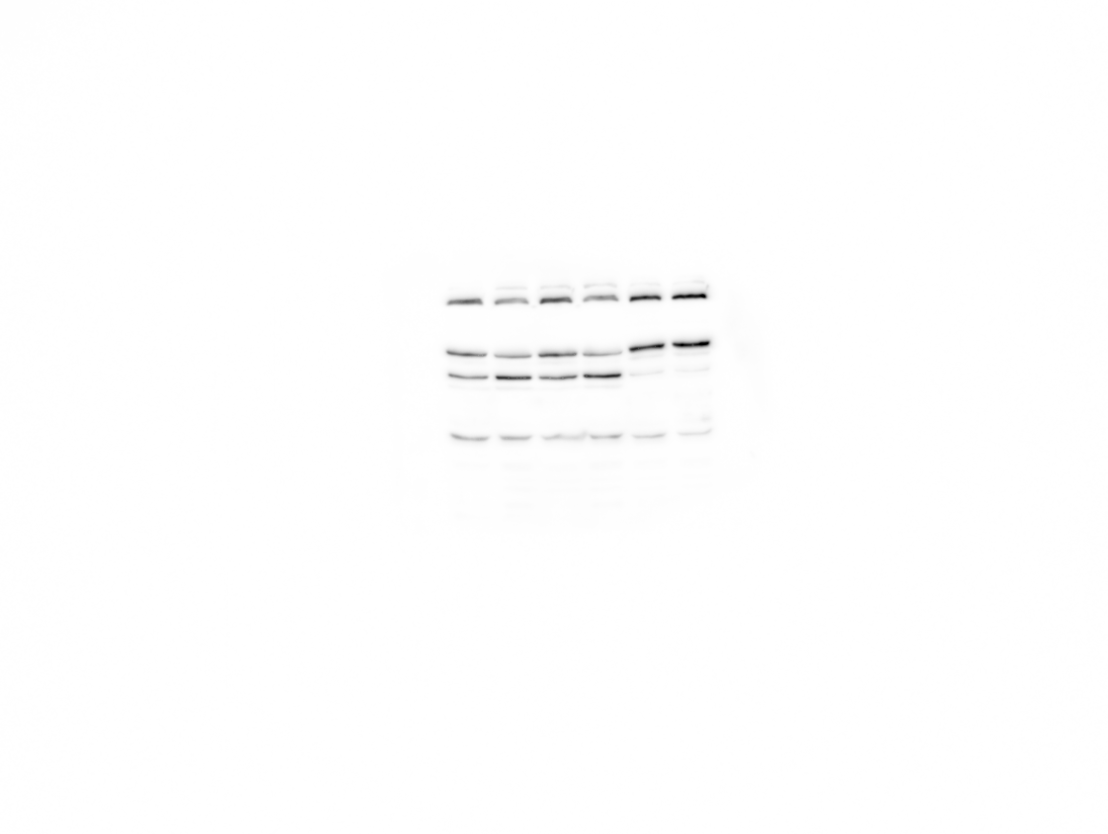

Supplement: Figure 4—source data 2. [file elife-96445-fig4-data2.zip › Figure 4_Source Data 2/4F-MKN45/ZFP.tif]

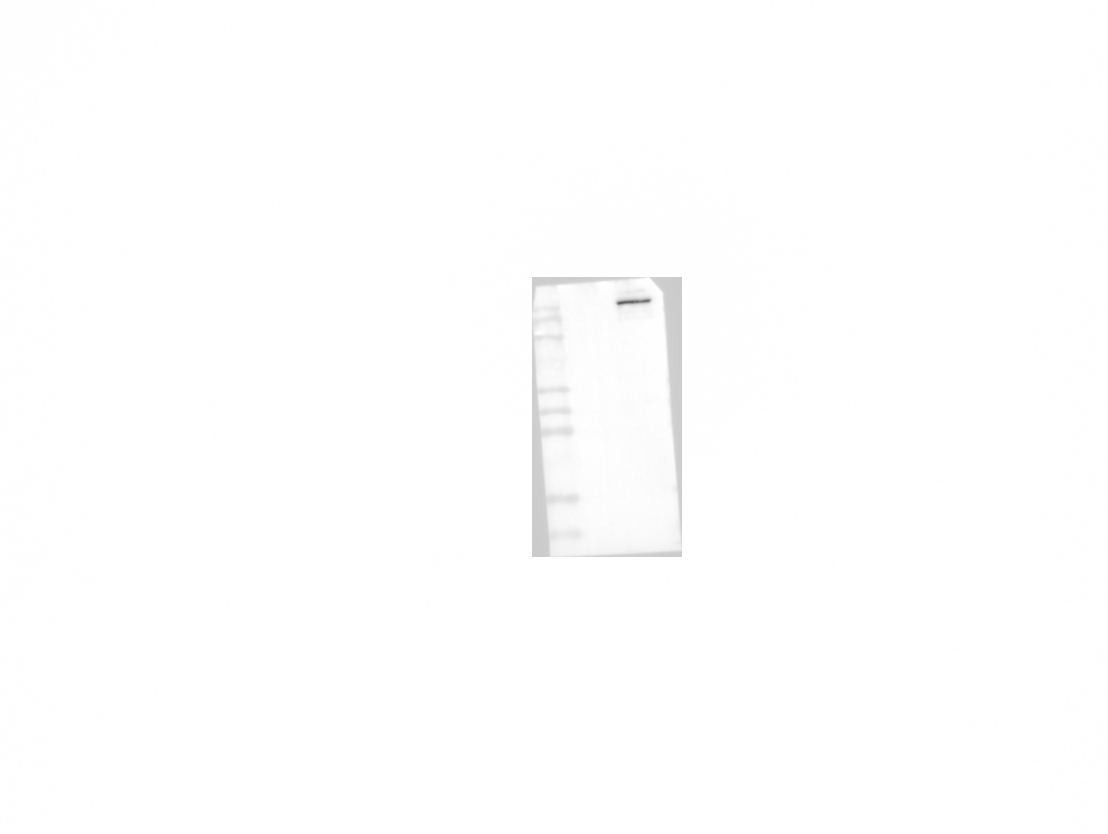

Supplement: Figure 4—source data 2. [file elife-96445-fig4-data2.zip › Figure 4_Source Data 2/4H/brd4-input.tif]

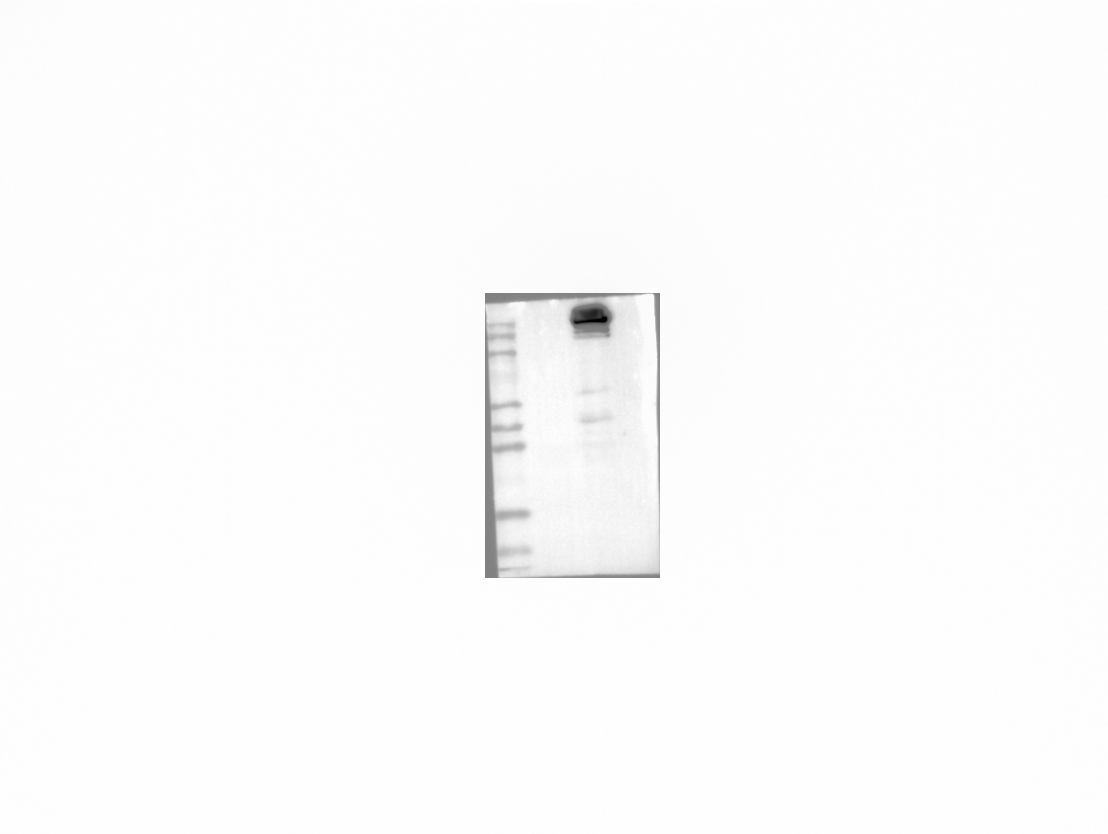

Supplement: Figure 4—source data 2. [file elife-96445-fig4-data2.zip › Figure 4_Source Data 2/4H/brd4-ip222.tif]

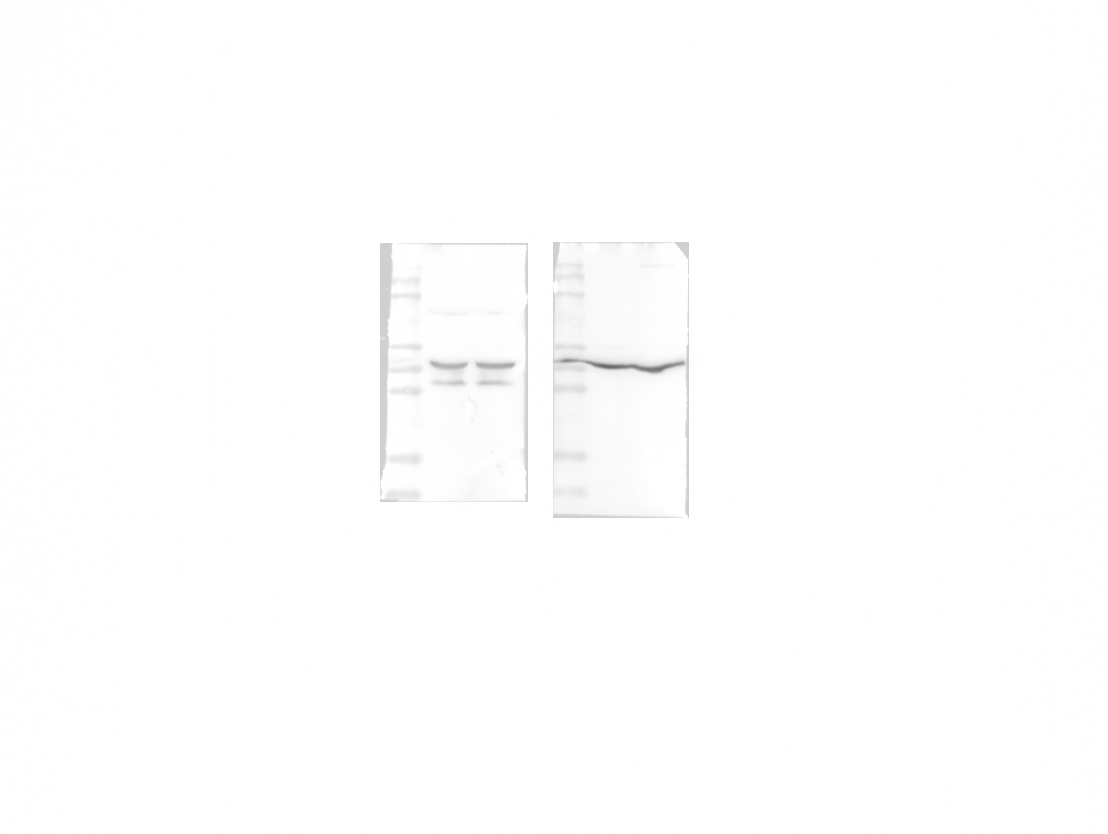

Supplement: Figure 4—source data 2. [file elife-96445-fig4-data2.zip › Figure 4_Source Data 2/4H/input-actin.tif]

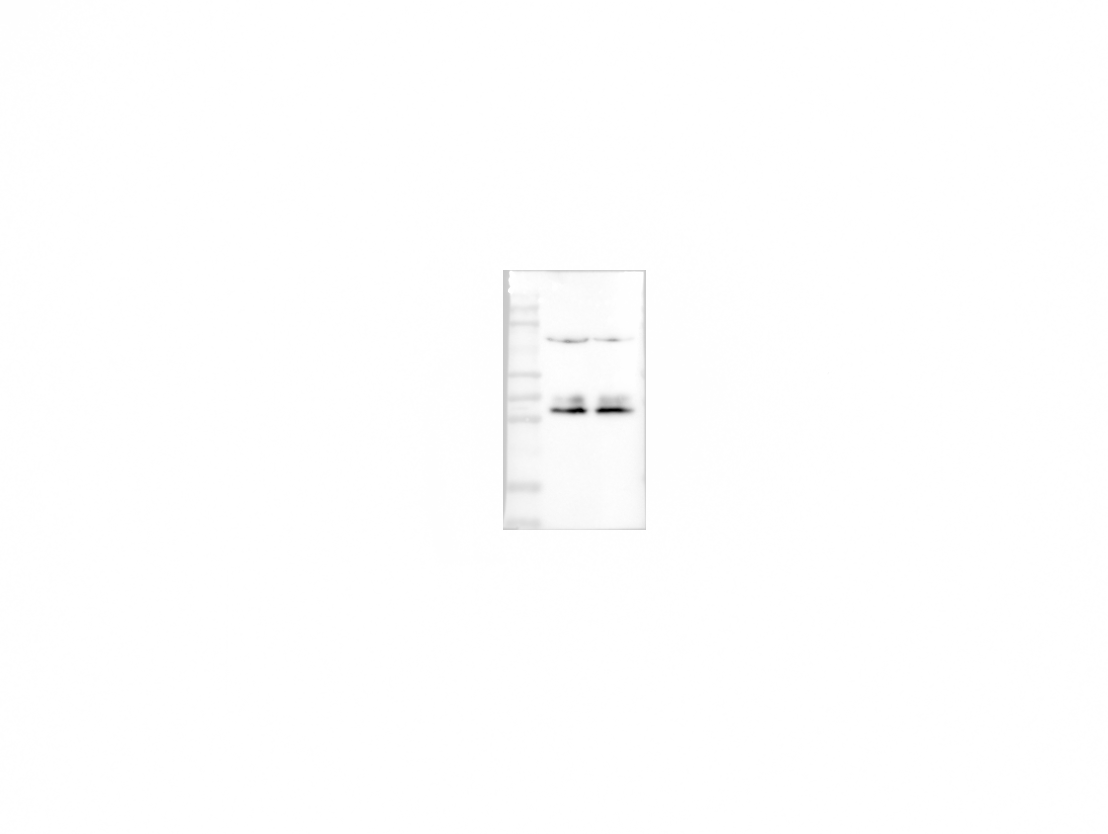

Supplement: Figure 4—source data 2. [file elife-96445-fig4-data2.zip › Figure 4_Source Data 2/4H/spi1-input.tif]

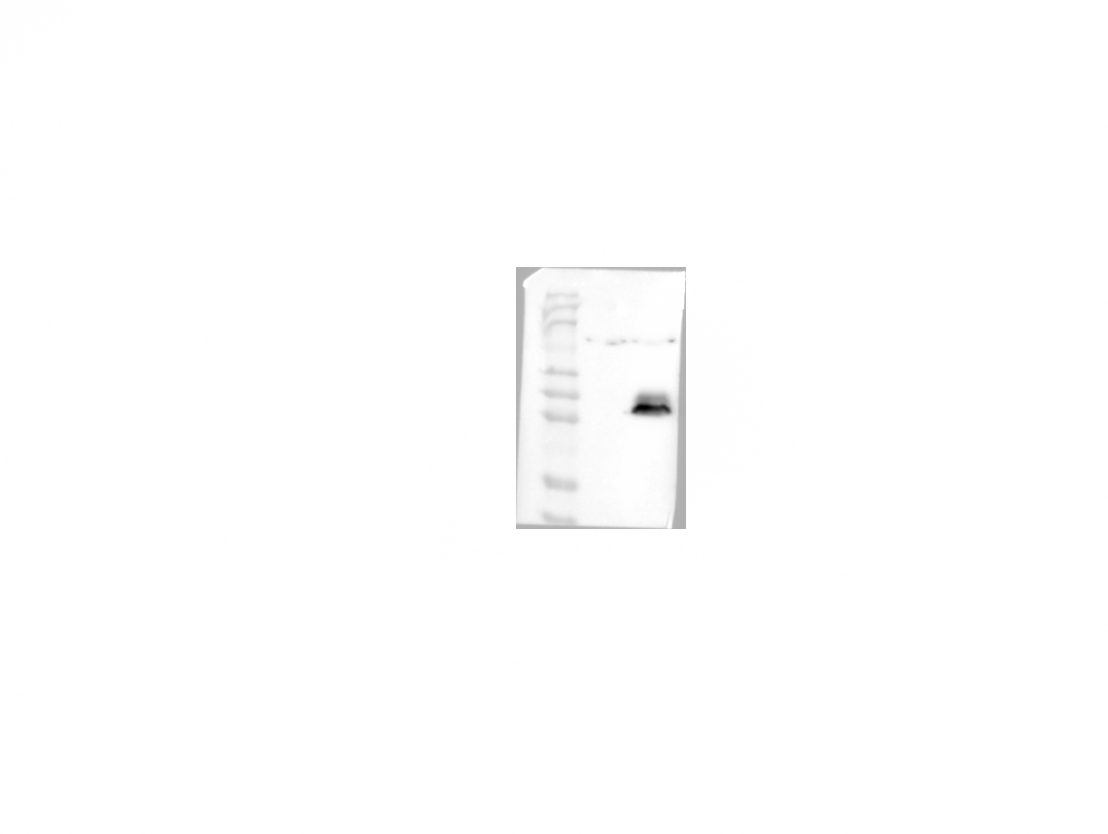

Supplement: Figure 4—source data 2. [file elife-96445-fig4-data2.zip › Figure 4_Source Data 2/4H/SPI1-ip.tif]

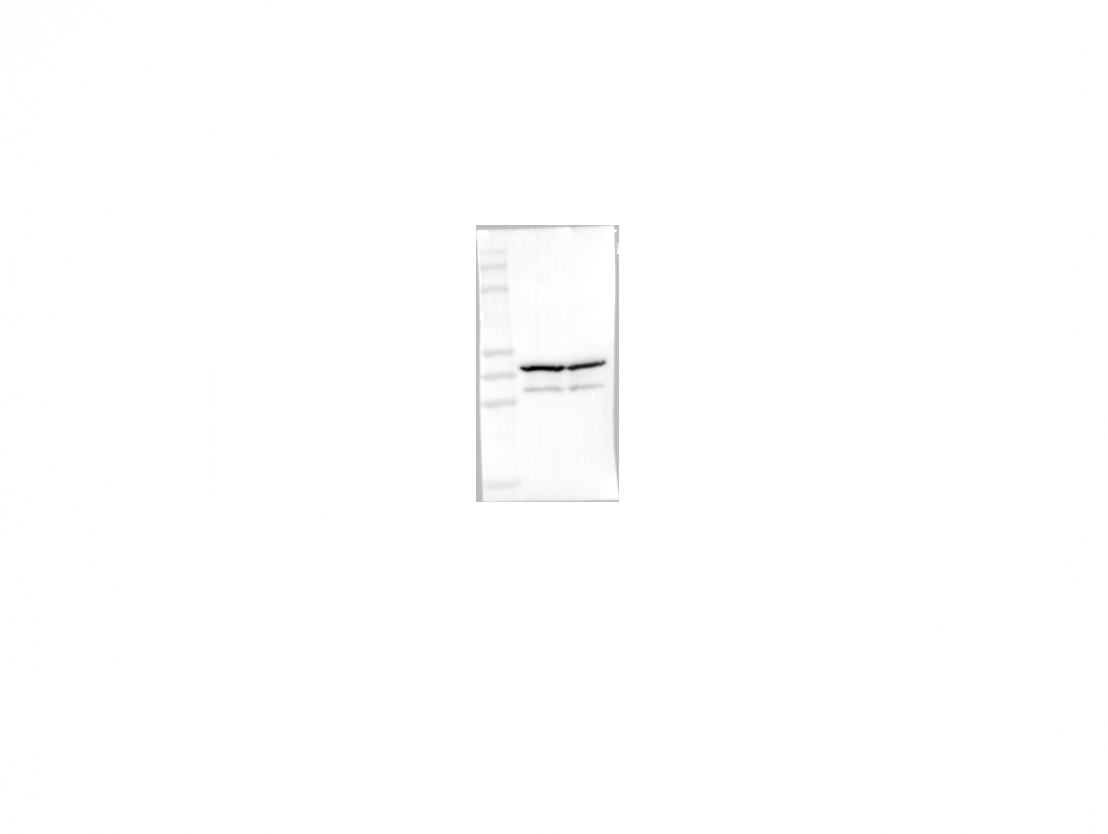

Supplement: Figure 4—source data 2. [file elife-96445-fig4-data2.zip › Figure 4_Source Data 2/4I/ACTIN-SPI1INPUT.tif]

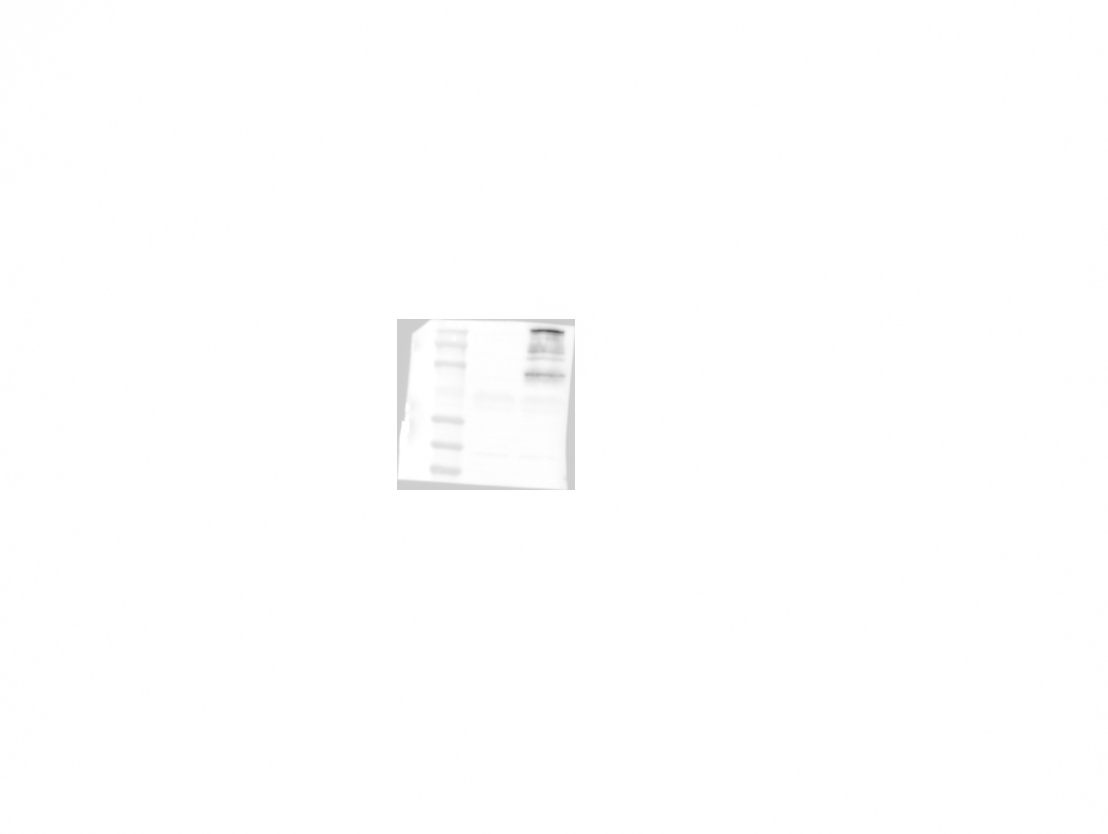

Supplement: Figure 4—source data 2. [file elife-96445-fig4-data2.zip › Figure 4_Source Data 2/4I/BRD4-IB.tif]

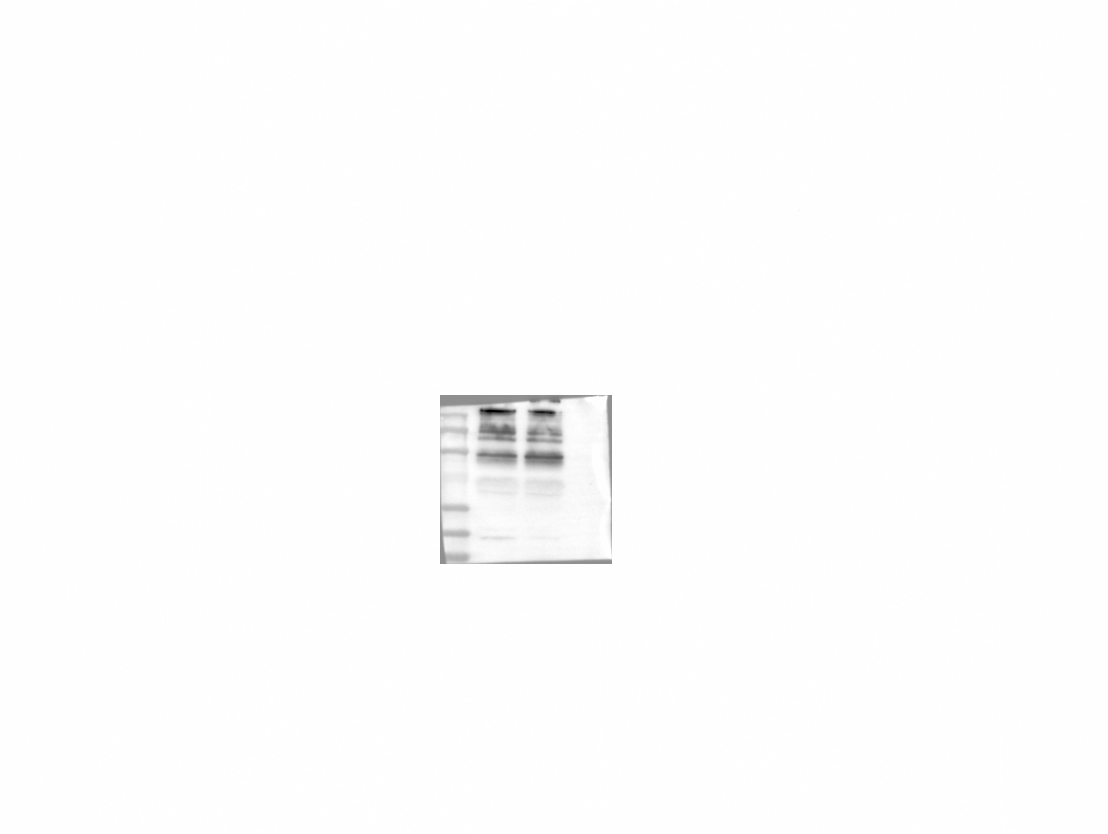

Supplement: Figure 4—source data 2. [file elife-96445-fig4-data2.zip › Figure 4_Source Data 2/4I/BRD4-INPUT.tif]

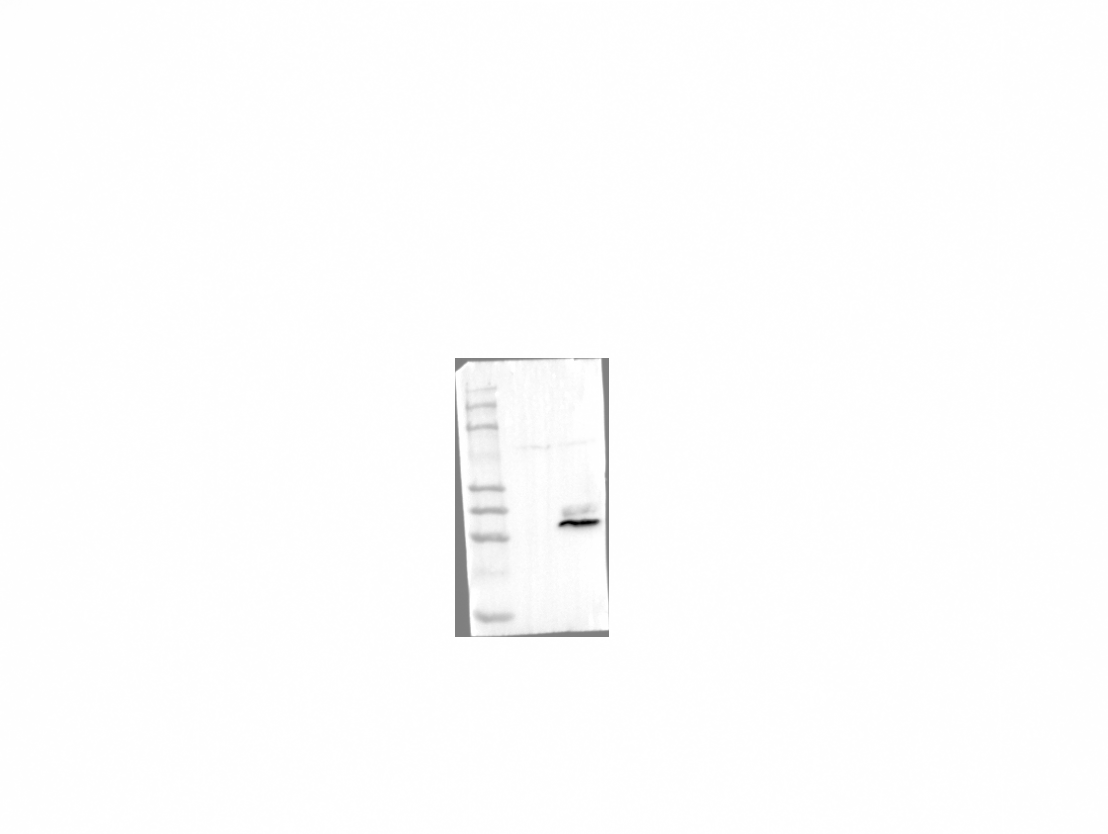

Supplement: Figure 4—source data 2. [file elife-96445-fig4-data2.zip › Figure 4_Source Data 2/4I/IB-SPI1.tif]

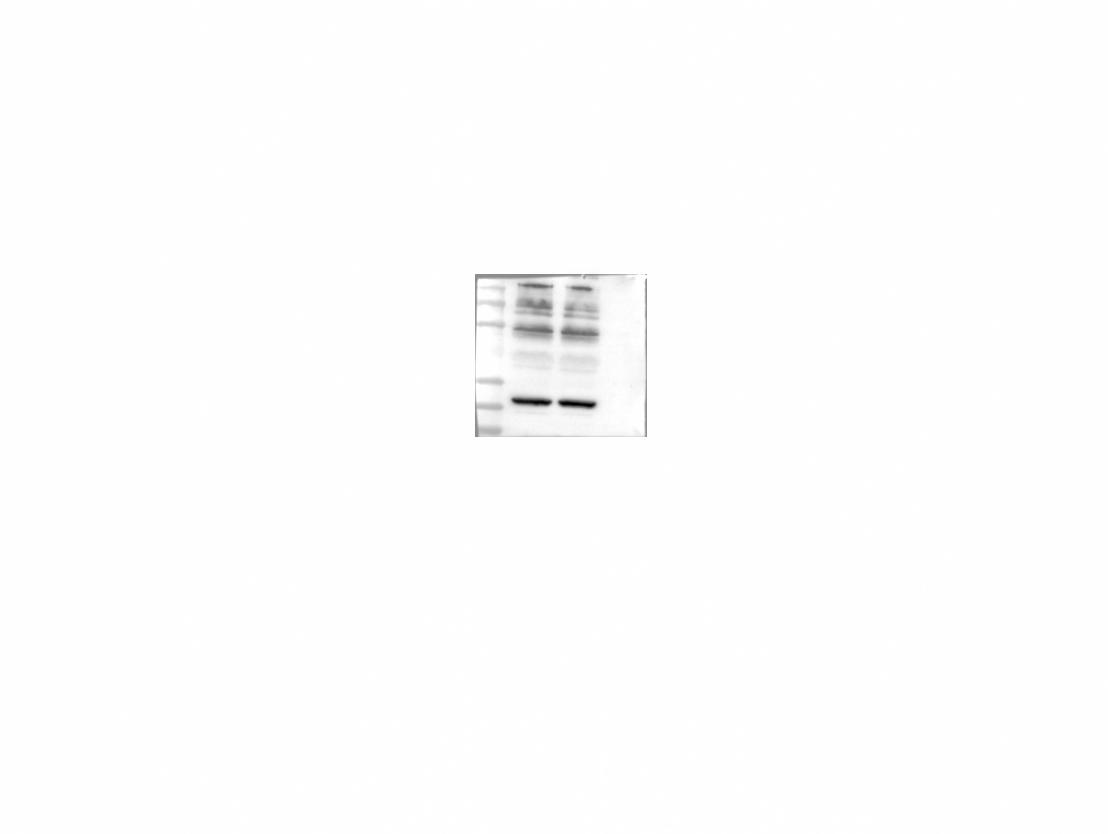

Supplement: Figure 4—source data 2. [file elife-96445-fig4-data2.zip › Figure 4_Source Data 2/4I/INPUT-actin.tif]

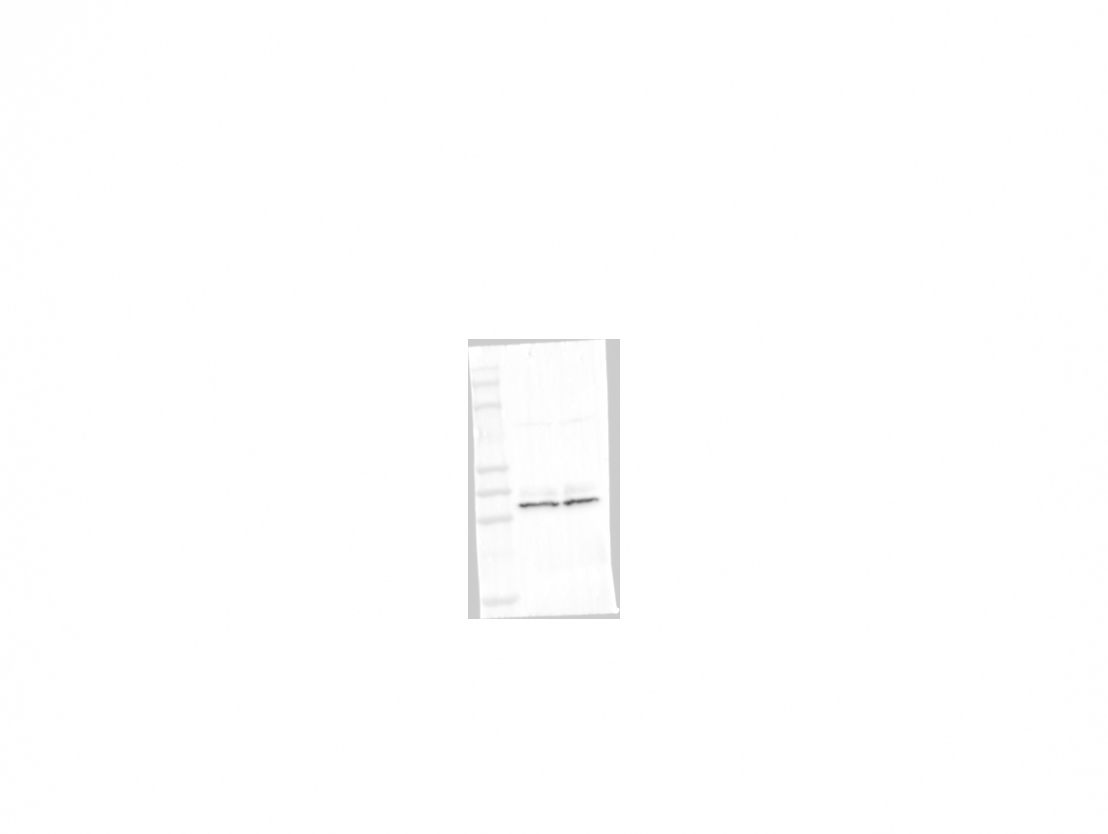

Supplement: Figure 4—source data 2. [file elife-96445-fig4-data2.zip › Figure 4_Source Data 2/4I/SPI1-INPUT.tif]

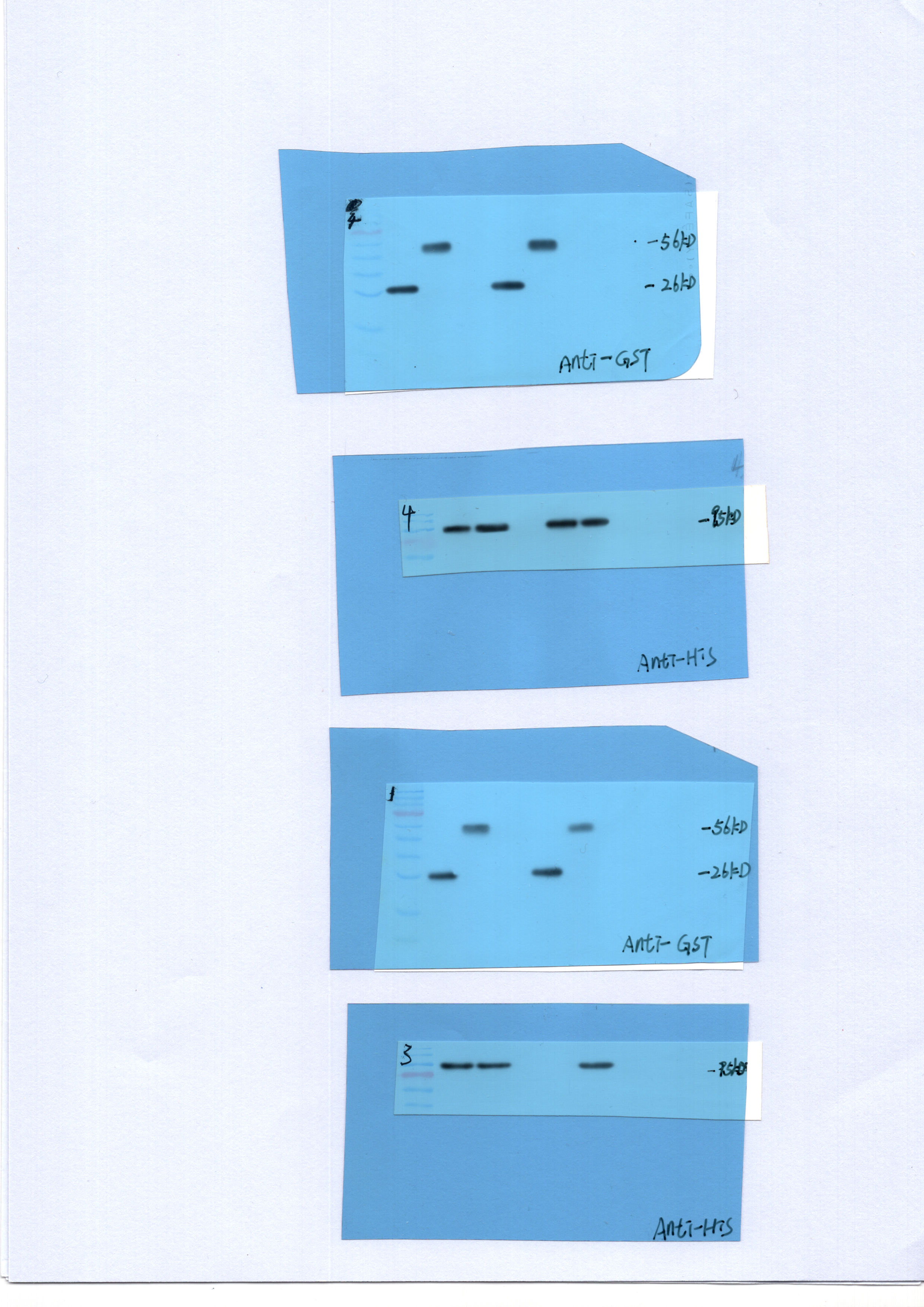

Supplement: Figure 4—source data 2. [file elife-96445-fig4-data2.zip › Figure 4_Source Data 2/4J/img286.bmp]

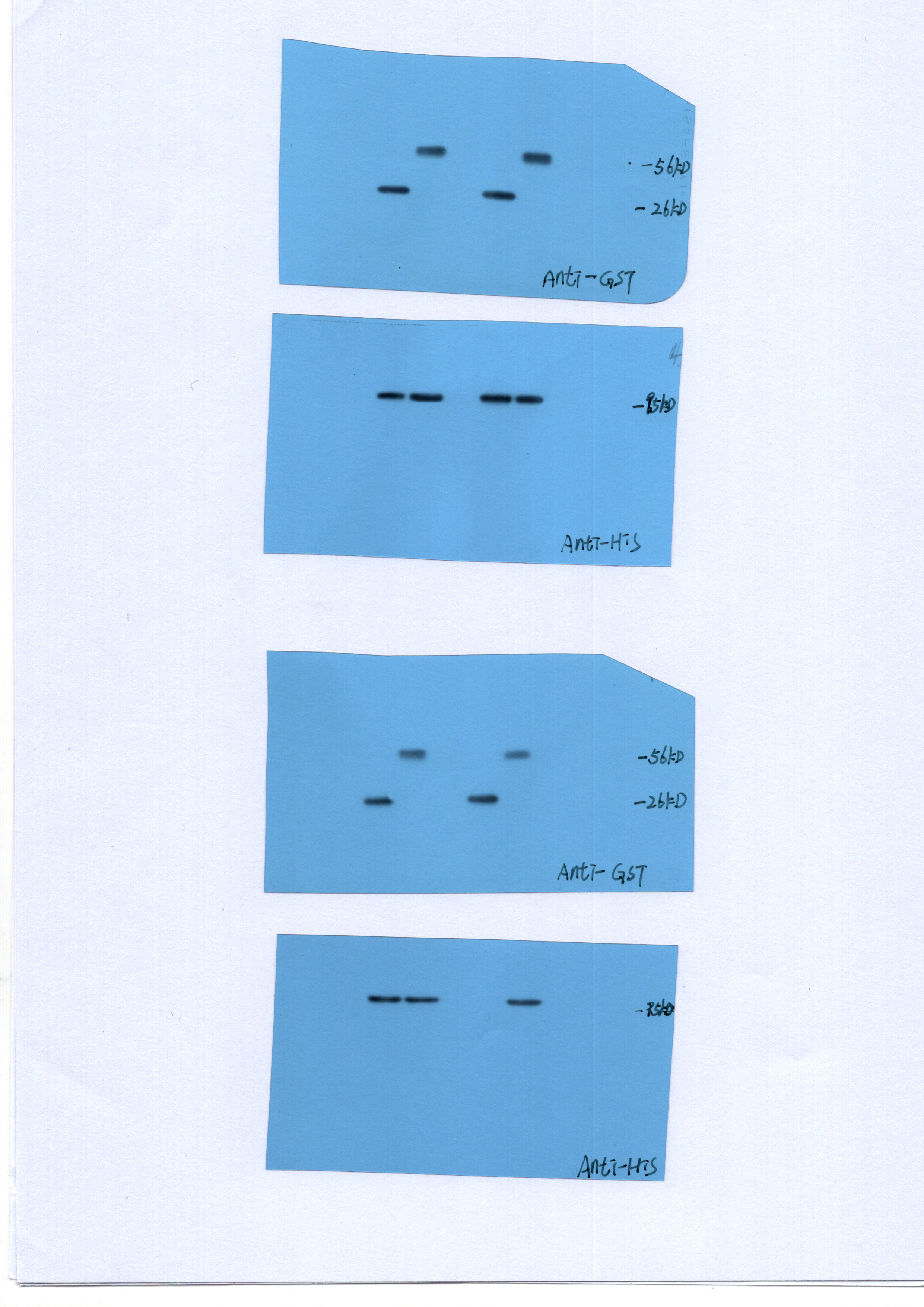

Supplement: Figure 4—source data 2. [file elife-96445-fig4-data2.zip › Figure 4_Source Data 2/4J/img287.bmp]

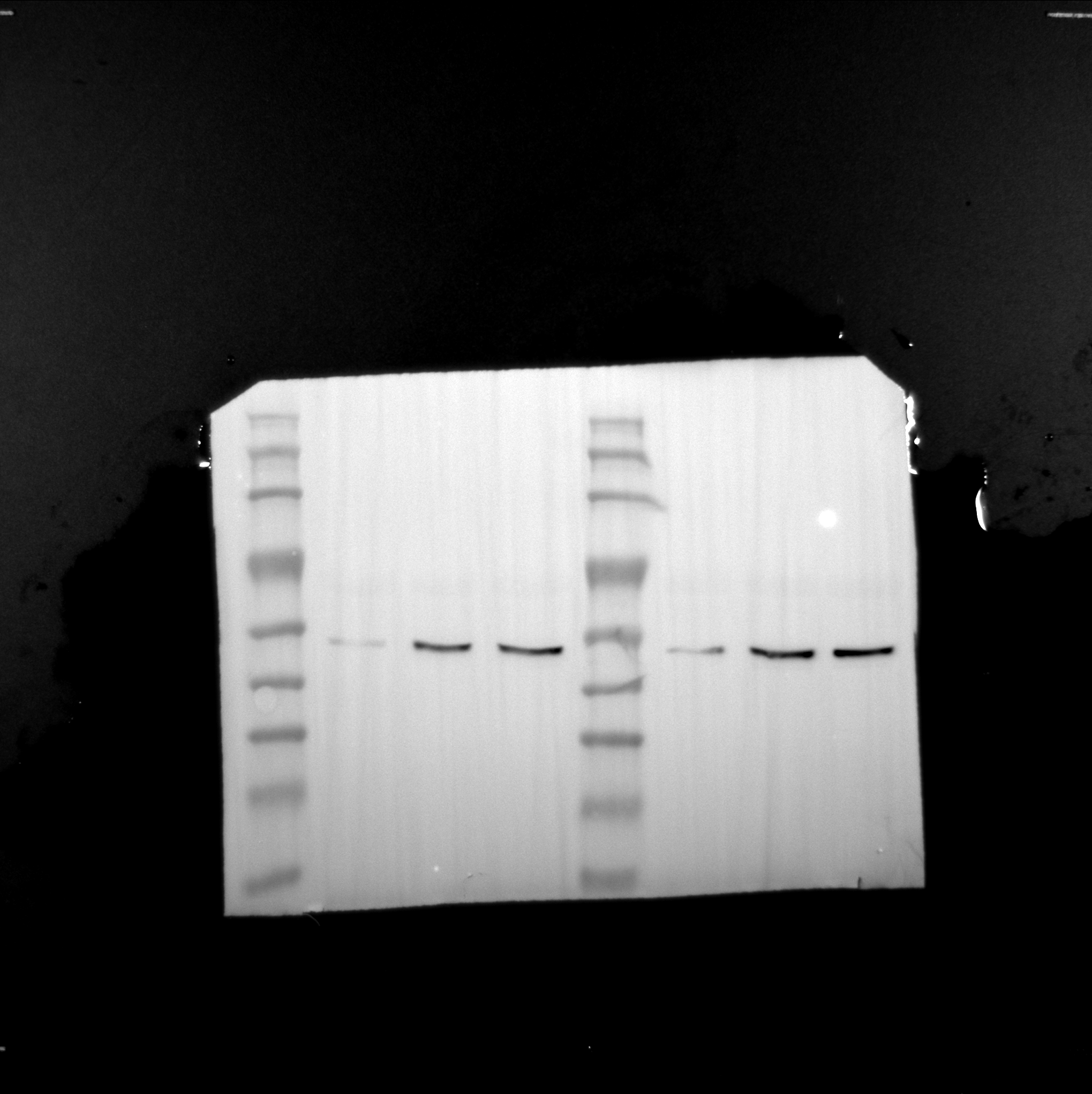

Supplement: Figure 5—source data 2. [file elife-96445-fig5-data2.zip › Figure 5_Source Data 2/5C/HDAC3.tif]

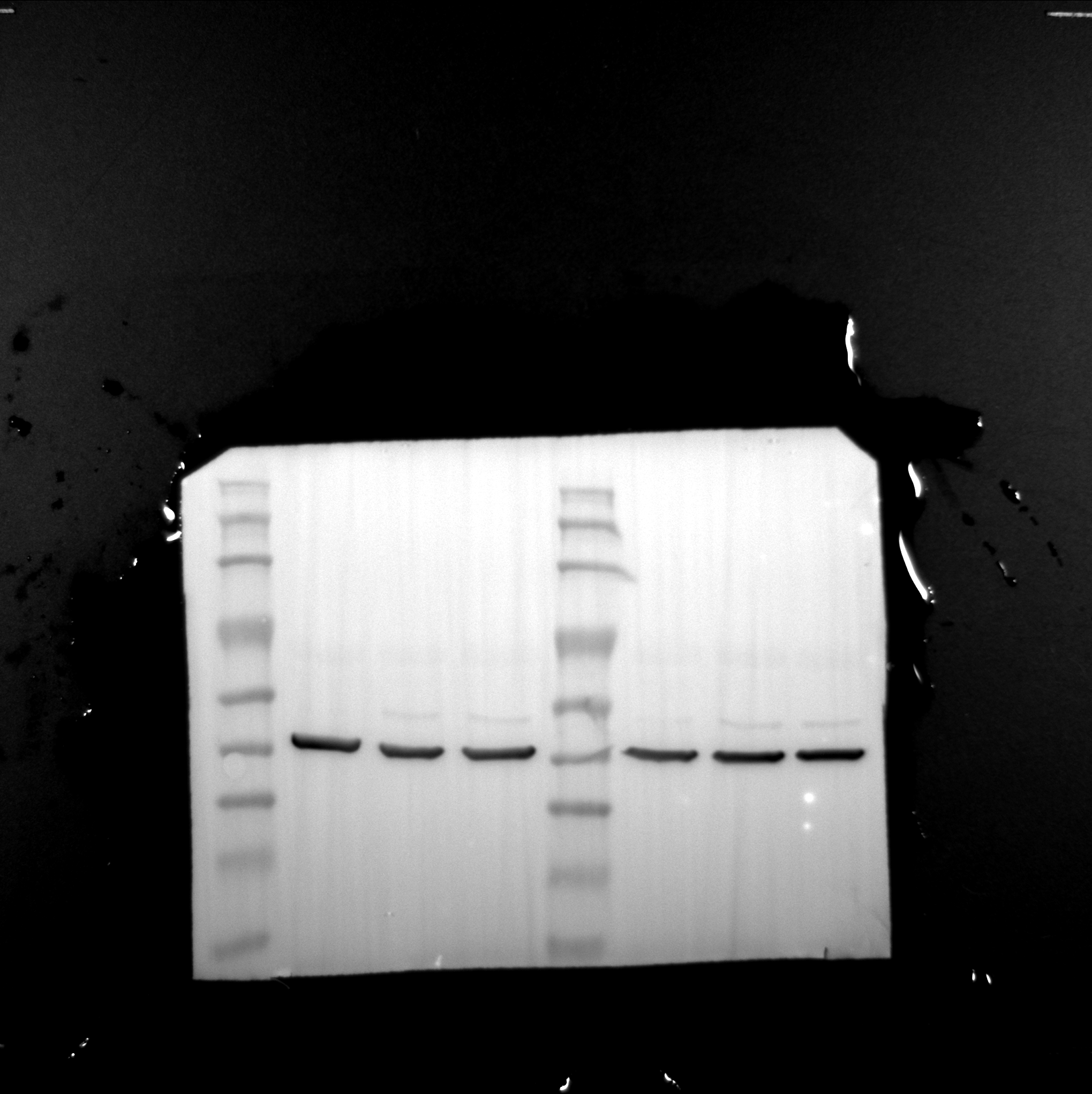

Supplement: Figure 5—source data 2. [file elife-96445-fig5-data2.zip › Figure 5_Source Data 2/5C/HDAC33ACTIIN.tif]

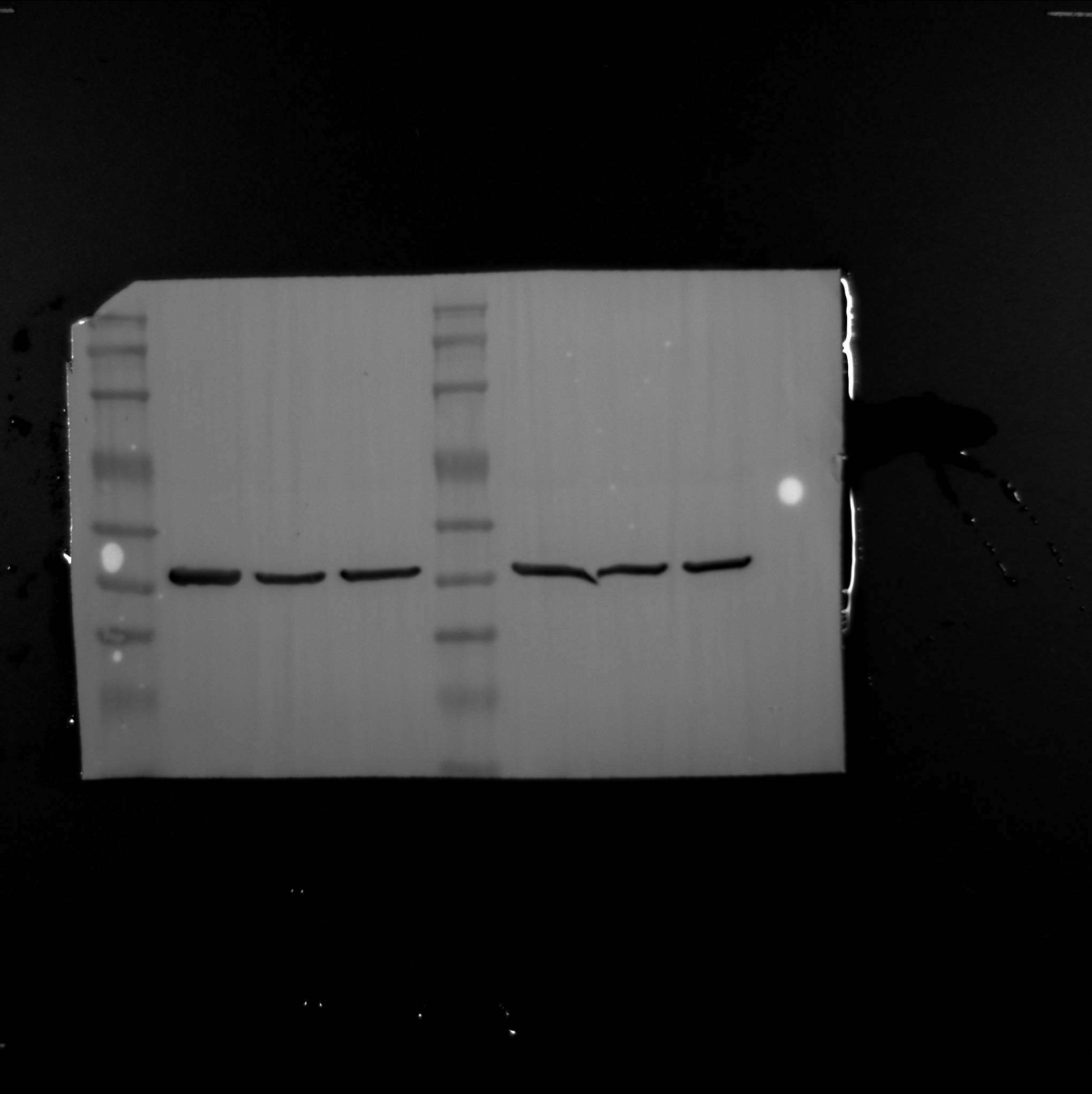

Supplement: Figure 5—source data 2. [file elife-96445-fig5-data2.zip › Figure 5_Source Data 2/5C/zfp-actin.tif]

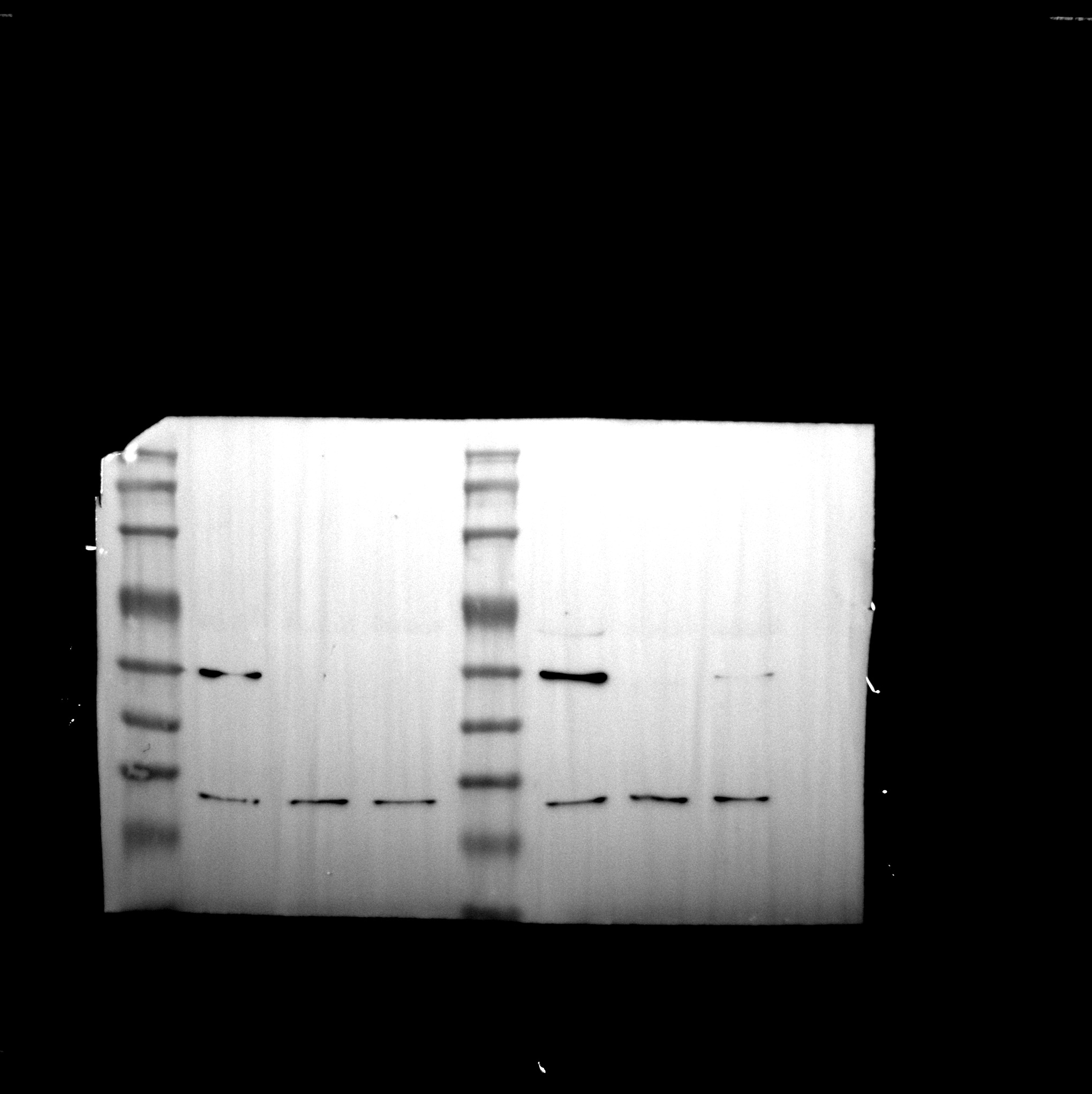

Supplement: Figure 5—source data 2. [file elife-96445-fig5-data2.zip › Figure 5_Source Data 2/5C/ZFP36L1.tif]

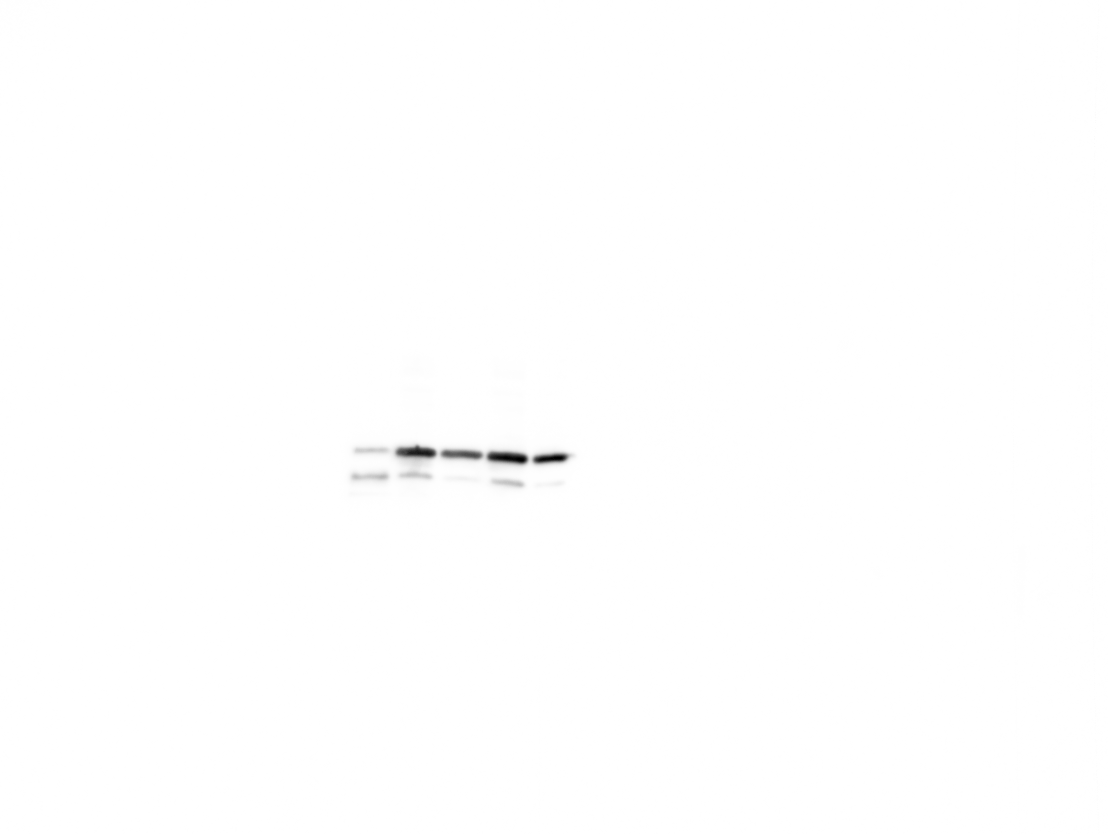

Supplement: Figure 5—source data 2. [file elife-96445-fig5-data2.zip › Figure 5_Source Data 2/5E/H3-3.tif]

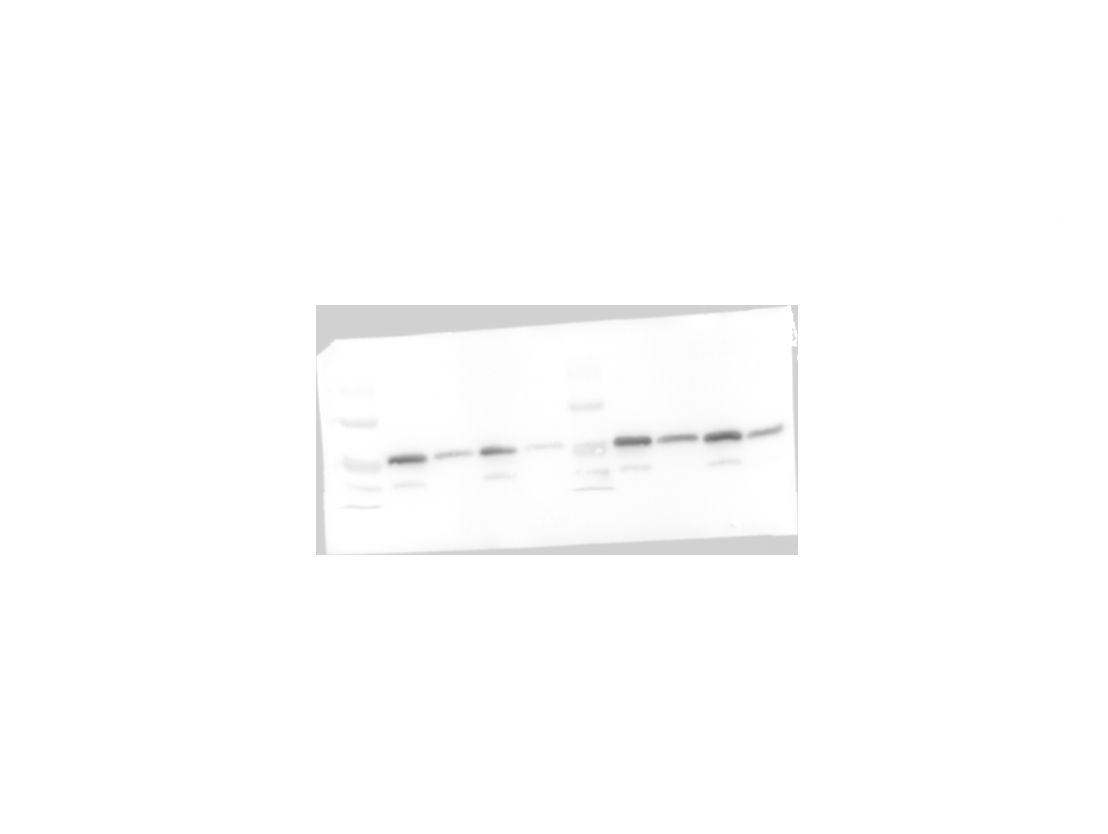

Supplement: Figure 5—source data 2. [file elife-96445-fig5-data2.zip › Figure 5_Source Data 2/5E/H3K27.tif]

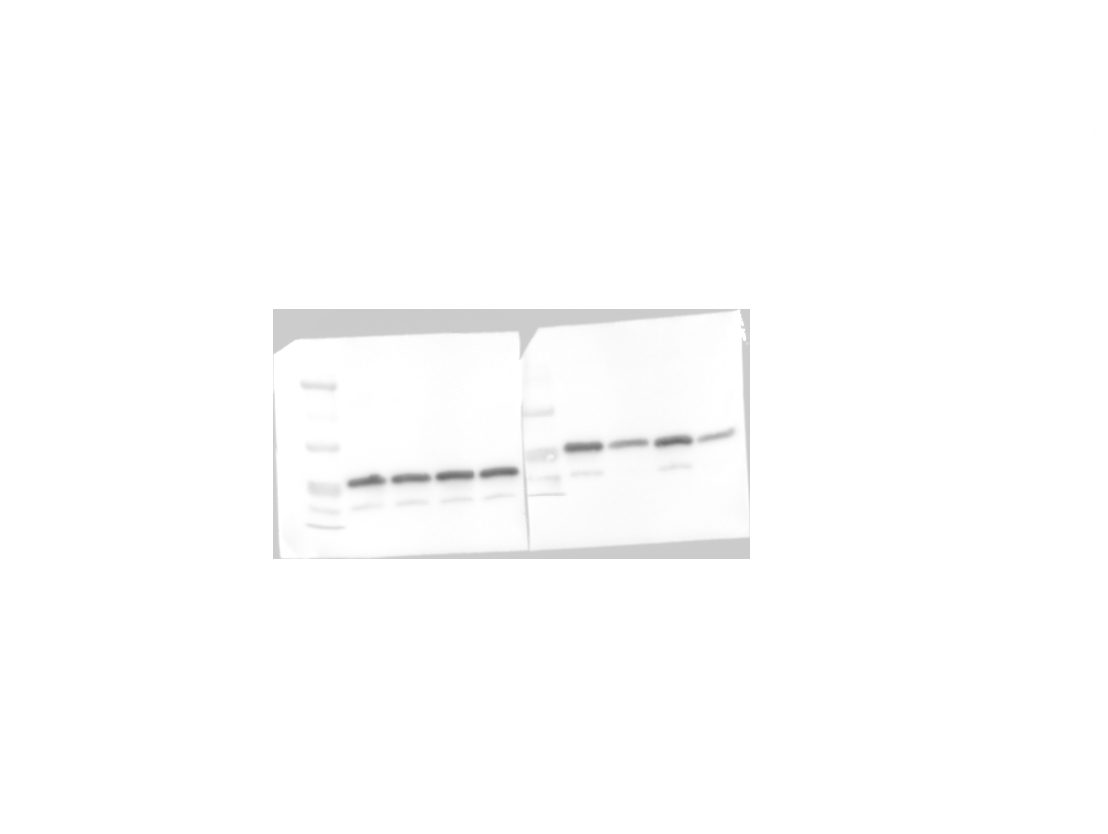

Supplement: Figure 5—source data 2. [file elife-96445-fig5-data2.zip › Figure 5_Source Data 2/5E/H3和H3K.tif]

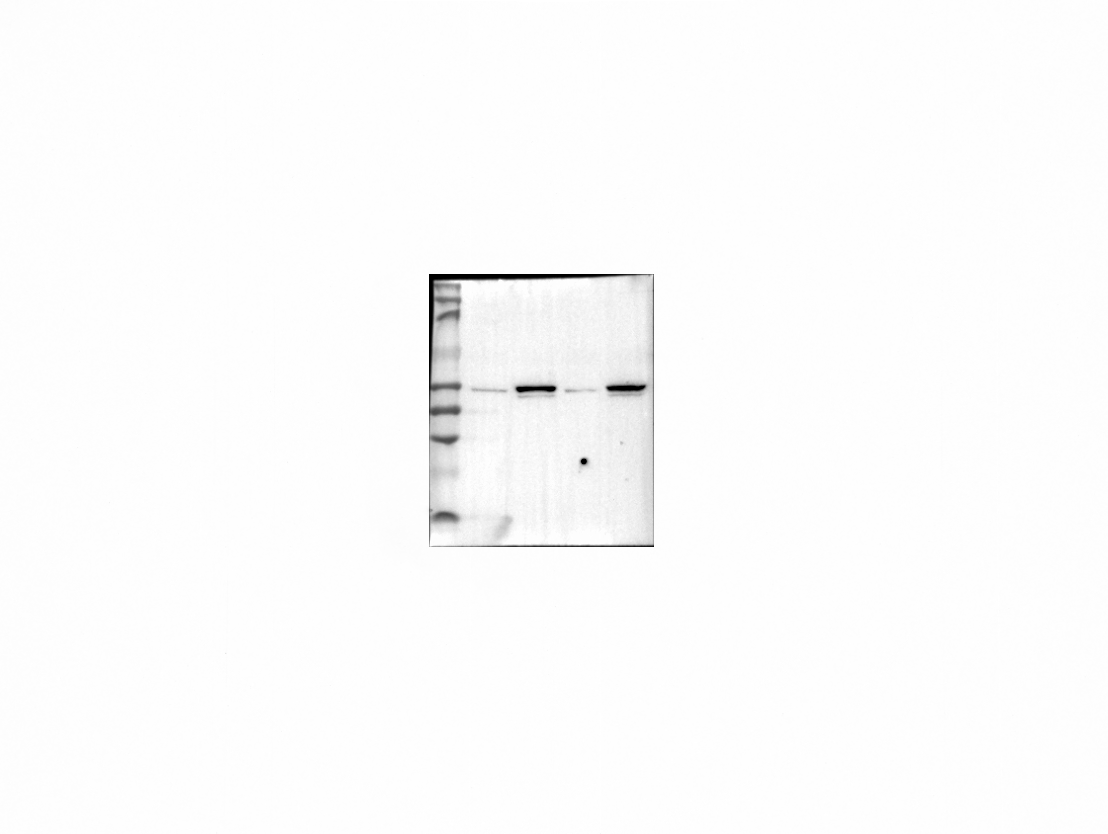

Supplement: Figure 5—source data 2. [file elife-96445-fig5-data2.zip › Figure 5_Source Data 2/5E/hdac3.tif]

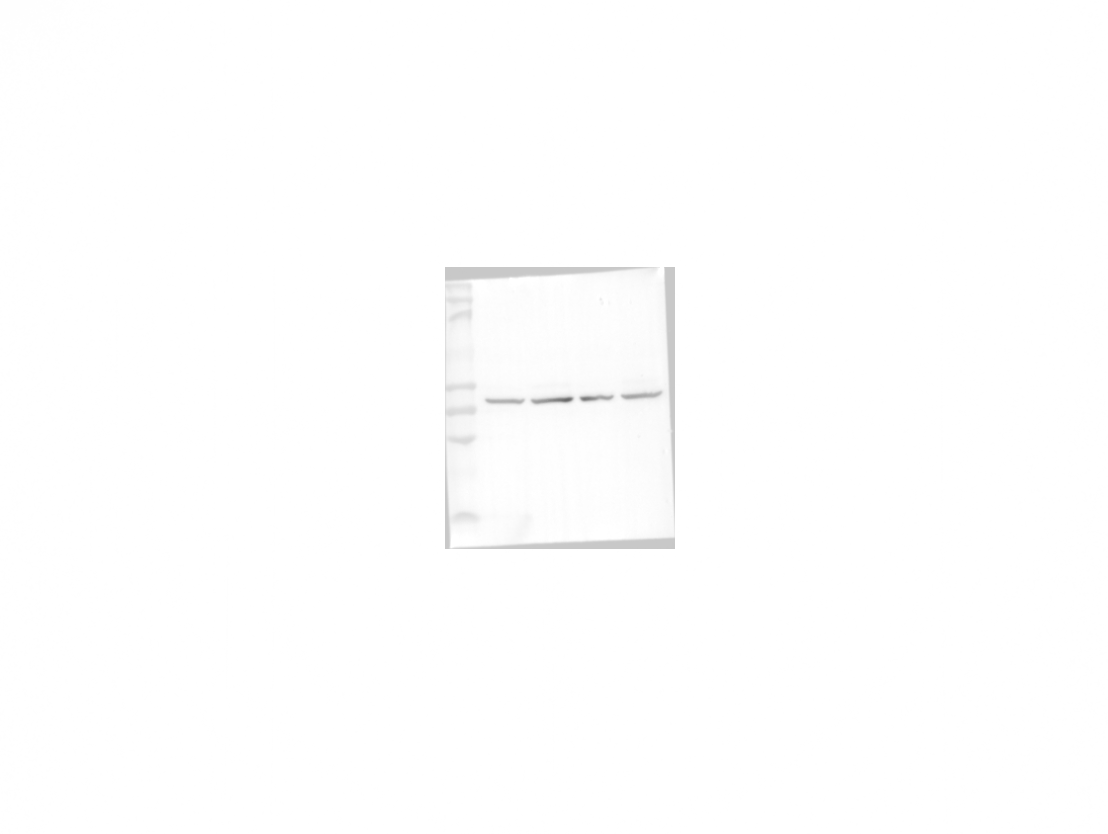

Supplement: Figure 5—source data 2. [file elife-96445-fig5-data2.zip › Figure 5_Source Data 2/5E/hdac3actin.tif]

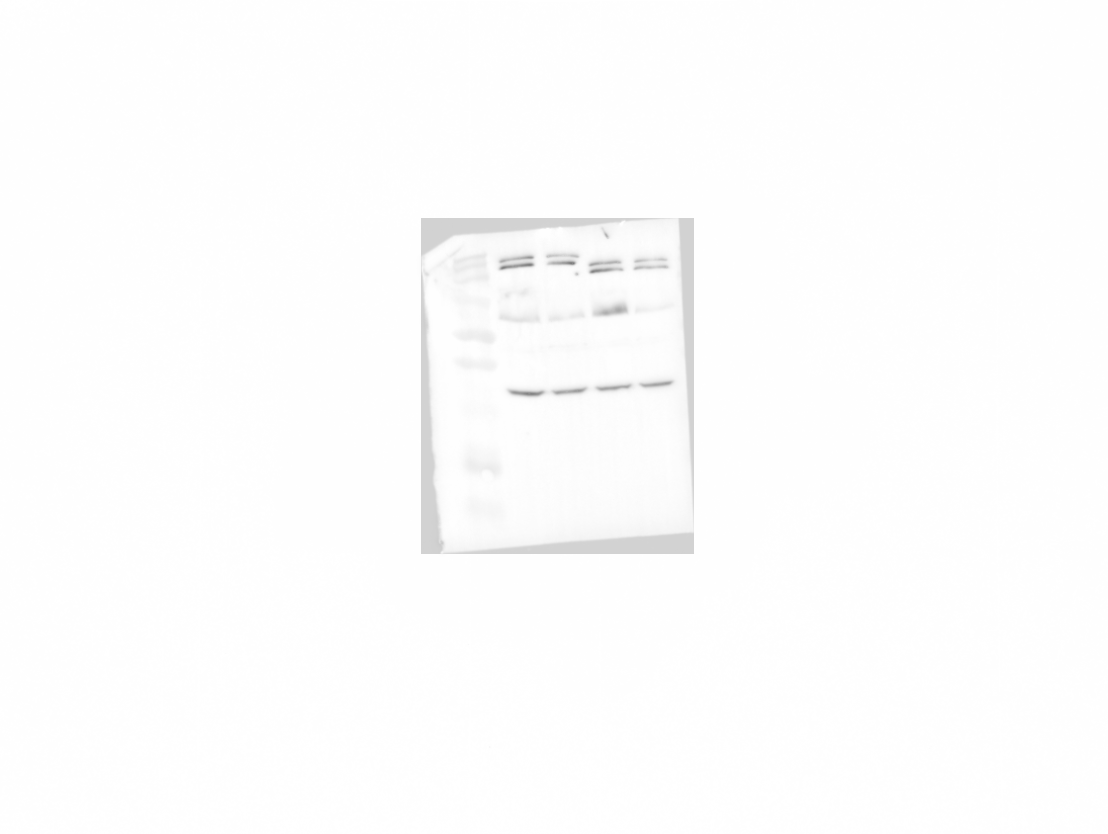

Supplement: Figure 5—source data 2. [file elife-96445-fig5-data2.zip › Figure 5_Source Data 2/5E/PD-L1.tif]

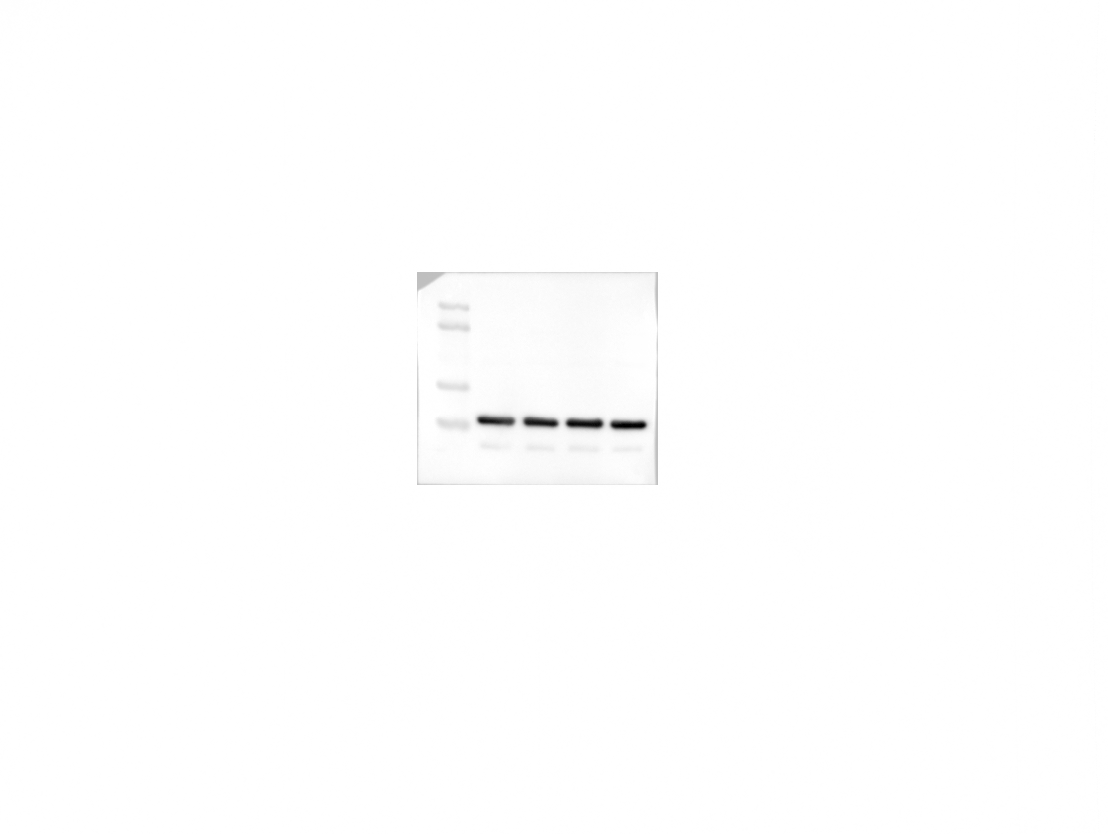

Supplement: Figure 5—source data 2. [file elife-96445-fig5-data2.zip › Figure 5_Source Data 2/5G/H3-3.21.tif]

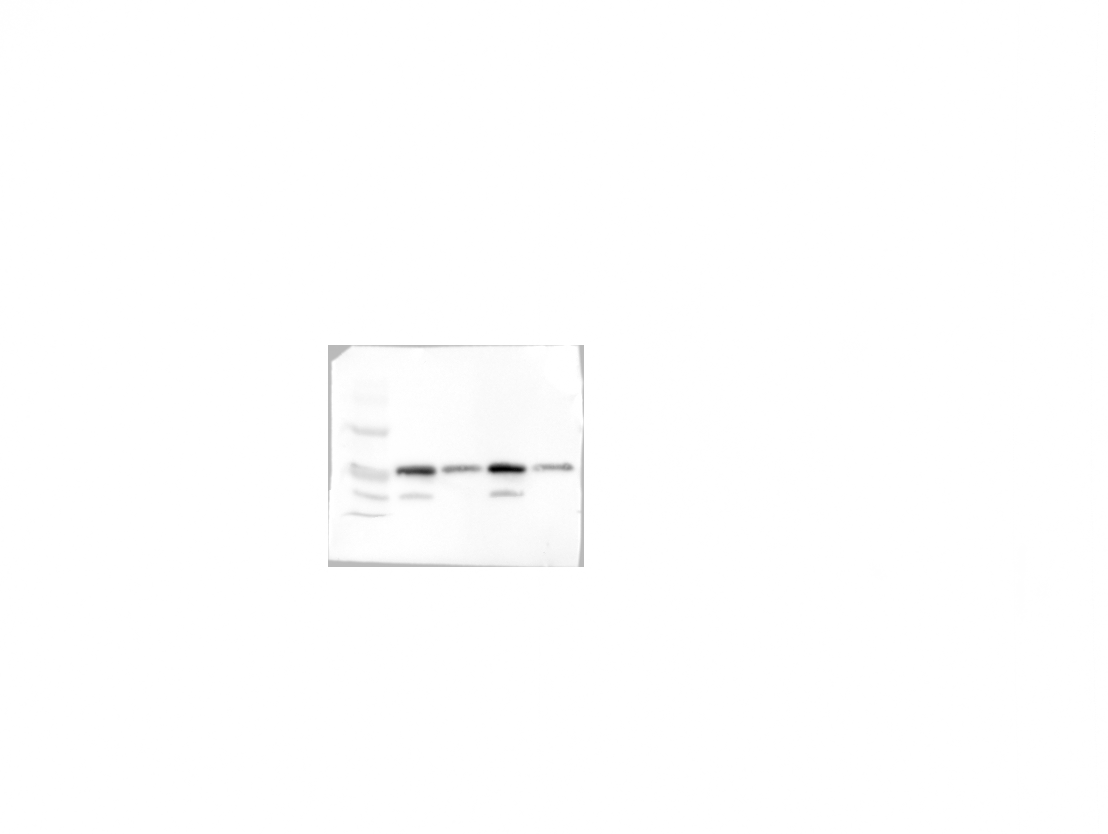

Supplement: Figure 5—source data 2. [file elife-96445-fig5-data2.zip › Figure 5_Source Data 2/5G/H3K27ac.tif]

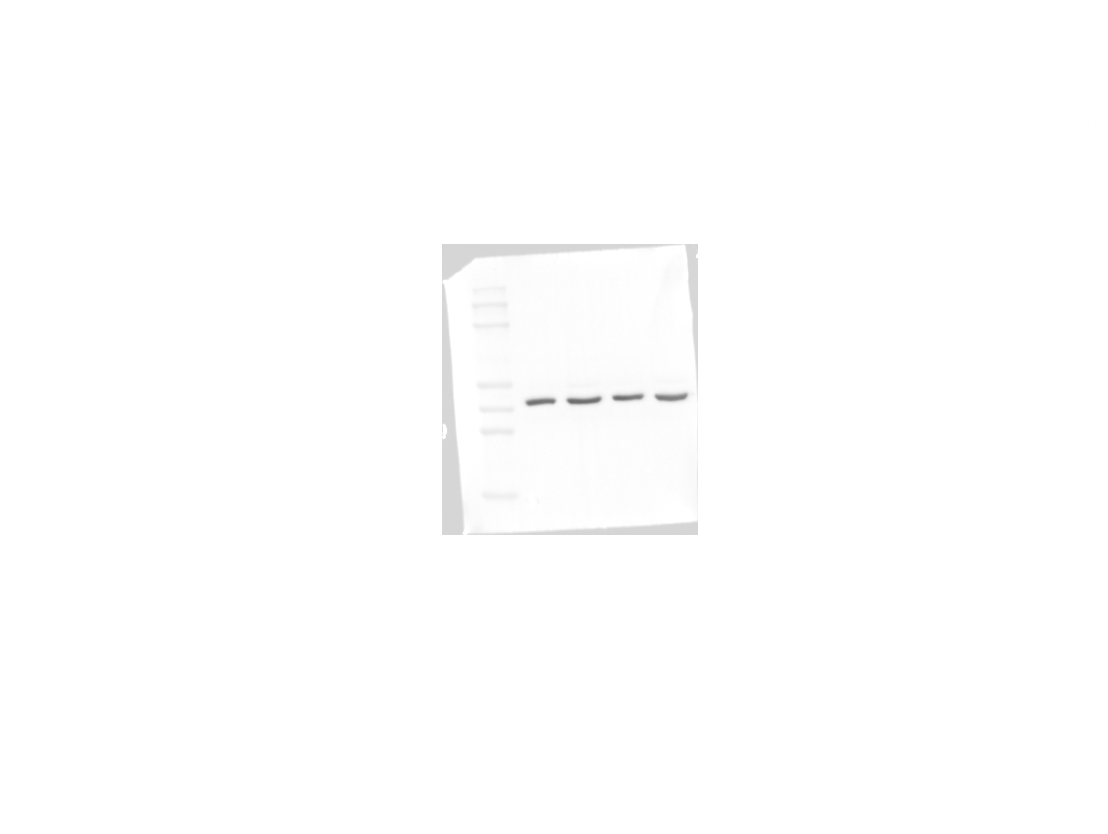

Supplement: Figure 5—source data 2. [file elife-96445-fig5-data2.zip › Figure 5_Source Data 2/5G/HDAC3-1-ACTIN.tif]

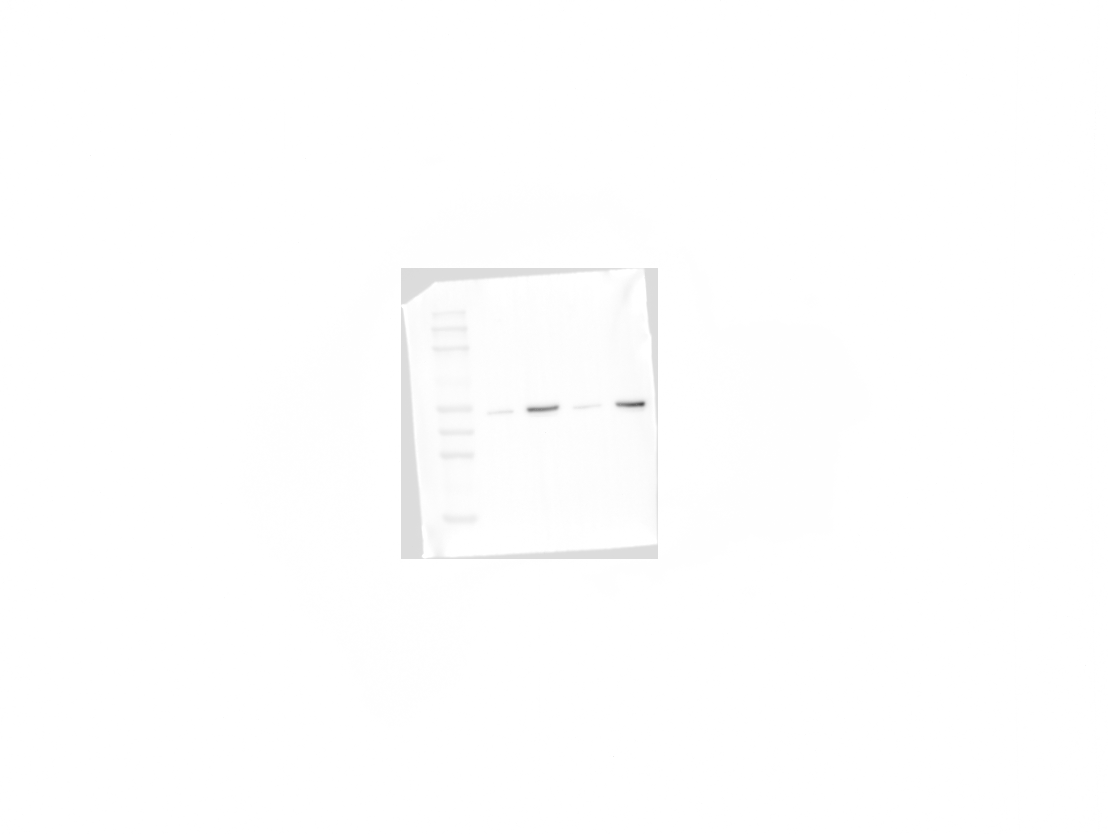

Supplement: Figure 5—source data 2. [file elife-96445-fig5-data2.zip › Figure 5_Source Data 2/5G/HDAC3-1.tif]

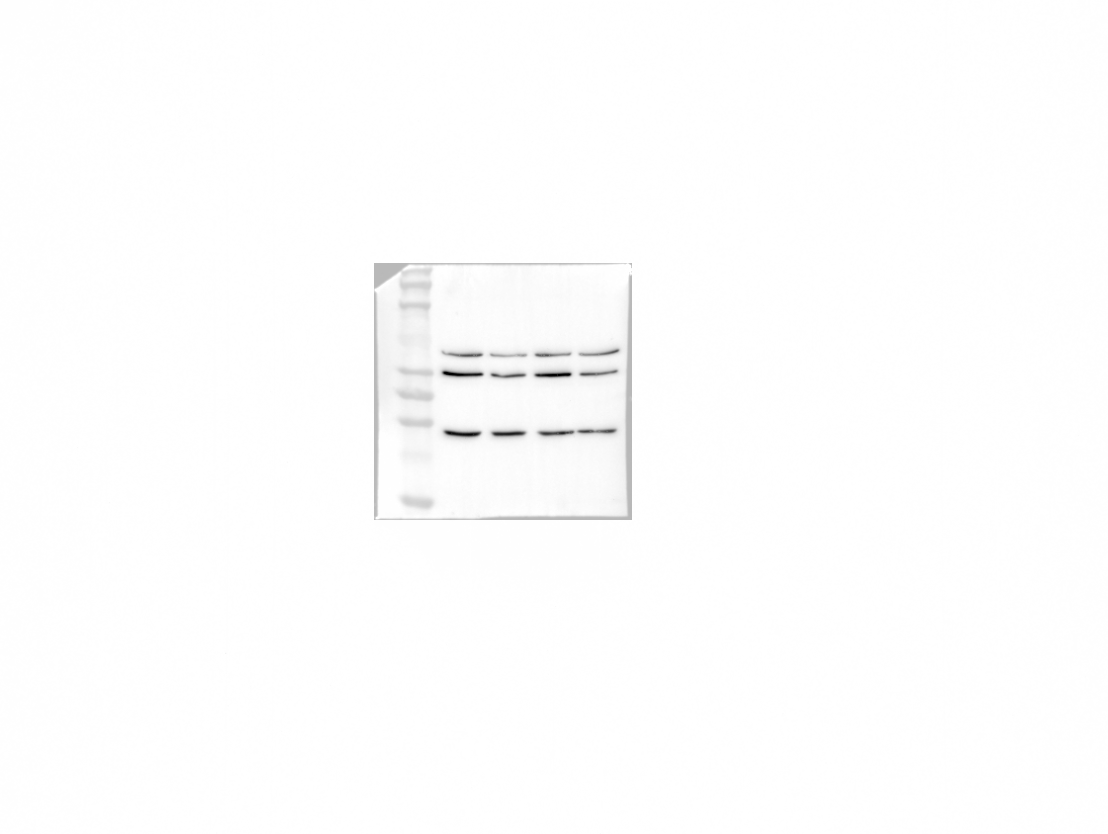

Supplement: Figure 5—source data 2. [file elife-96445-fig5-data2.zip › Figure 5_Source Data 2/5G/zfp36l1.tif]

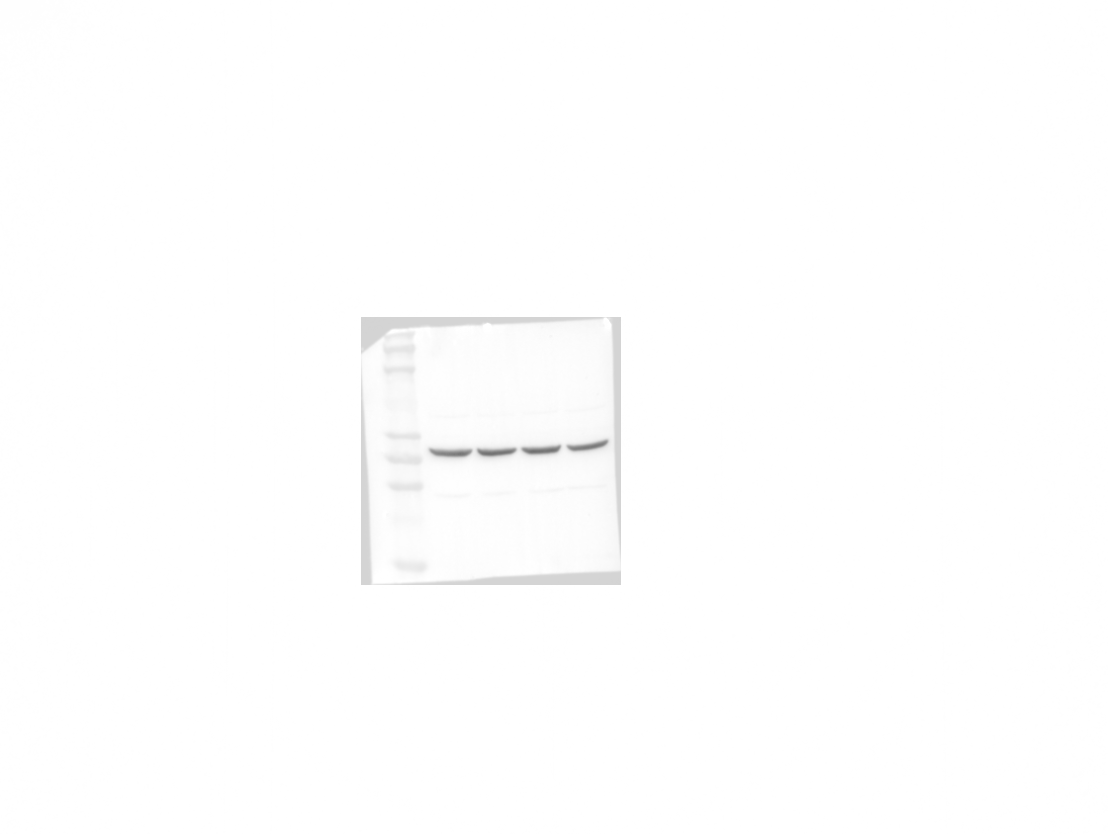

Supplement: Figure 5—source data 2. [file elife-96445-fig5-data2.zip › Figure 5_Source Data 2/5G/zfpactin.tif]

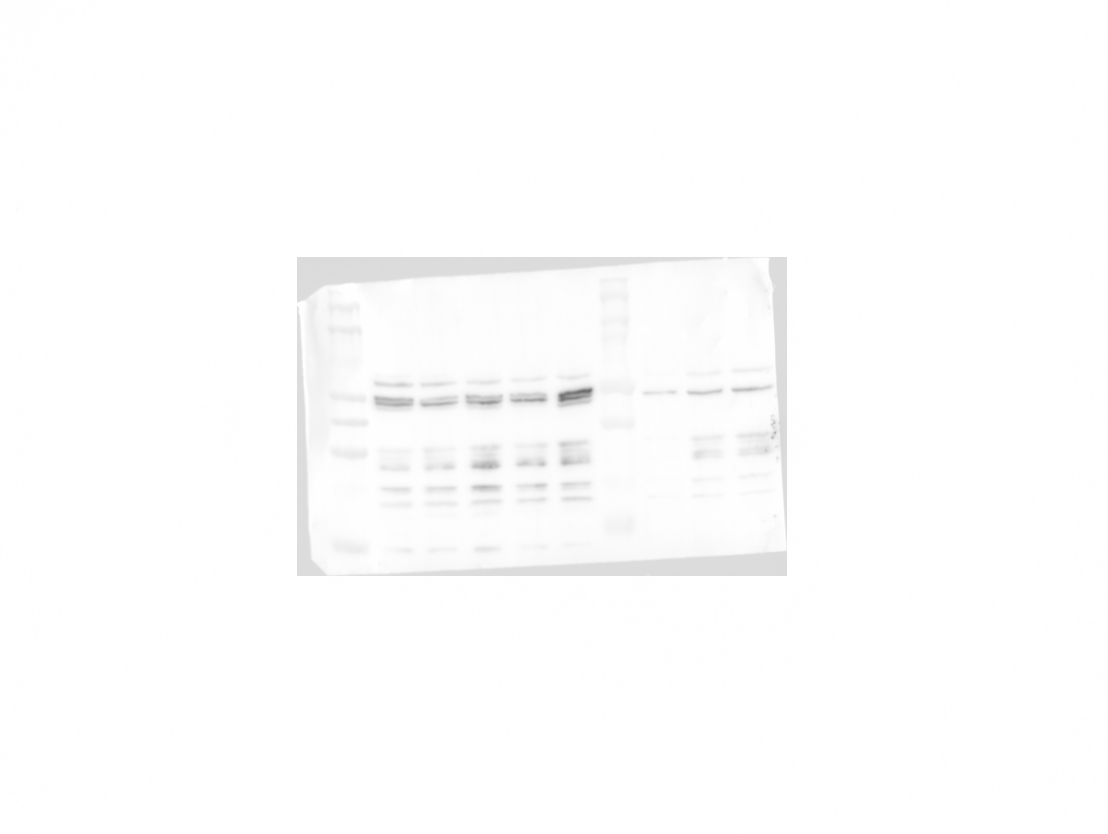

Supplement: Figure 5—source data 2. [file elife-96445-fig5-data2.zip › Figure 5_Source Data 2/5I/MGC803/HDAC3.tif]

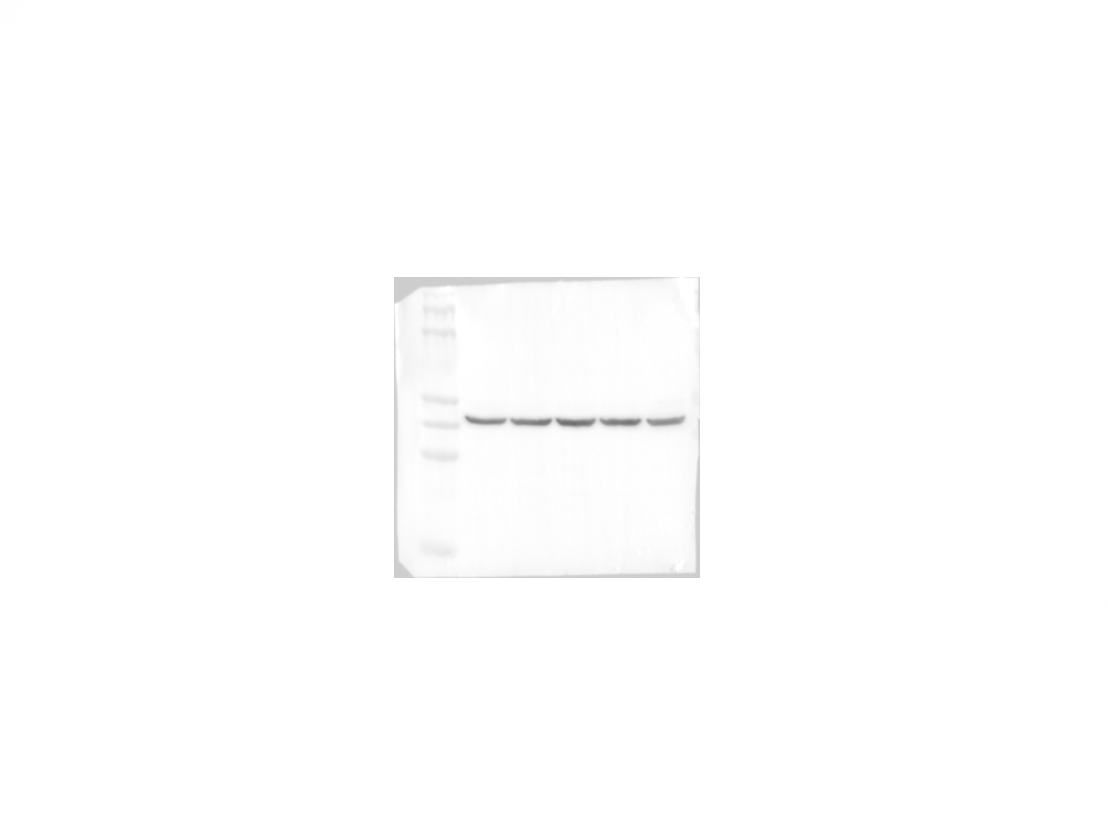

Supplement: Figure 5—source data 2. [file elife-96445-fig5-data2.zip › Figure 5_Source Data 2/5I/MGC803/hdac3actin.tif]

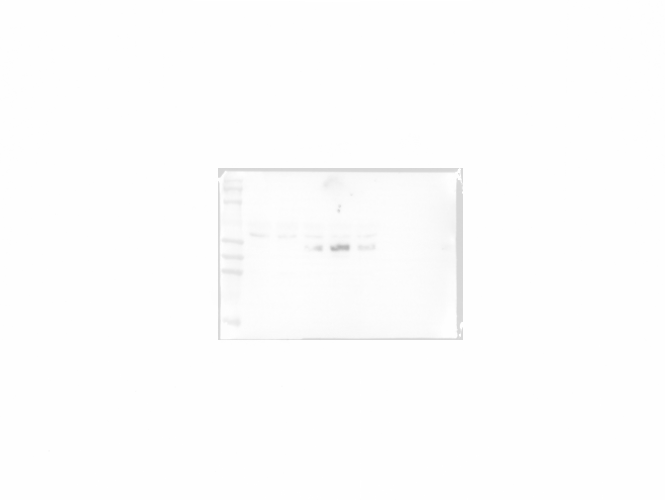

Supplement: Figure 5—source data 2. [file elife-96445-fig5-data2.zip › Figure 5_Source Data 2/5I/MGC803/PD-L1.tif]

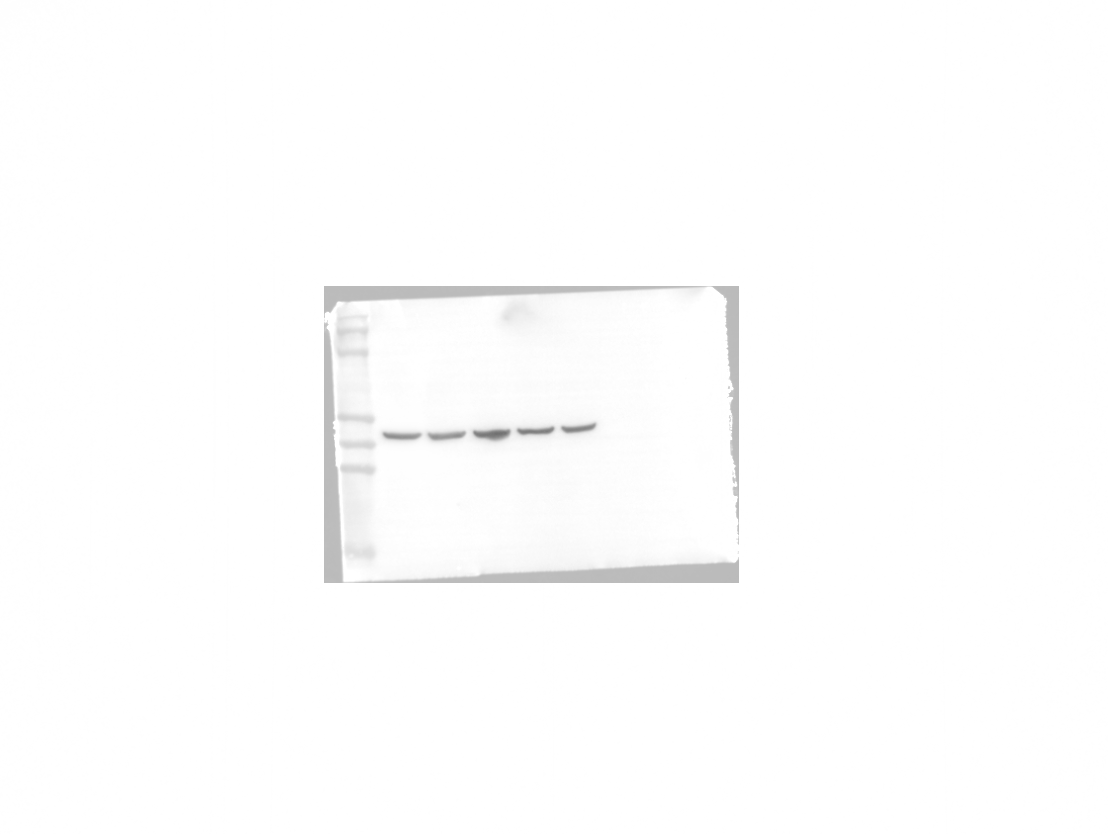

Supplement: Figure 5—source data 2. [file elife-96445-fig5-data2.zip › Figure 5_Source Data 2/5I/MGC803/pd-l1actin.tif]

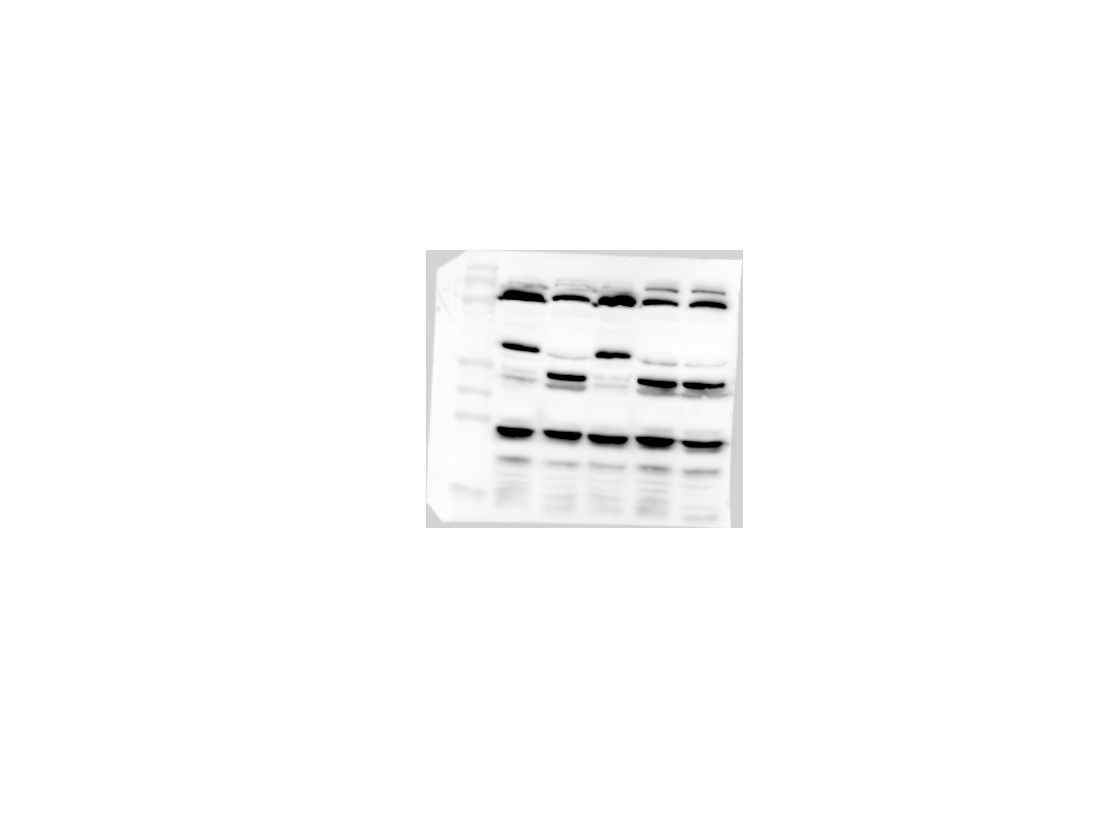

Supplement: Figure 5—source data 2. [file elife-96445-fig5-data2.zip › Figure 5_Source Data 2/5I/MGC803/zfp36l1.tif]

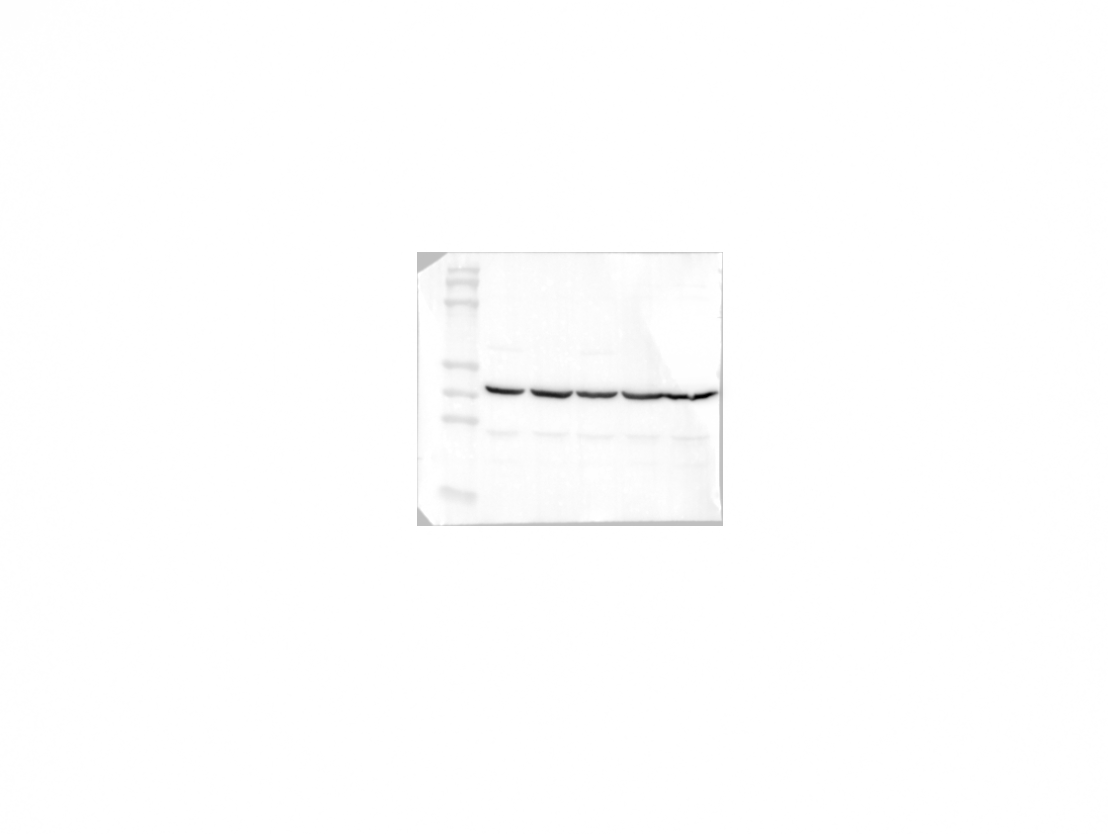

Supplement: Figure 5—source data 2. [file elife-96445-fig5-data2.zip › Figure 5_Source Data 2/5I/MGC803/zfpactin.tif]

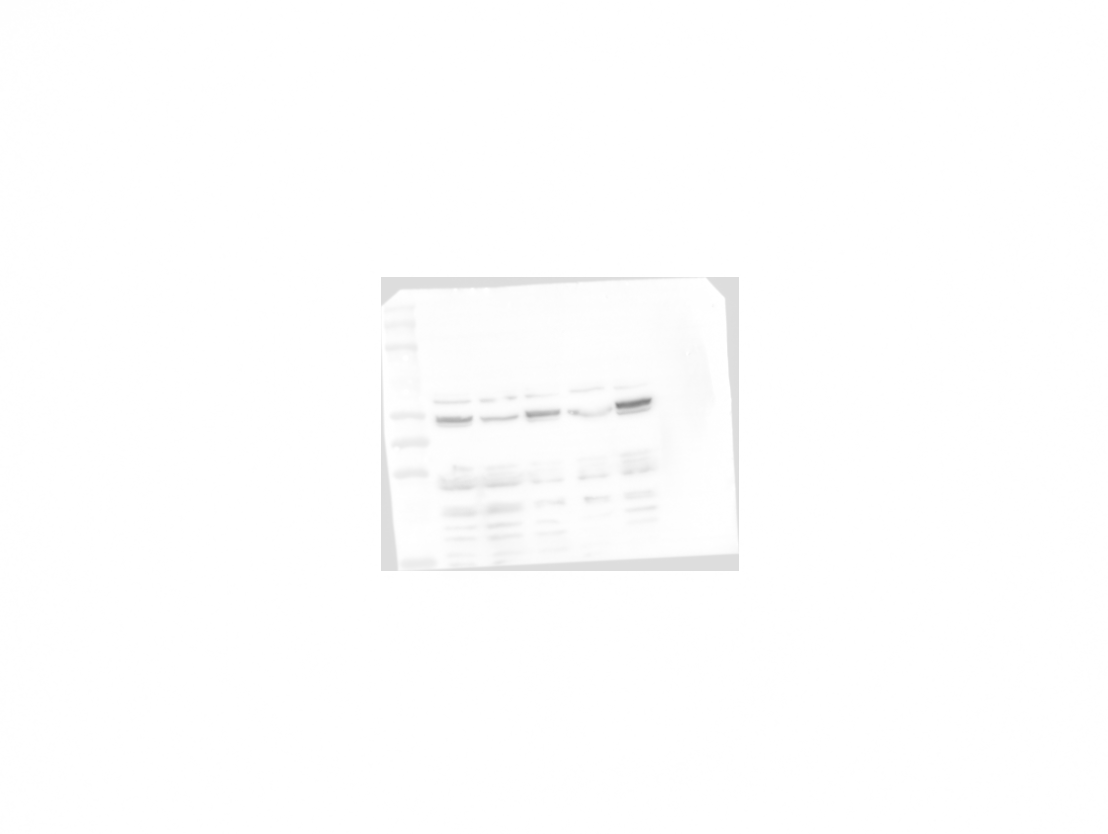

Supplement: Figure 5—source data 2. [file elife-96445-fig5-data2.zip › Figure 5_Source Data 2/5I/XGC-2/HDAC3.tif]

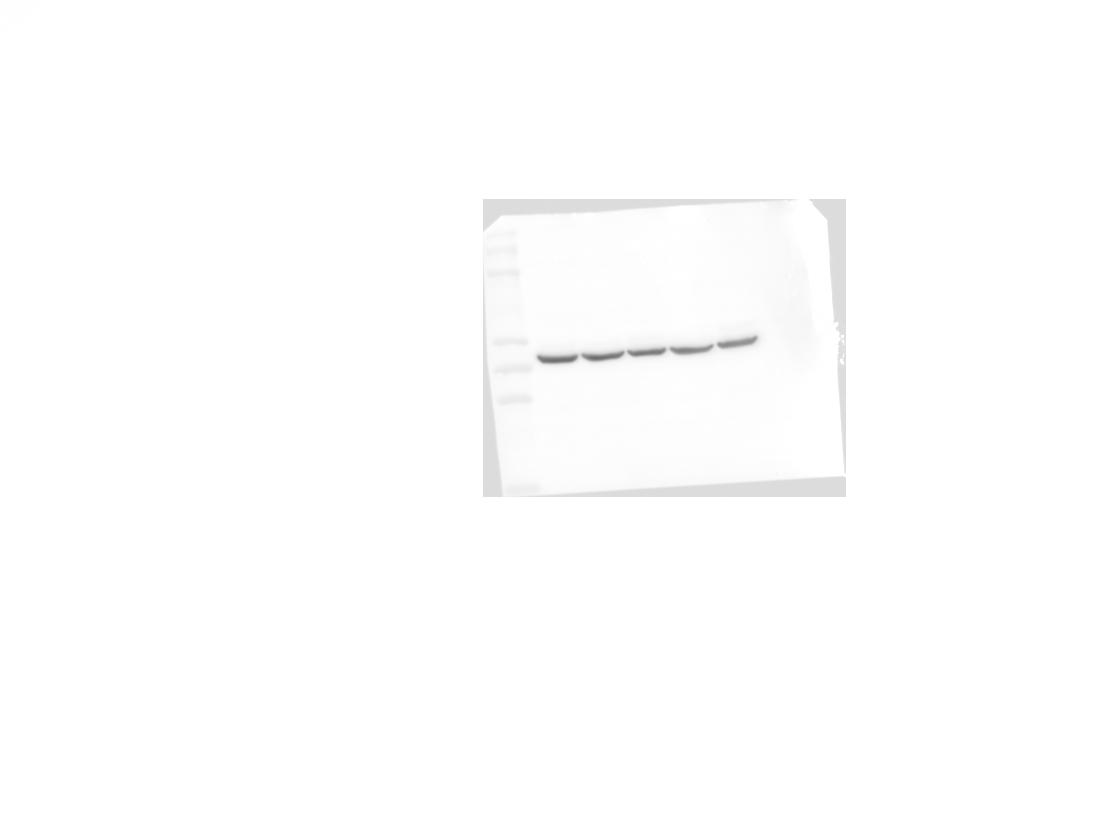

Supplement: Figure 5—source data 2. [file elife-96445-fig5-data2.zip › Figure 5_Source Data 2/5I/XGC-2/HDAC3actin.tif]

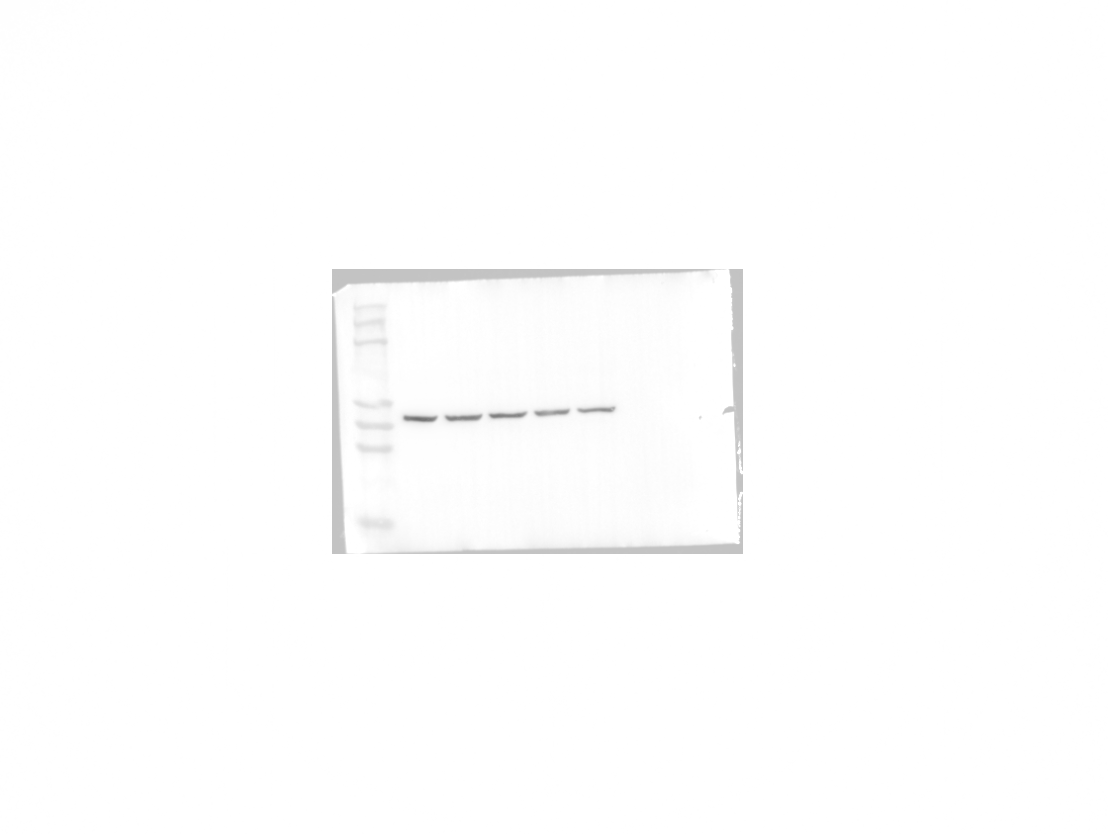

Supplement: Figure 5—source data 2. [file elife-96445-fig5-data2.zip › Figure 5_Source Data 2/5I/XGC-2/pd-l1 actin.tif]

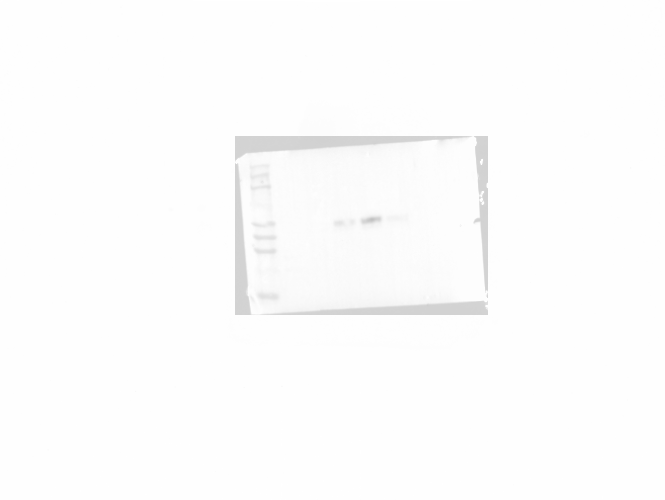

Supplement: Figure 5—source data 2. [file elife-96445-fig5-data2.zip › Figure 5_Source Data 2/5I/XGC-2/pd-l1.tif]

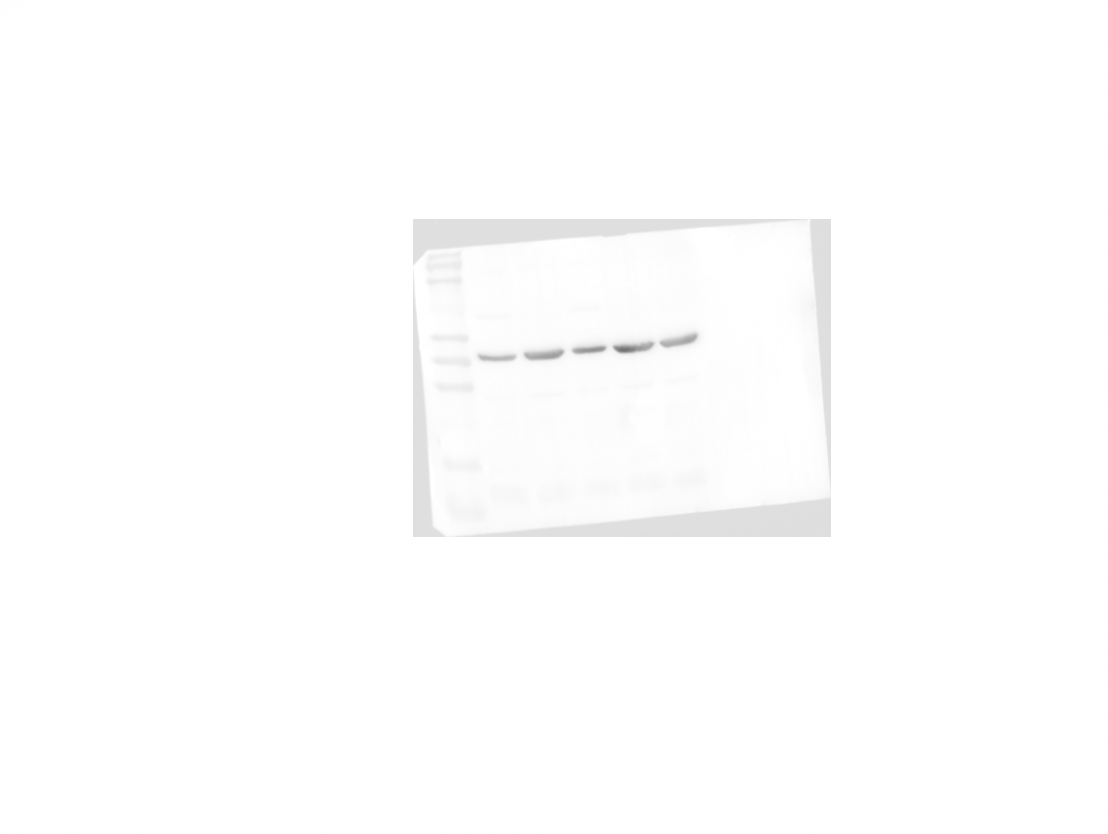

Supplement: Figure 5—source data 2. [file elife-96445-fig5-data2.zip › Figure 5_Source Data 2/5I/XGC-2/zfactin.tif]

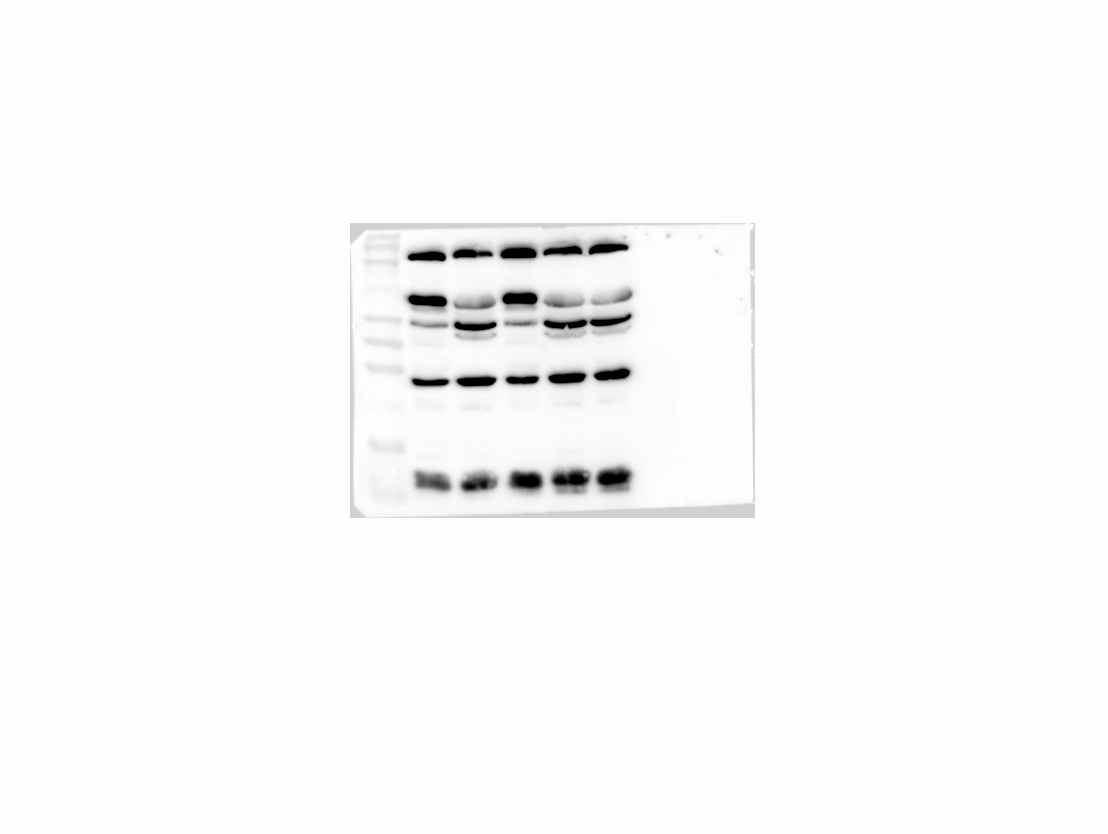

Supplement: Figure 5—source data 2. [file elife-96445-fig5-data2.zip › Figure 5_Source Data 2/5I/XGC-2/zfp.tif]

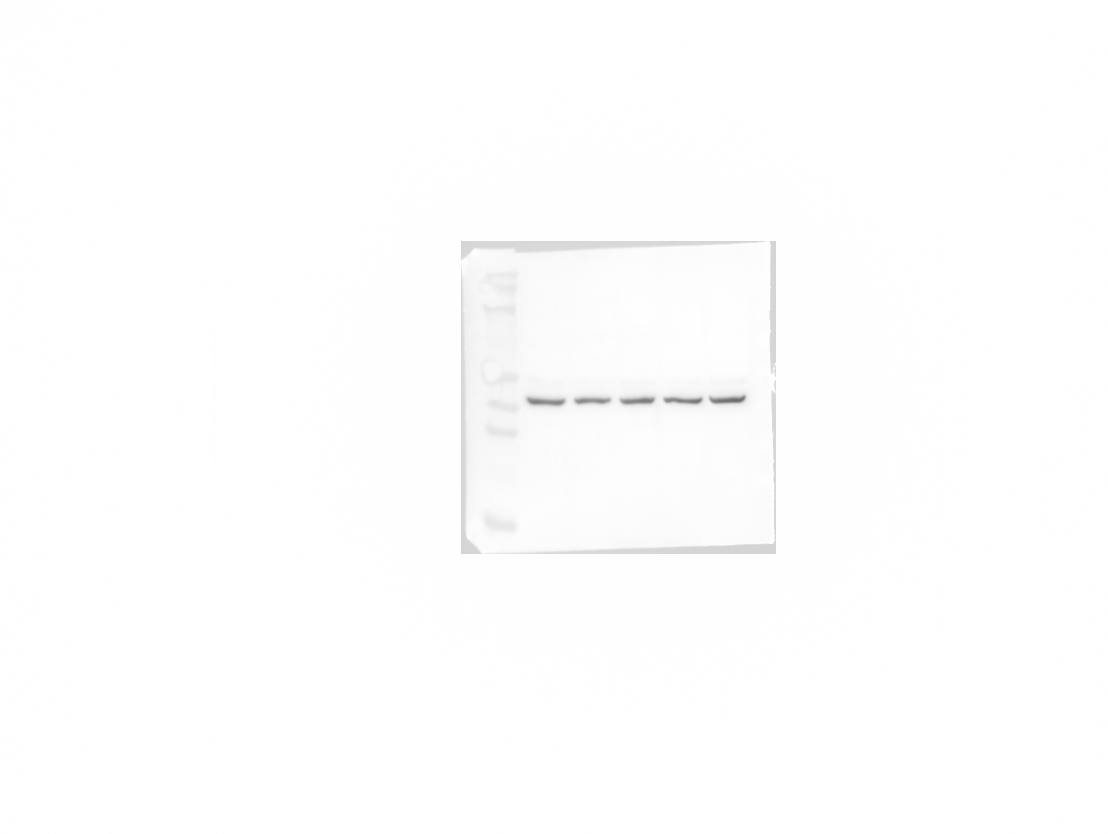

Supplement: Figure 5—source data 2. [file elife-96445-fig5-data2.zip › Figure 5_Source Data 2/5K/MGC803/hdac3 actin.tif]

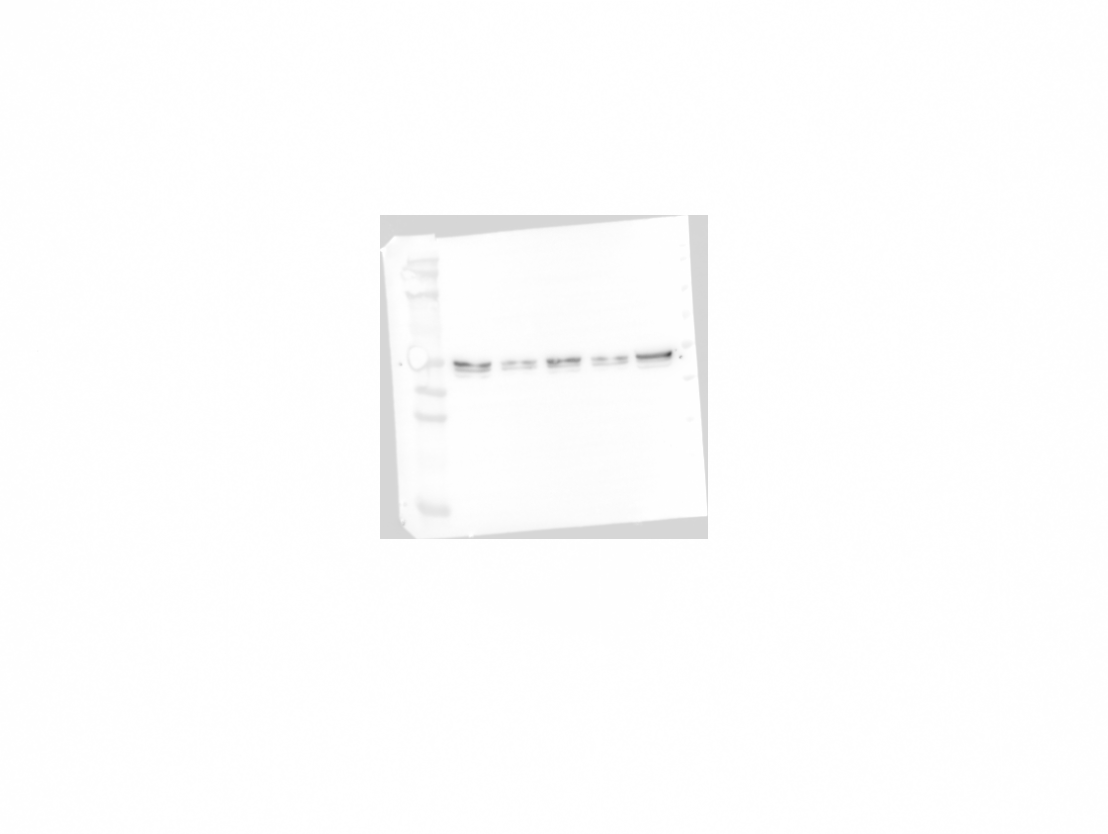

Supplement: Figure 5—source data 2. [file elife-96445-fig5-data2.zip › Figure 5_Source Data 2/5K/MGC803/HDAC3.tif]

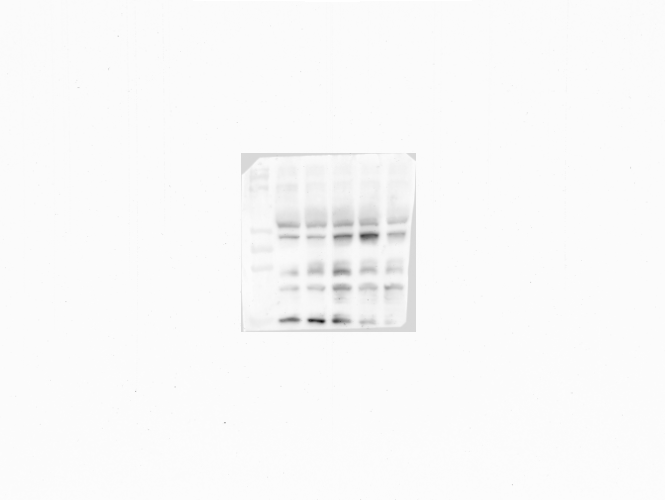

Supplement: Figure 5—source data 2. [file elife-96445-fig5-data2.zip › Figure 5_Source Data 2/5K/MGC803/PD-L1.tif]

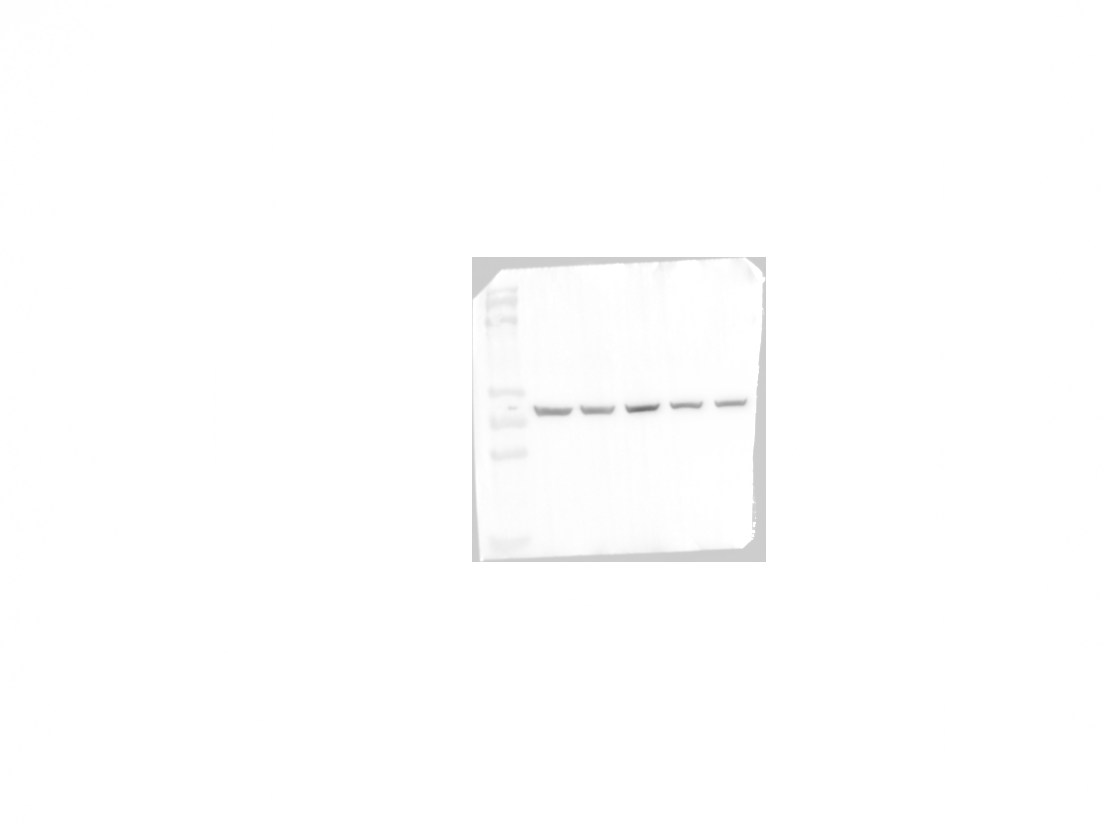

Supplement: Figure 5—source data 2. [file elife-96445-fig5-data2.zip › Figure 5_Source Data 2/5K/MGC803/PDL1actin.tif]

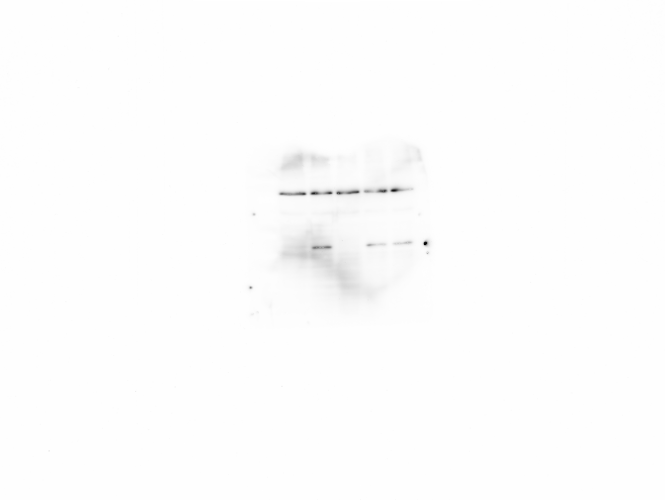

Supplement: Figure 5—source data 2. [file elife-96445-fig5-data2.zip › Figure 5_Source Data 2/5K/MGC803/spi1.tif]

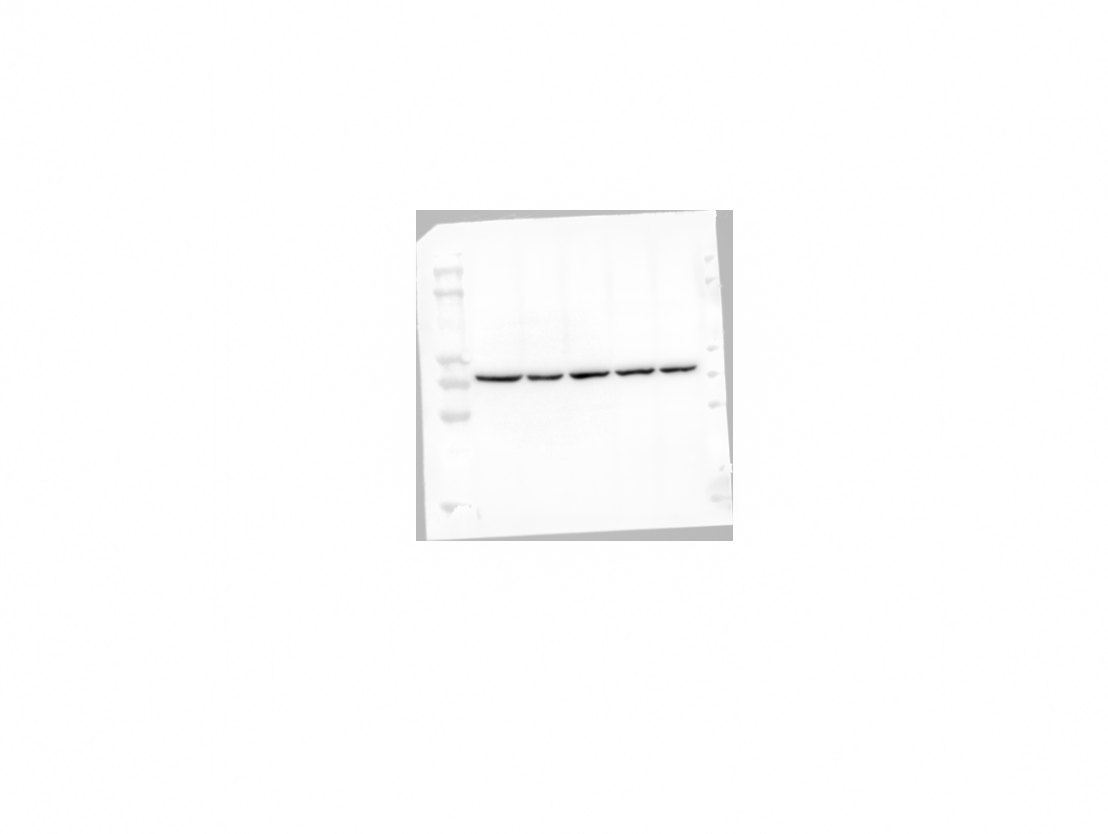

Supplement: Figure 5—source data 2. [file elife-96445-fig5-data2.zip › Figure 5_Source Data 2/5K/MGC803/SPI1ACTN.tif]

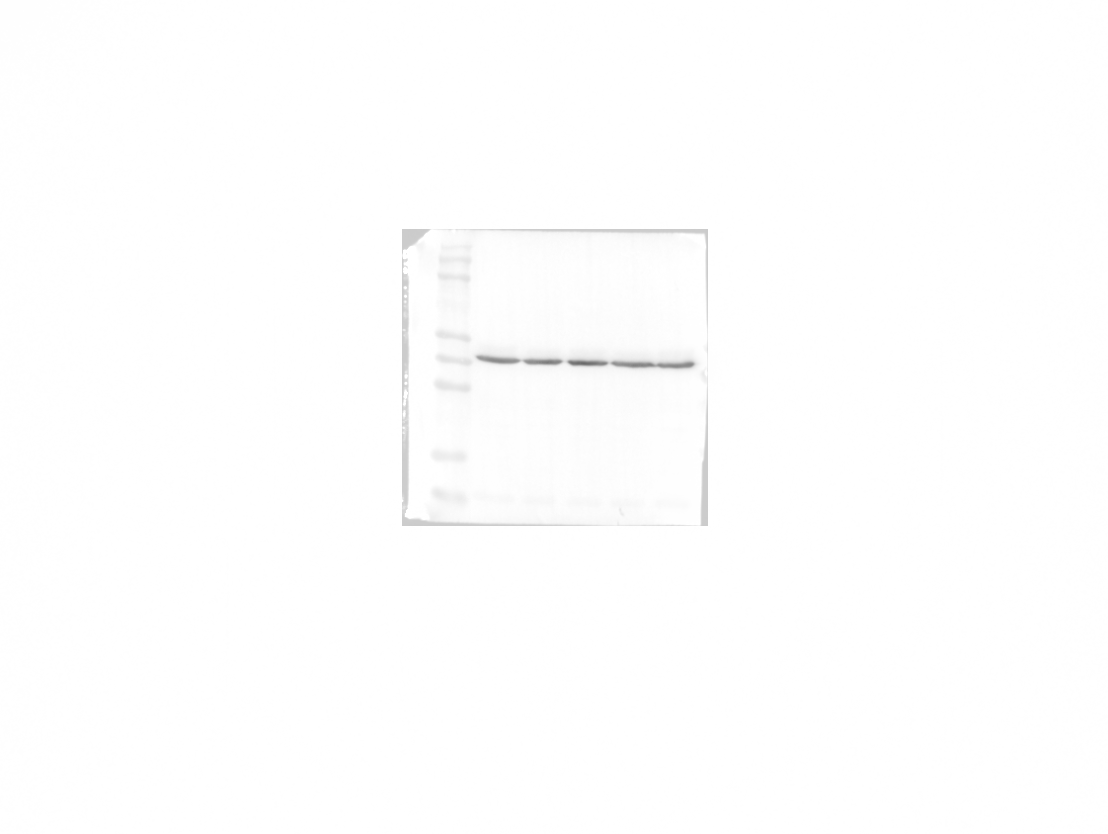

Supplement: Figure 5—source data 2. [file elife-96445-fig5-data2.zip › Figure 5_Source Data 2/5K/MKN45/HDAC3-actin.tif]

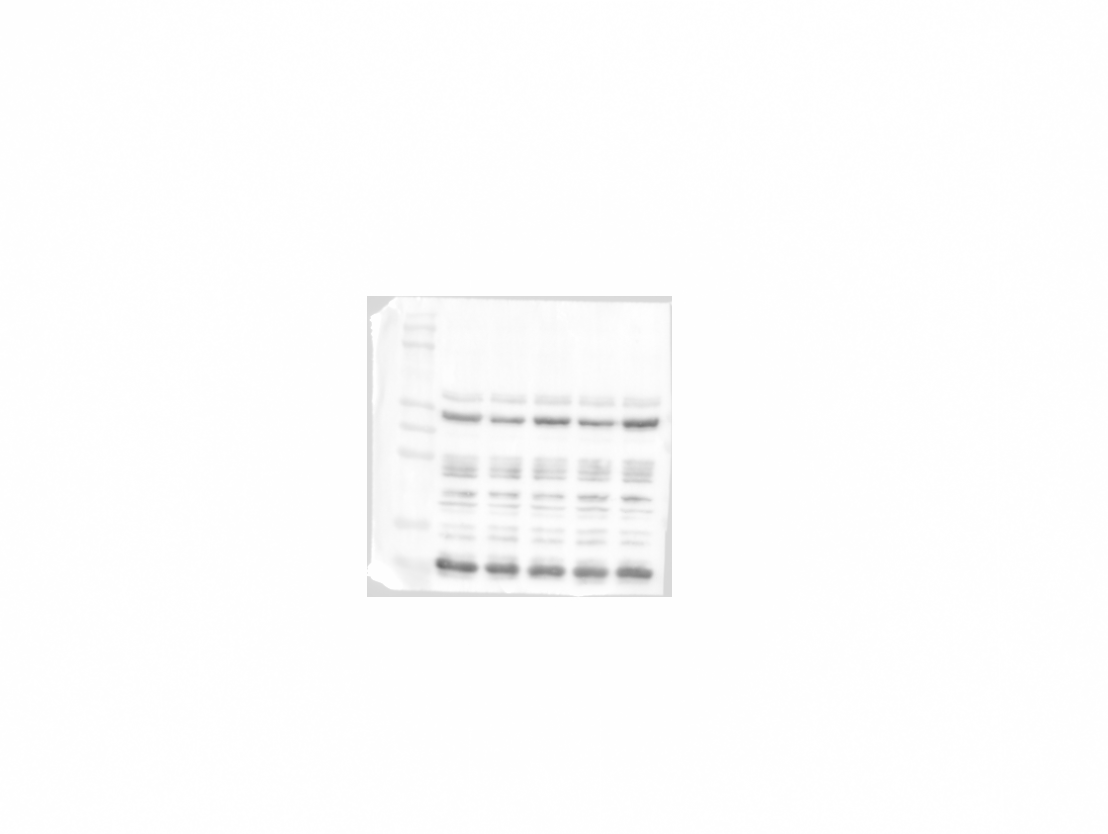

Supplement: Figure 5—source data 2. [file elife-96445-fig5-data2.zip › Figure 5_Source Data 2/5K/MKN45/HDAC3.tif]

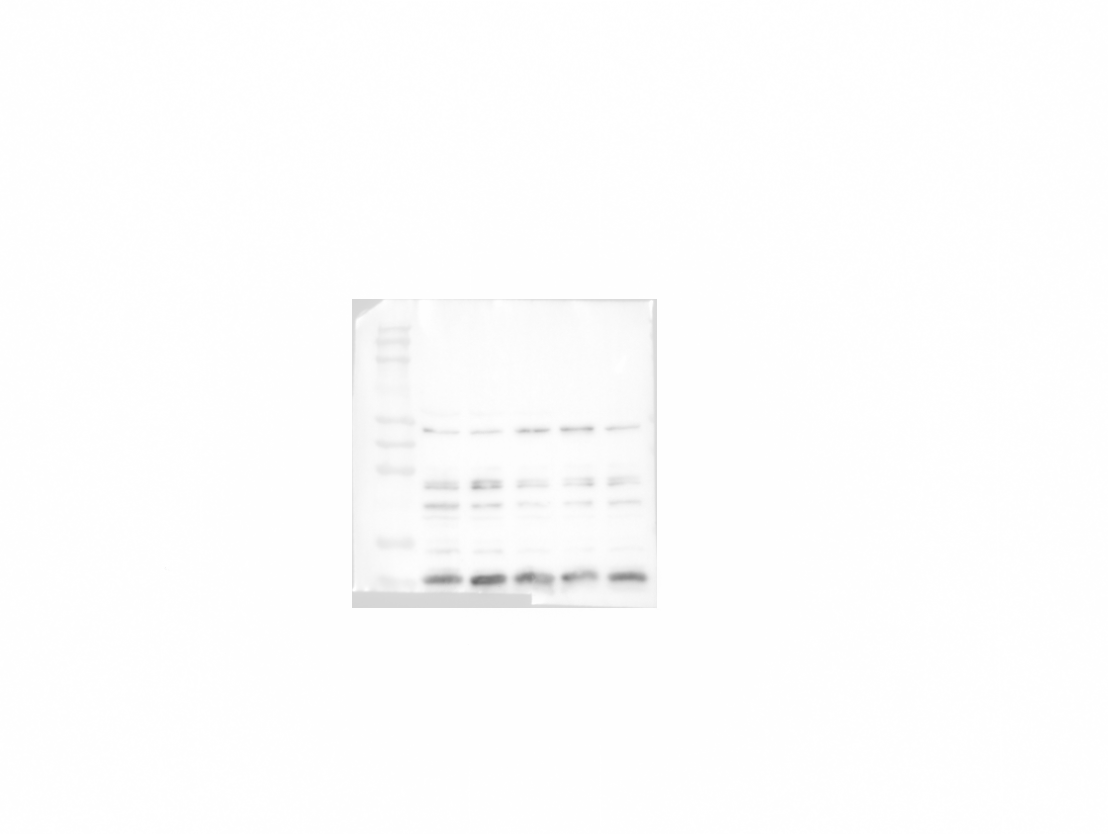

Supplement: Figure 5—source data 2. [file elife-96445-fig5-data2.zip › Figure 5_Source Data 2/5K/MKN45/pd-l1.tif]

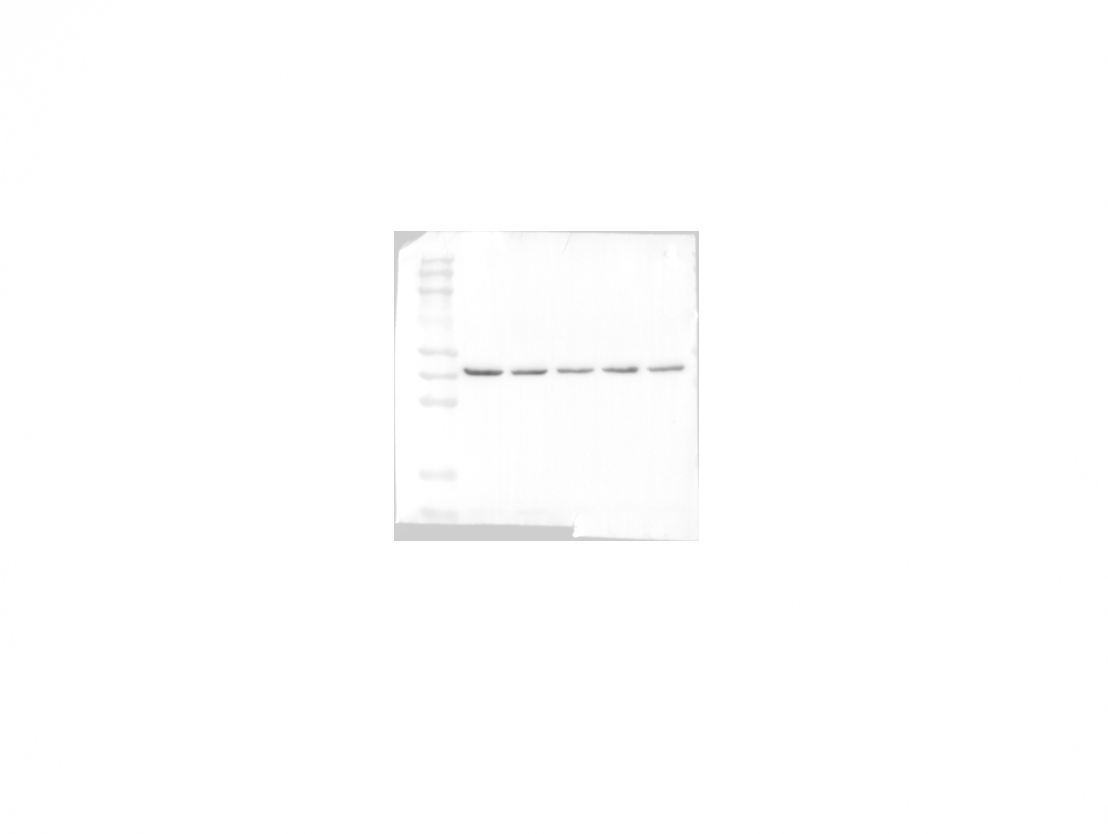

Supplement: Figure 5—source data 2. [file elife-96445-fig5-data2.zip › Figure 5_Source Data 2/5K/MKN45/pd-l1actin.tif]

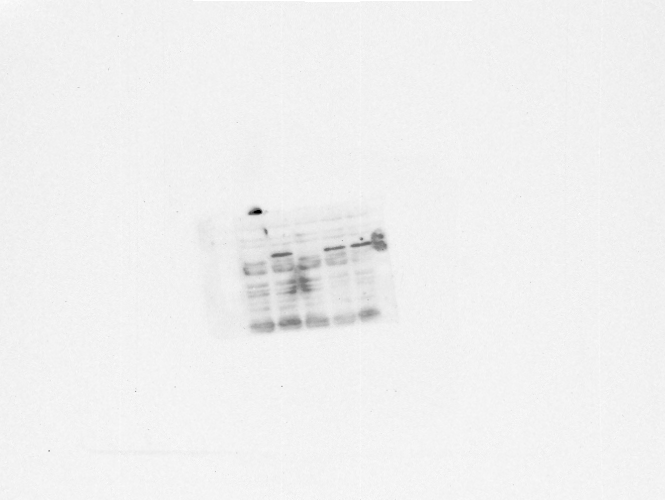

Supplement: Figure 5—source data 2. [file elife-96445-fig5-data2.zip › Figure 5_Source Data 2/5K/MKN45/spi1.tif]

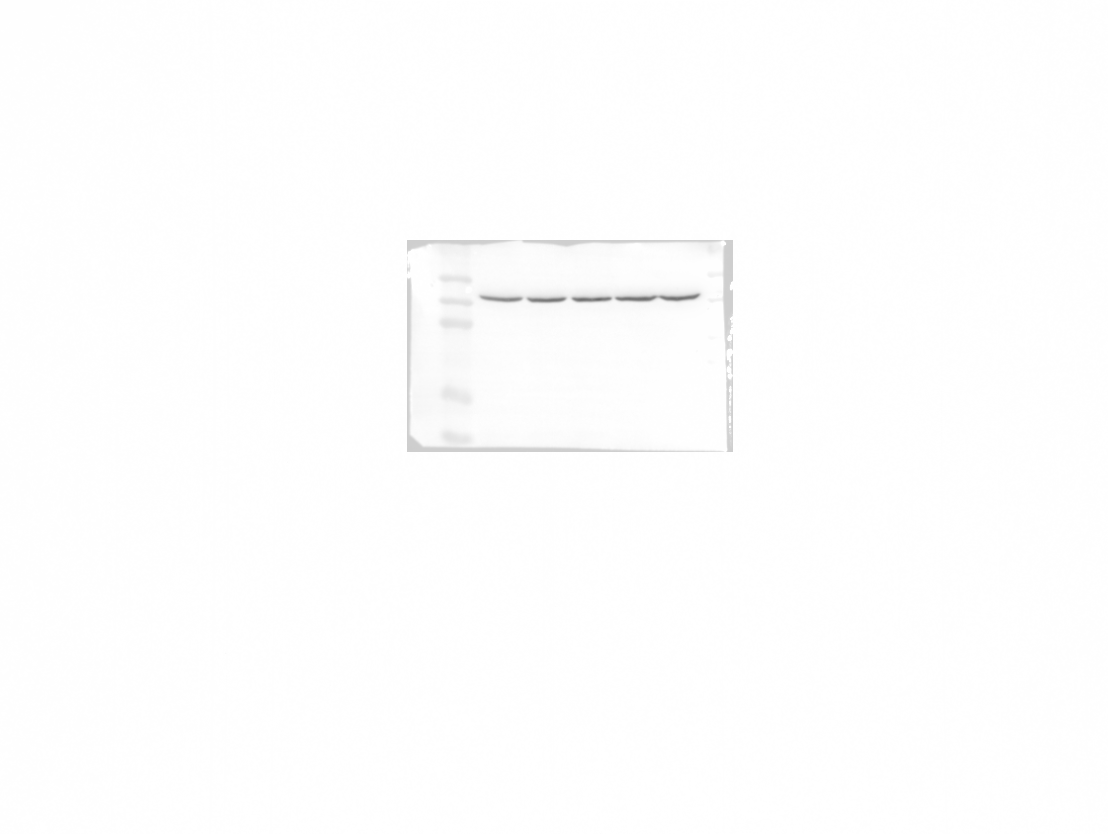

Supplement: Figure 5—source data 2. [file elife-96445-fig5-data2.zip › Figure 5_Source Data 2/5K/MKN45/spi1actin.tif]

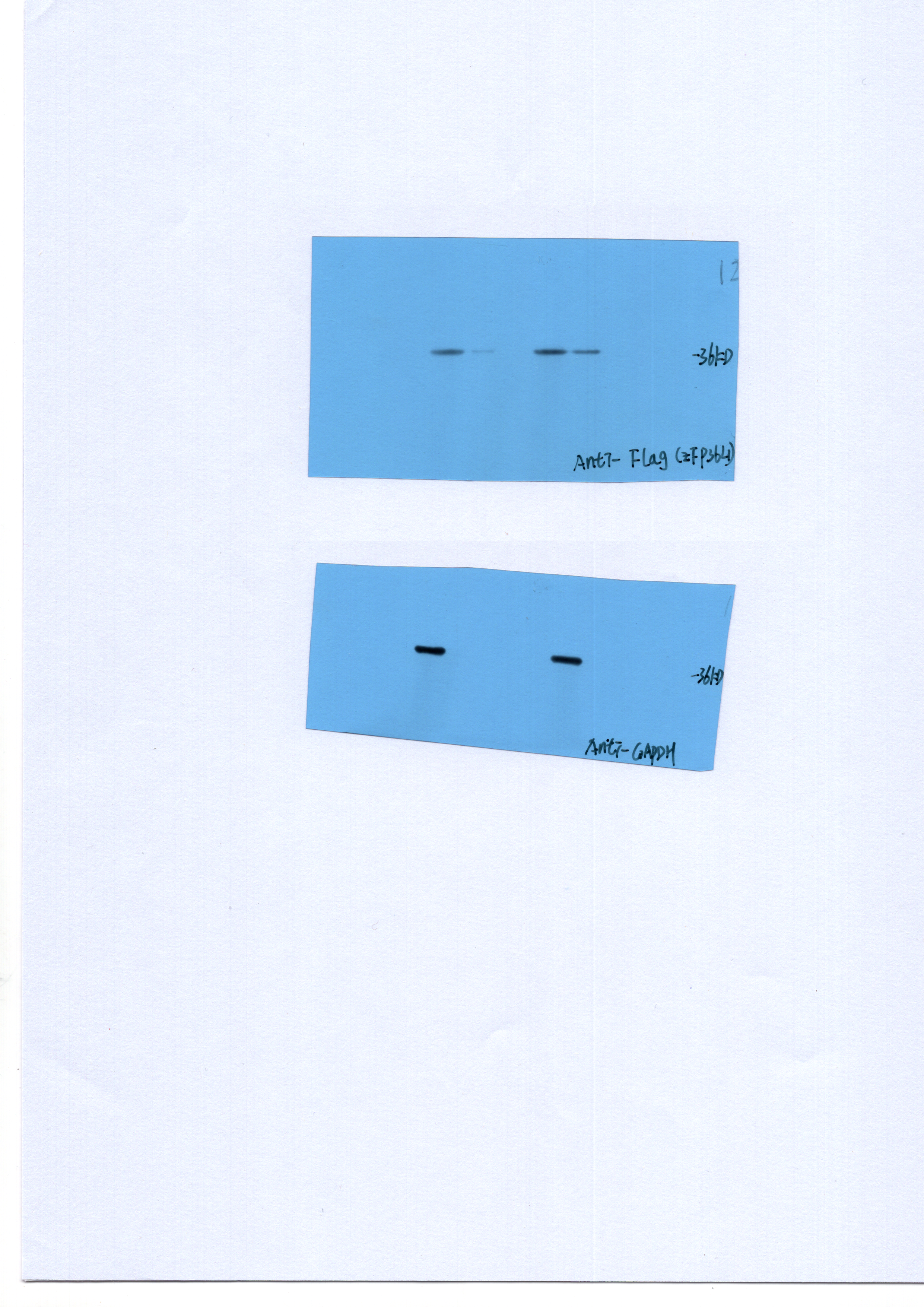

Supplement: Figure 5—source data 2. [file elife-96445-fig5-data2.zip › Figure 5_Source Data 2/5P/1.bmp]
