# Supplementary material for: Super-enhancer-driven ZFP36L1 promotes PD-L1 expression in infiltrative gastric cancer
Source: eLife. 2024 Oct 7;13:RP96445. doi: 10.7554/eLife.96445 (PMC11458174; doi:10.7554/eLife.96445)
Supplement: Figure 5—source data 1. [file elife-96445-fig5-data1.pdf]

Fig.5C

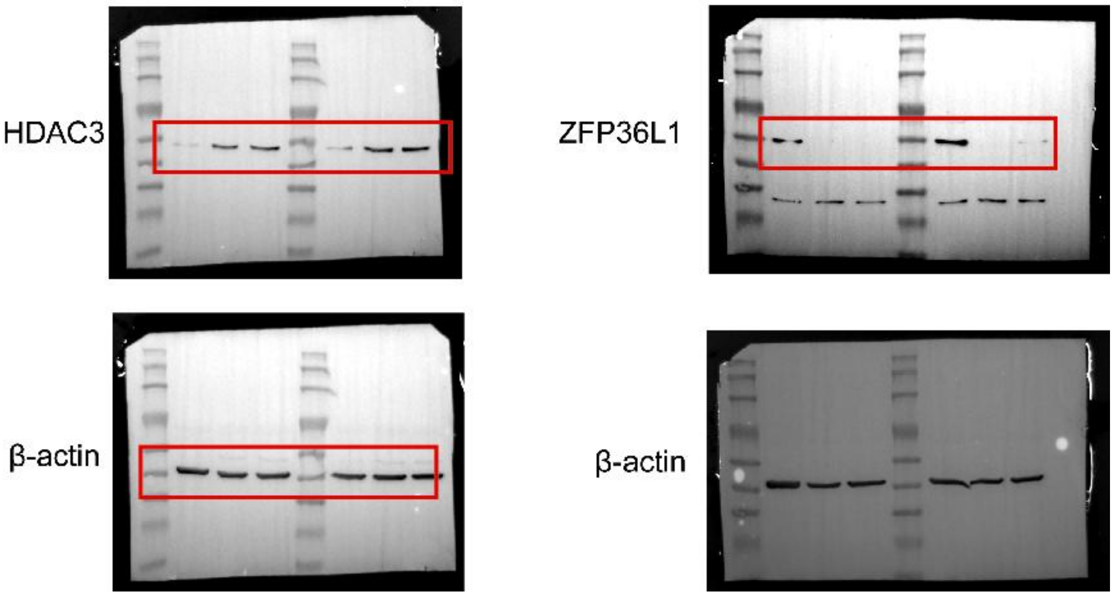

Fig.5E

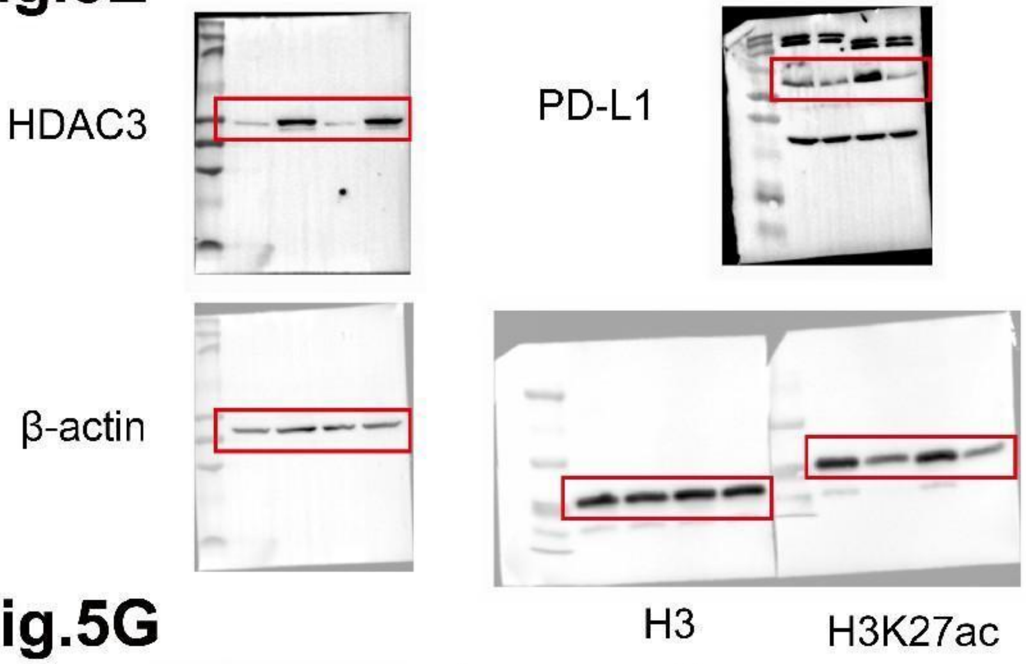

Fig.5G

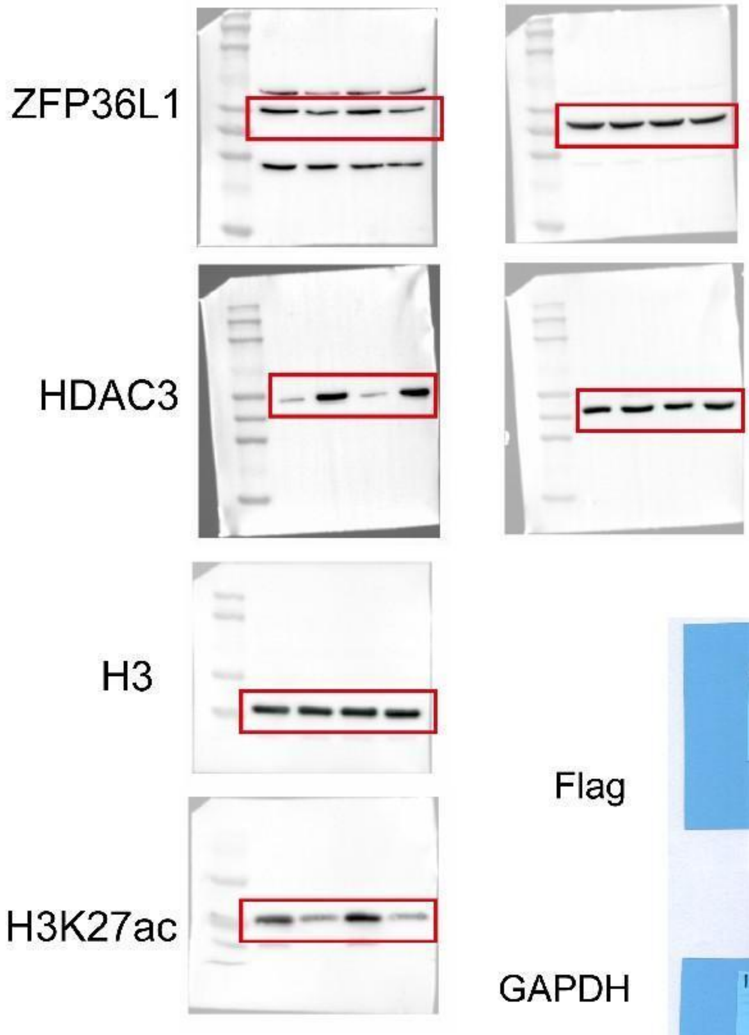

Fig.5P

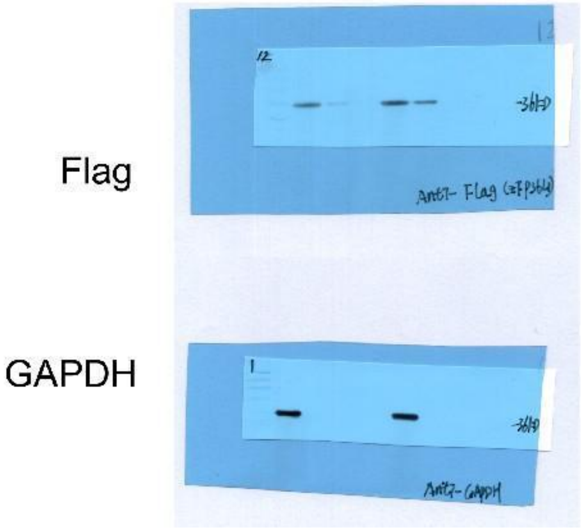

Fig.5I

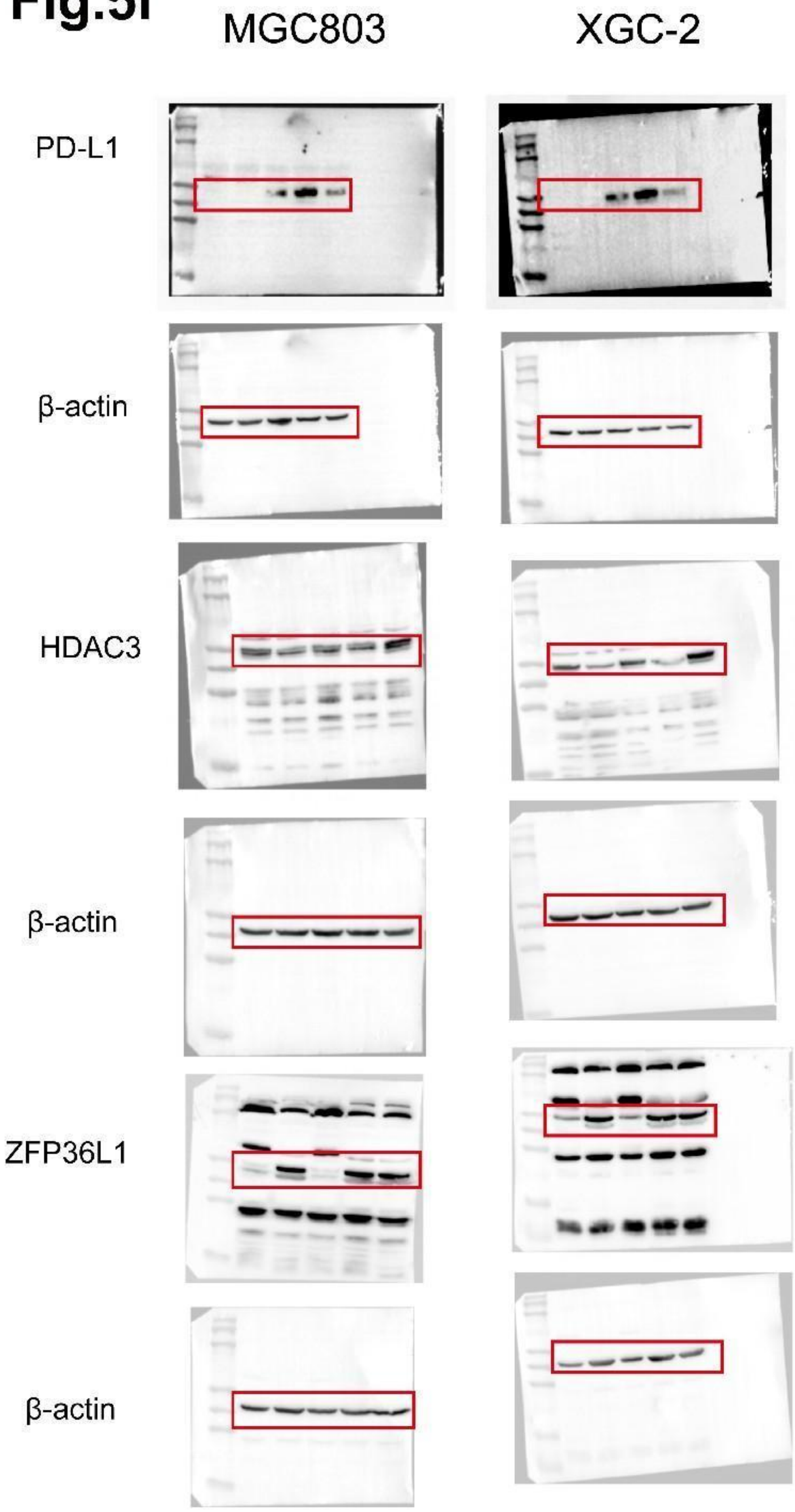

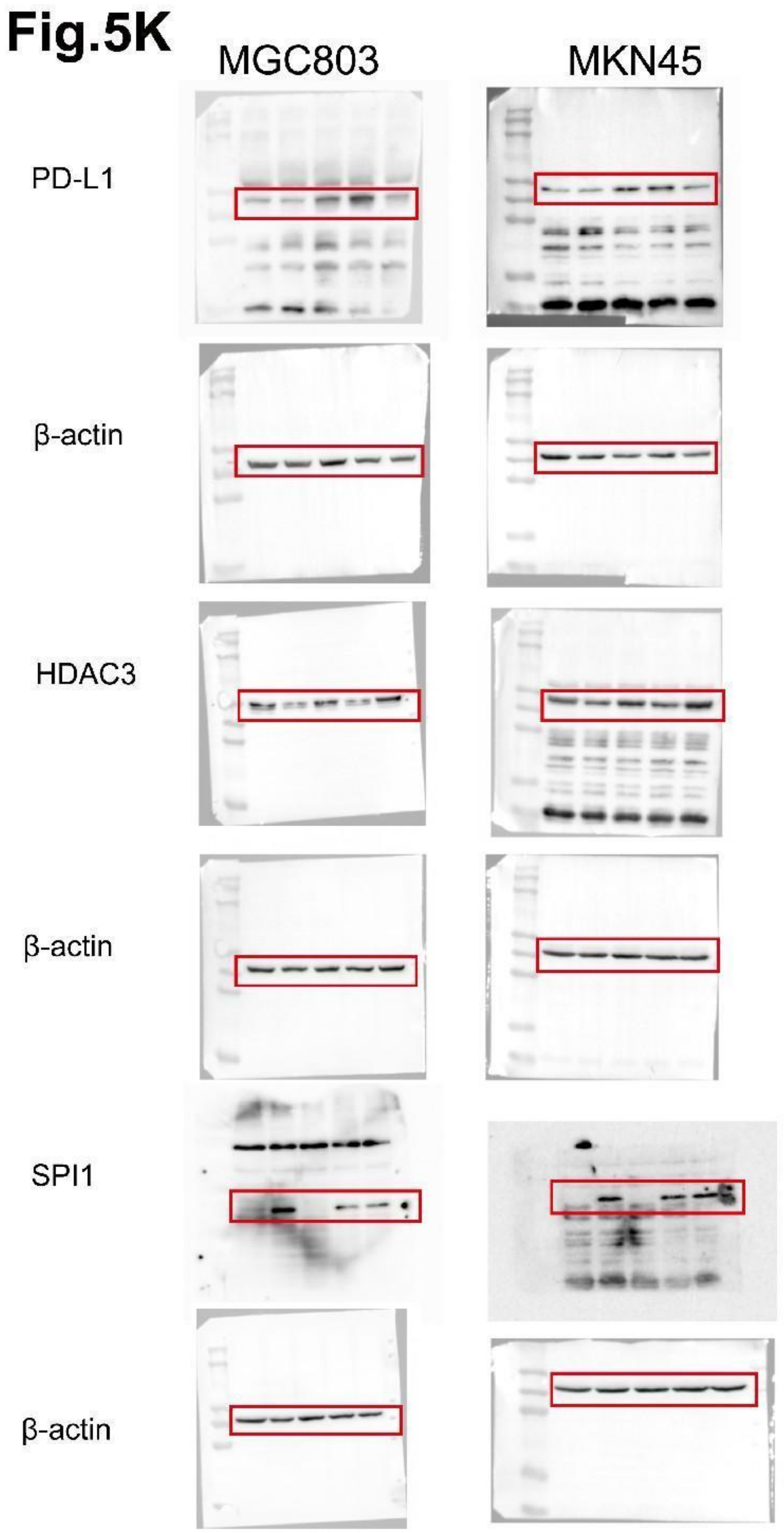

Figure 5, Source Data 1. Original blots corresponding to Figure 5
