# Supplementary figures and images for: Super-enhancer-driven ZFP36L1 promotes PD-L1 expression in infiltrative gastric cancer (part 2 of 2)
Source: eLife. 2024 Oct 7;13:RP96445. doi: 10.7554/eLife.96445 (PMC11458174; doi:10.7554/eLife.96445)

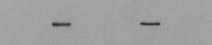

Supplement: Figure 5—source data 2. [file elife-96445-fig5-data2.zip › Figure 5_Source Data 2/5P/1.tif]

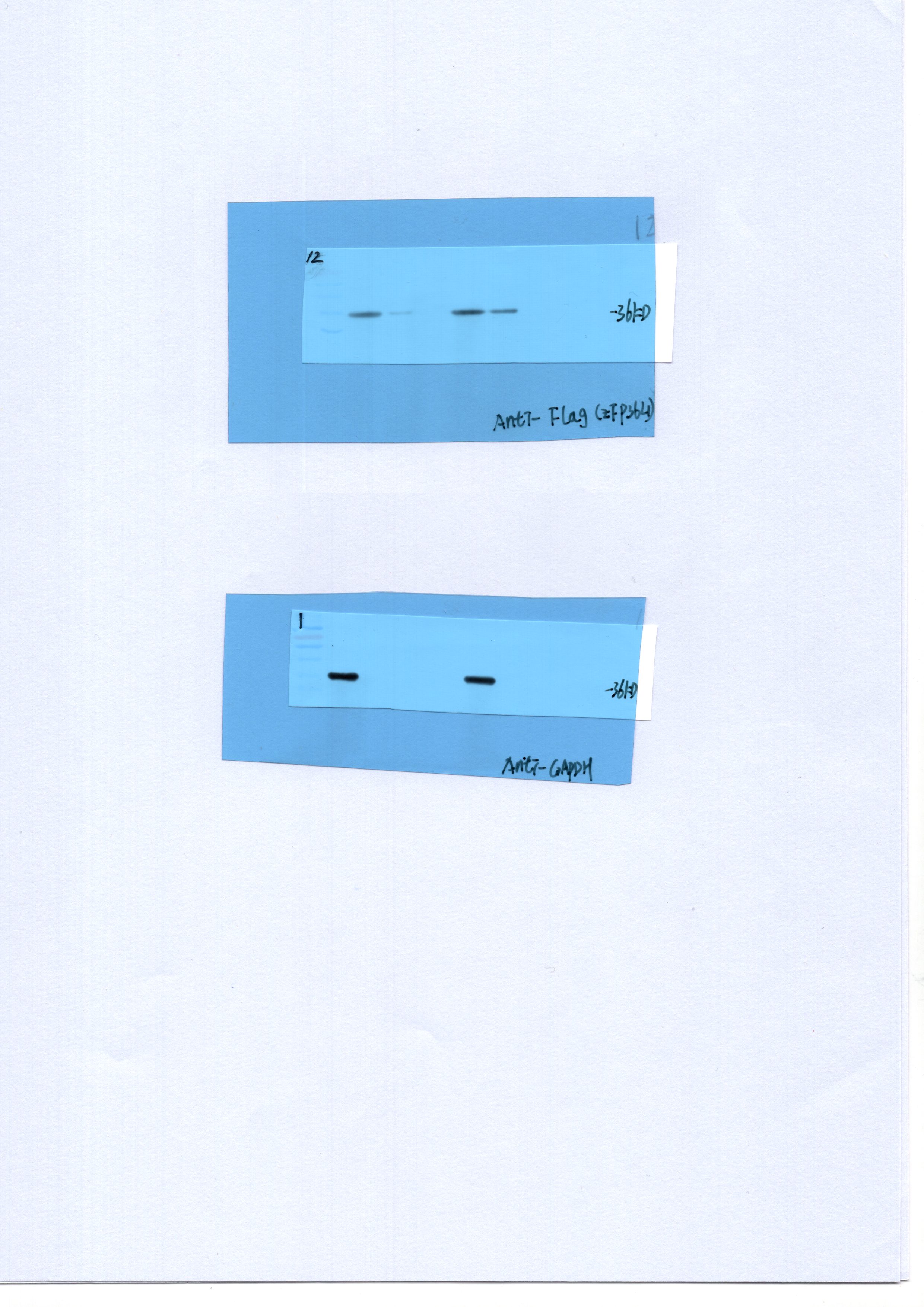

Supplement: Figure 5—source data 2. [file elife-96445-fig5-data2.zip › Figure 5_Source Data 2/5P/2.jpg]

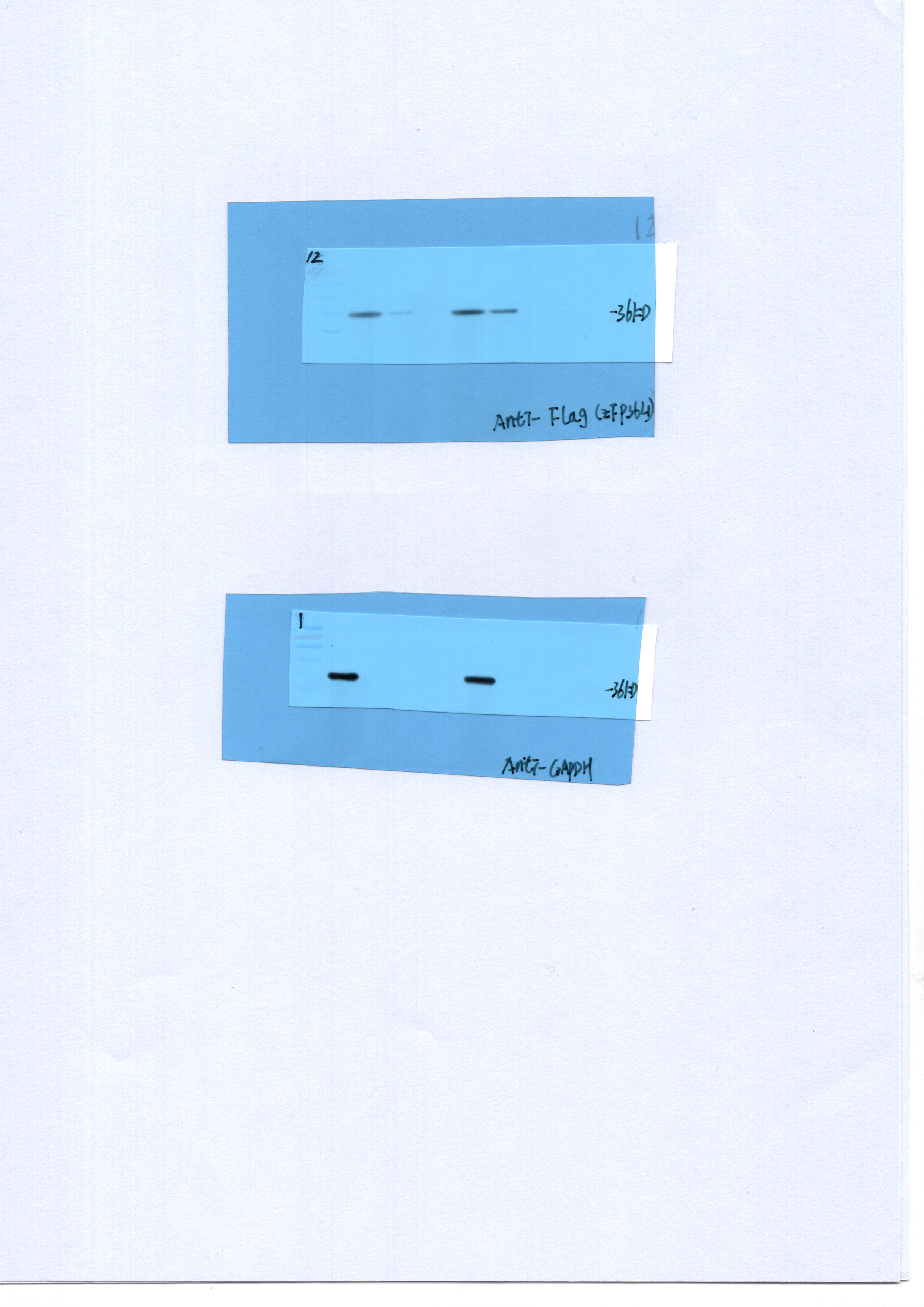

Supplement: Figure 5—source data 2. [file elife-96445-fig5-data2.zip › Figure 5_Source Data 2/5P/2.tiff]

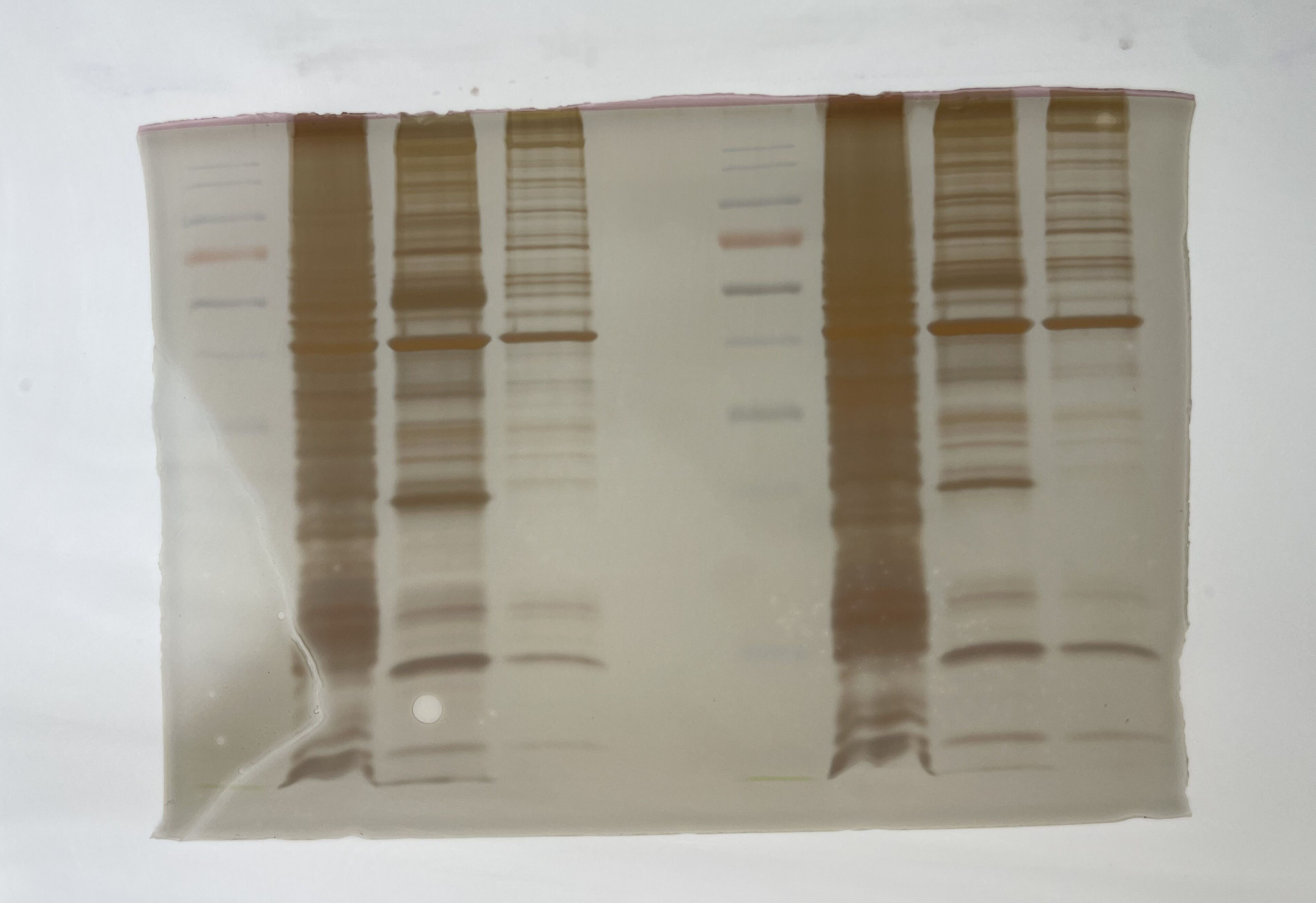

Supplement: Figure 5—source data 2. [file elife-96445-fig5-data2.zip › Figure 5_Source Data 2/5P/银染检测1.jpg]

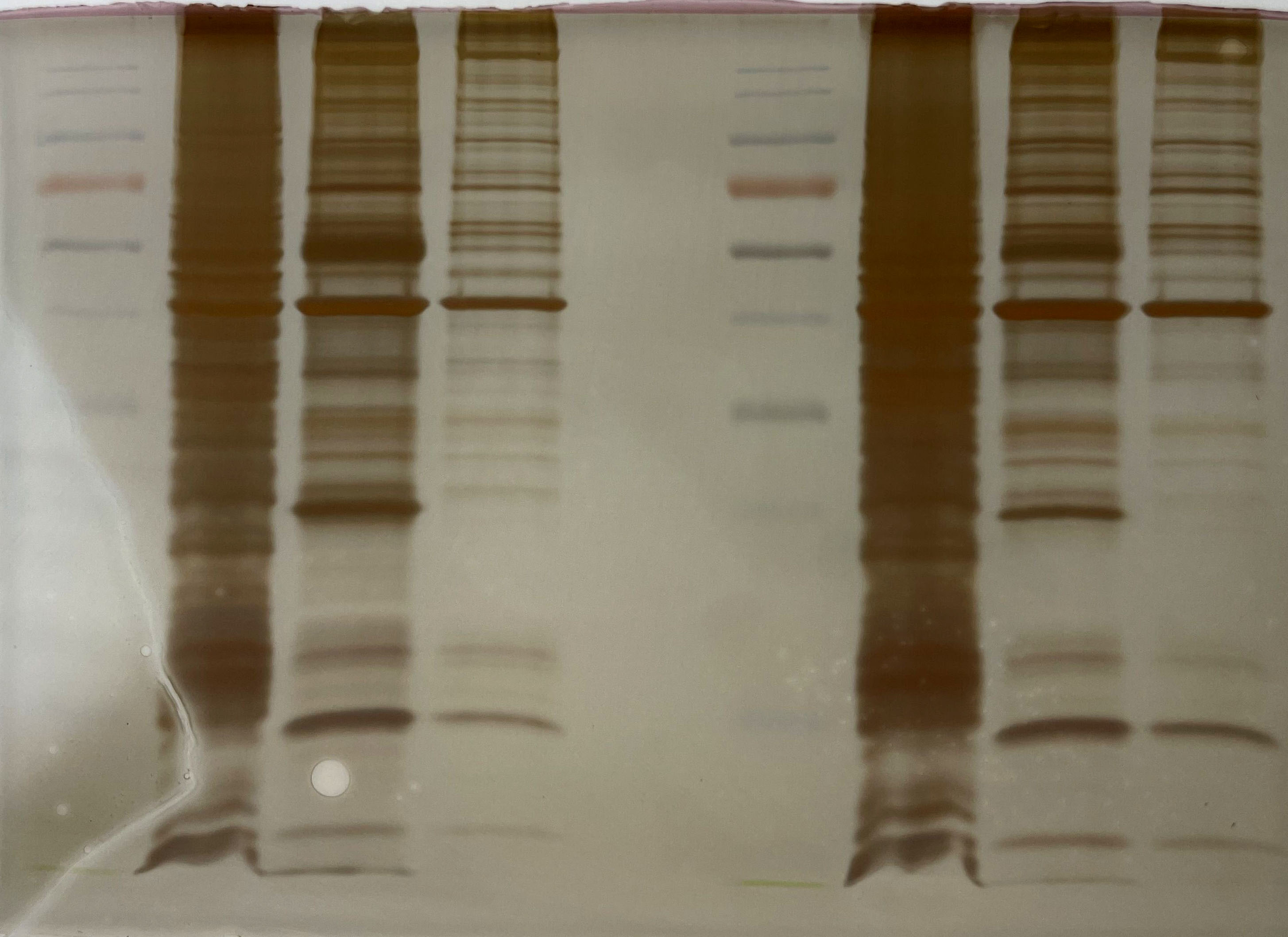

Supplement: Figure 5—source data 2. [file elife-96445-fig5-data2.zip › Figure 5_Source Data 2/5P/银染检测2.jpg]
