# Supplementary material for: Super-enhancer-driven ZFP36L1 promotes PD-L1 expression in infiltrative gastric cancer
Source: eLife. 2024 Oct 7;13:RP96445. doi: 10.7554/eLife.96445 (PMC11458174; doi:10.7554/eLife.96445)
Supplement: Supplementary file 1. — (A) Primer sequences and (B) plasmids used in this study. [file elife-96445-supp1.docx]

**1.Supplementary File 1A**

| GENE | 5’ to 3’ |
| --- | --- |
| GAPDH | Forward: ACAACTTTGGTATCGTGGAAGG  Reverse: GCCATCACGCCACAGTTTC |
| ZFP36L1 | Forward: ACCACCACCCTCGTGTCTG  Reverse: TGCCCACTGCCTTTCTGT |
| CD274 | Forward: TGGCATTTGCTGAACGCATTT  Reverse: TGCAGCCAGGTCTAATTGTTTT |
| HDAC3 | Forward: CCTGGCATTGACCCATAGCC  Reverse: CTCTTGGTGAAGCCTTGCATA |
| SPI1 | Forward: ATGGAAGGGTTTCCCCTCGT  Reverse: CTGGAGCTCCGTGAAGTTGT |
| 18S rRNA | Forward: GTCTGTGATGCCCTTAGATG  Reverse: AGCTTATGACCCGCACTTAC |
| Zfp36l1 | Forward: CACCCCAAGTACAAGACGGA  Reverse: GCTAGGAGCAAAGAGGCTCG |
| Cd274 | Forward: GCTCCAAAGGACTTGTACGTG  Reverse: TGATCTGAAGGGCAGCATTTC |
| ChIP-ZFP36L1-E1A | Forward: AAGTGCCAGTTTTCTTCCTTG  Reverse: CACCAGTCCCTGCCAGTC |
| ChIP-ZFP36L1-E1B | Forward: CTAGCAAGGCCCTGGTATG  Reverse: CTGTCCACATGGCAACCCT |
| ChIP-ZFP36L1-E1C | Forward: TTATACAACGTGGTGCTGGTG  Reverse: GTGTCAGTGCCTCCTCATT |
| ChIP-ZFP36L1-E1D | Forward: GGAGGCACTGACACGGACA  Reverse: GAATTCAAGTGGGGATTAGG |
| ChIP-CD274-P1 | Forward: GCTGGGCCCAAACCCTATT  Reverse: TTTGGCAGGAGCATG GAGTT |
| ChIP-CD274-P2 | Forward: ATGGGTCTGCTGCTGACTTT  Reverse: GGCGTCCCCCTTTCTGATAA |
| ChIP-CD274-P3 | Forward: ACTGAAAGCTTCCGCCGATT  Reverse: CCCAAGGCAGCAAAT CCAGT |

**2.Supplementary File 1B**

| Type | Plasmid DNA | remark |
| --- | --- | --- |
| ZFP36L1  (Human) | pLenti-CMV-ZFP36L1-GFP-Puro |  |
|  | pcDNA3.1-ZFP36L1-Flag |  |
|  | pPLK-GFP-Puro-ZFP36L1 shRNA-1 | GTAACAAGATGCTCAACTATA |
|  | pPLK-GFP-Puro-ZFP36L1 shRNA-2 | CCTCCAGCATAGCTTTAGCTT |
|  | pcDNA3.1-mutZFP36L1-Flag | C153R-C173R |
| ZFP36L1  (Mouse) | pLVX-CMV-Zfp36l1-3×Flag-Puro |  |
|  | pLKO.1-U6-Zfp36l1 shRNA-1-EF1a-copGFP-T2A-Puro | GCTTTCGAGACCGCTCTTTCTC |
|  | pLKO.1-U6-Zfp36l1 shRNA-2-EF1a-copGFP-T2A-Puro | GCTGCCACTTCATTCATAACGC |
|  | pLenti-CMV-SPI1-GFP-Puro | ID：6688；NM_001080547.2 |
|  | pLenti-CMV-ELF1-GFP-Puro | ID：1997；NM_172373.4 |
|  | pLenti-CMV-E2F1-GFP-Puro | ID：1869；NM_005225.3 |
|  | pcDNA3.1-BRD4-3×Flag | ID: 23476；NM_058243.3 |
|  | pLVX-Puro-Flag-HDAC3 | ID: 8841；NM_001355039.2 |
| prokaryotic expression | pGEX-4T-2-GST-SPI1 |  |
|  | pET-32a-His-BRD4 |  |
| Luciferase reporter | pGL4-Luci-E1 (Full length) | chr14:68806839-68807740 |
|  | pGL4-Luci-E1A | chr14:68806839-68807000 |
|  | pGL4-Luci-E1B | chr14:68807000-68807300 |
|  | pGL4-Luci-E1C (Wild) | chr14:68807300-68807500 |
|  | pGL4-Luci-E1C (Deletion) | deletion: GAAGAGGGAAGGCAG, chr14:68807469-68807483 |
|  | pGL4-Luci-E1D | chr14:68807500-68807740 |
|  | pGL4-Luci-CD274 promoter |  |
|  | pmirGLO-HDAC3-3’UTR (Wild) | “ATTTA” motif |
|  | pmirGLO-HDAC3-3’UTR (Mutant) | “ACCCA” mutant motif |
